# Adipocytes promote malignant growth of breast tumours with monocarboxylate transporter 2 expression via β-hydroxybutyrate

**DOI:** 10.1038/ncomms14706

**Published:** 2017-03-10

**Authors:** Chun-Kai Huang, Po-Hao Chang, Wen-Hung Kuo, Chi-Long Chen, Yung-Ming Jeng, King-Jen Chang, Jin-Yuh Shew, Chun-Mei Hu, Wen-Hwa Lee

**Affiliations:** 1 Institute of Biochemistry and Molecular Biology, College of Medicine, National Taiwan University, Taipei 100, Taiwan; 2 Genomics Research Center, Academia Sinica, Taipei 115, Taiwan; 3 Department of Surgery, National Taiwan University Hospital, Taipei 100, Taiwan; 4 Department of Pathology, Wan Fang Hospital, Taipei Medical University, Taipei 116, Taiwan; 5 Department of Pathology, National Taiwan University Hospital, Taipei 100, Taiwan; 6 Cheng Chin General Hospital, Taichung 407, Taiwan; 7 Graduate Institute of New Drug Development, China Medical University, Taichung 404, Taiwan

## Abstract

Adipocytes are the most abundant stromal partners in breast tissue. However, the crosstalk between breast cancer cells and adipocytes has been given less attention compared to cancer-associated fibroblasts. Here we find, through systematic screening, that primary mammary gland-derived adipocytes (MGDAs) promote growth of breast cancer cells that express monocarboxylate transporter 2 (MCT2) both *in vitro* and *in vivo*. We show that β-hydroxybutyrate is secreted by MGDAs and is required to enhance breast cancer cells malignancy *in vitro*. Consistently, β-hydroxybutyrate is sufficient to promote tumorigenesis of a mouse xenograft model of MCT2-expressing breast cancer cells. Mechanistically we observe that upon co-culturing with MGDAs or treatment with β-hydroxybutyrate, breast cancer cells expressing MCT2 increase the global histone H3K9 acetylation and upregulate several tumour-promoting genes. These results suggest that adipocytes promote malignancy of MCT2-expressing breast cancer via β-hydroxybutyrate potentially by inducing the epigenetic upregulation of tumour-promoting genes.

## Abstract (web summary)

Invasion of the adipose tissue correlates with poor prognosis in breast cancer. Here, the authors show that mammary gland adipocytes promote malignancy via β-hydroxybutyrate, which acts on cancer cells through the monocarboxylate transporter MCT2 resulting in tumour-promoting epigenetic modifications.

Emerging evidence indicates that the tumour microenvironment plays a vital role in the initiation and progression of many cancers^1^. In breast cancer, cancer-associated fibroblasts and tumour-associated macrophages promote breast cancer progression and metastasis by secreting growth factors and chemokines^2^,^3^,^4^,^5^. Deciphering the underlying molecular mechanisms of crosstalk among heterotypic cells is of great interest because new targets for improving diagnosis or novel therapeutic strategies may be revealed.

Adipocytes are abundant stromal cells in human breast tissue. During breast cancer development, invasion of tumour cells through the basement membrane results in a close interaction between cancer cells and adipocytes^6^. Several clinical observations demonstrate that local invasion of the adipose tissue correlates with poor prognosis^7^,^8^, highlighting the importance of adipocytes in breast tumour progression. In addition to their function in energy metabolism and regulation of energy homeostasis, adipocytes also secrete hormone and various cytokines (also known as adipokines)^9^, which participate in cancer progression. For instance, IGF-1, leptin and interleukin-6 (IL-6) secreted from adipocytes promote breast cancer growth and invasion^10^,^11^,^12^. The expression of metalloproteinase (MMP)-11/stromelysin-3 in adipocytes is induced by breast cancer cells at the tumour invasive front, indicative of a role in extracellular matrix remodelling during breast cancer development^13^. Furthermore, it has been shown that adipocytes co-cultured with breast cancer cells exhibit decreased adiponectin concomitant with IL-6 overexpression^12^. These studies suggest there is an intimate crosstalk between adipocytes and breast cancer cells. However, the underlying mechanism of how breast cancer cells interact with mature adipocytes is not fully understood.

In this communication, we provide a systematic investigation exploring this issue for breast cancer. We establish a co-culture system using breast cancer cells and adipocytes isolated from breast cancer mastectomy specimens (called mammary gland-derived adipocytes, MGDAs) to identify novel receptor(s) involved in the crosstalk between breast cancer cells and adipocytes. Through microarray analyses and RNAi knockdown screening, monocarboxylate transporter 2 (MCT2) is identified as a new player in MGDAs-mediated promotion of tumorigenic activity. Furthermore, either MGDAs co-culture or treatment of β-hydroxybutyrate, an intrinsic histone deacetylase (HDAC) inhibitor, increases histone H3K9 acetylation and induces expressions of *IL-1β* and *LCN2* to enhance tumorigenicity in MCT2-expressing breast cancer cells. Consistently, elevated expressions of *MCT2* as well as β-hydroxybutyrate-induced genes, *IL-1β* and *LCN2*, are significantly (*P* values: *MCT2*/*IL-1β*=0.029; *MCT2*/*LCN2*=0.017, log-rank test) correlated with poor prognosis in breast cancer patients in two independent cohorts. These findings provide new insights into the connection between adipocytes and breast cancer malignancy.

## Results

### MGDAs promote growth of distinct subtypes of breast cancer

**Figure 1:**
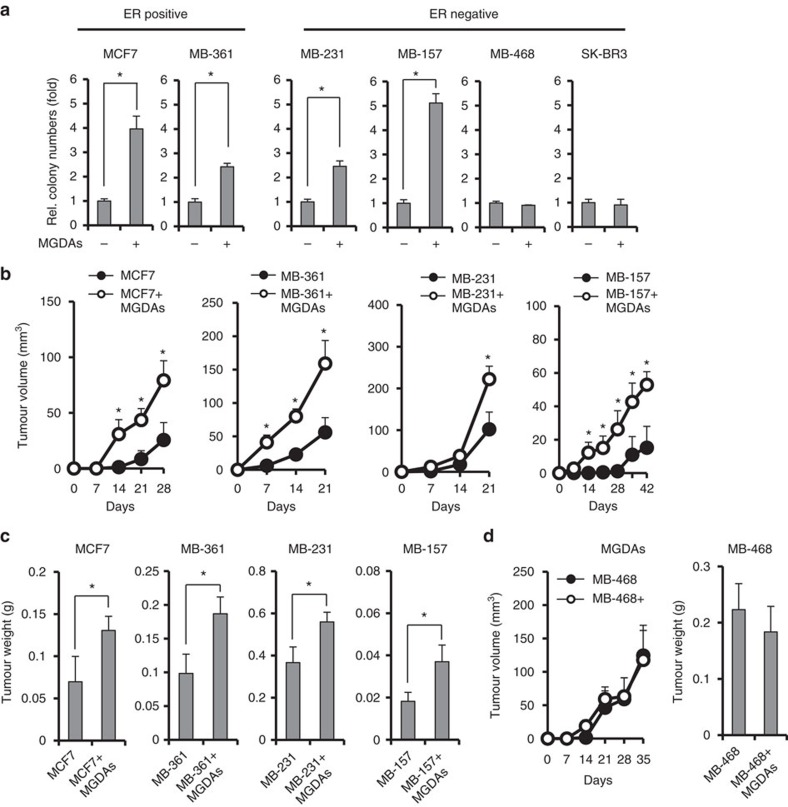
Mammary gland-derived adipocytes promote tumour growth in several breast cancer cell lines. (**a**) Soft agar colony formation assays using a panel of breast cancer cell lines co-cultured with MGDAs. (**b**) Tumour growth assays in NOD/SCID/γ^null^ mice. Breast cancer cells were subcutaneously injected in both flanks of each mouse w/ or w/o MGDAs and tumour volumes were measured every 7 days. Four mice (*n*=4) were used for each group. (**c**) Co-injection of MGDAs in xenograft models increased the tumour weights in several breast cancer cell lines. (**d**) MGDAs co-injection failed to enhance the tumour growth of MDA-MB-468 cells in mouse tumorigenicity assays. The experiment in **a** was performed in technical triplicate and repeated at least twice with similar results. In **a**–**d**, data show means±s.d. *P<0.05 (Student's *t*-test).

To address whether MGDAs play a role in promoting breast tumour malignancy, a panel of estrogen receptor (ER)-positive (MCF7 and MDA-MB-361) and ER-negative (MDA-MB-231, MDA-MB-157, MDA-MB-468 and SK-BR3) breast cancer cell lines were used for soft agar colony formation assays with MGDAs co-cultured in the bottom layer. In this co-culture system, MGDAs and breast cancer cells are separated, but crosstalk can occur through diffusible factors. Human primary MGDAs were isolated from breast cancer specimens after mastectomy according to published protocols^14^ (Supplementary Fig. 1A,B). As shown in Fig. 1a, MGDAs promoted colony formation of MCF7, MDA-MB-361, MDA-MB-231 and MDA-MB-157 cells, but not MDA-MB-468 and SK-BR3 cells. No strict correlation with the ERα status of the breast cancer cell lines was noted. Similar results were also obtained using two independent batches of MGDAs isolated from different breast cancer patients, suggesting that this MGDAs activity is not patient-specific. The colony number increased when greater numbers of MGDAs were used in the co-culture system (Supplementary Fig. 1C). Next, we examined the tumour formation ability of breast cancer cells co-cultured with MGDAs in a mouse xenograft tumour model. MGDAs and breast cancer cells were co-implanted subcutaneously instead of in the fat pad to minimize potential interference by mouse adipocytes. As shown in Fig. 1b,c, the MCF7, MDA-MB-361, MDA-MB-231 and MDA-MB-157 cells mixed with MGDAs grew faster and had greater tumour weights than the control groups. However, little or no difference was observed with the MDA-MB-468 cells (Fig. 1d). Taken together, these data suggest that promotion of breast cancer malignancy by MGDAs is selectively dependent on breast cancer cell type, with the effects likely mediated by soluble factors secreted from the MGDAs.

### Identifying membrane proteins mediating responses to MGDAs

**Figure 2:**
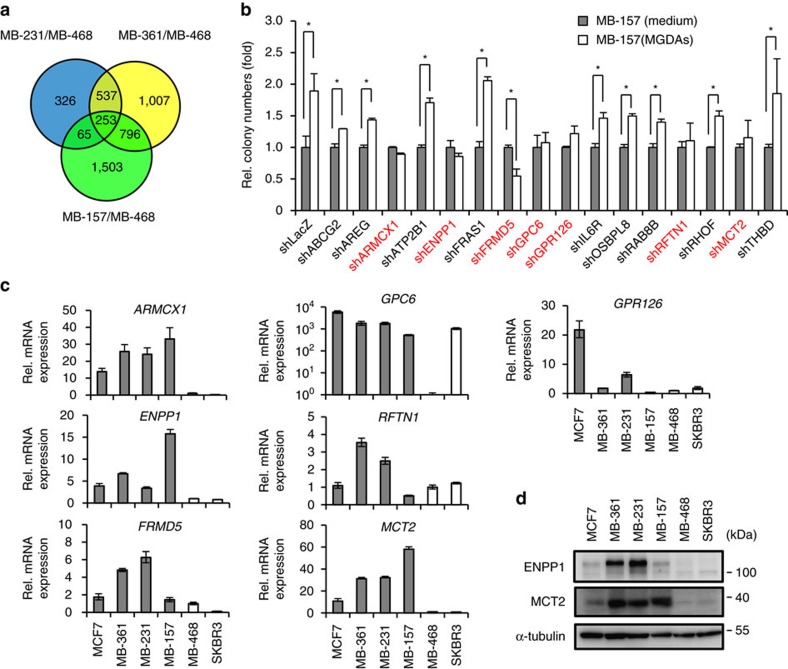
Identification of membrane proteins involved in MGDAs-mediated enhancement of tumorigenic activity. (**a**) Summary of cDNA microarray analyses. In all, 253 genes were identified with at least twofold upregulation in MDA-MB-231, MDA-MB-361 and MDA-MB-157 compared to MDA-MB-468 cells. (**b**) RNAi knockdown screening of candidate genes involved in MGDAs-mediated tumorigenic activity promotion in MDA-MB-157 cells by soft agar colony formation assay. shLacZ was used as a knockdown control. (**c**) Expression profiles of *ARMCX1*, *ENPP1*, *FRMD5*, *GPC6*, *GPR126*, *RFTN1* and *MCT2* in six different breast cancer cell lines. The experiment was performed in technical triplicate. (**d**) Western blot analyses reconfirmed the expression profiles of ENPP1 and MCT2 in breast cancer cell lines (no commercial workable ARMCX1 antibody was found). The experiment in **b** was performed in technical triplicate and repeated at least twice with similar results. Data show means±s.d. *P<0.05 (Student's *t*-test).

To explore potential factors of breast cancer cells involved in MGDAs-mediated tumorigenic enhancement, we compared the gene expression profiles of MDA-MB-231, MDA-MB-361, MDA-MB-157 and MDA-MB-468 cells from previous cDNA microarray results^15^ (Fig. 2a and Supplementary Data 1). Genes upregulated twofold or more in the MDA-MB-231, MDA-MB-361 and MDA-MB-157 cell lines, when compared to MDA-MB-468, were selected as candidate genes. Among the 253 genes identified, 16 encoding membrane or membrane-associated proteins were selected for further study as these receptors/transporters may interact with diffusible factors from adipocytes (Table 1). Q-PCR analysis confirmed that the expression profiles of these genes were consistent with the results from the cDNA microarray (Fig. 2c and Supplementary Fig. 2A). Next, we individually depleted each of these 16 candidate genes in MDA-MB-157 cells by pooled RNAi knockdown to evaluate their roles in MGDAs-mediated tumour enhancement (Supplementary Fig. 2B). Depletion of *ARMCX1*, *ENPP1*, *FRMD5*, *GPC6*, *GPR126*, *RFTN1* and *MCT2* abolished the colony number increase induced by co-culture with MGDAs (Fig. 2b). However, only *ARMCX1*, *ENPP1* and *MCT2* showed a better correlation between phenotype and expression level as high expression of these three genes was detected in MCF7, MDA-MB-361, MDA-MB-231 and MDA-MB-157 cells (for which MGDAs had a promoting activity) while low expression was found in MDA-MB-468 and SKBR3 cells (for which MGDAs co-culture had no significant effect) (Fig. 2c,d).

**Figure 3:**
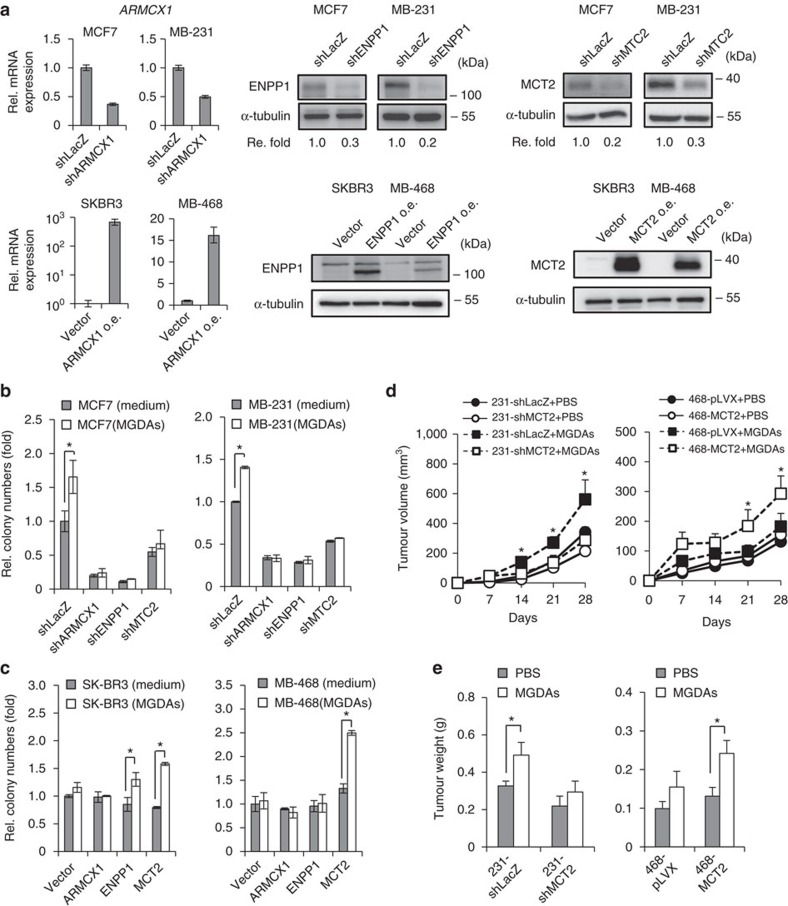
MCT2 is required for the enhancement of tumorigenic activity associated with MGDAs. (**a**) Q-PCR and western blot analyses of ARMCX1, ENPP1 and MCT2 depletion and ectopic overexpression in breast cancer cells. (**b**) Soft agar colony formation assays showed that depletion of ARMCX1, ENPP1 and MCT2 abrogated the increase of colonies induced by MGDAs co-culture in MCF7 and MDA-MB-231 cells. (**c**) Soft agar colony formation assays of ARMCX1, ENPP1 and MCT2 overexpressing MDA-MB-468 and SK-BR3 cells w/ or w/o MGDAs co-culture. (**d**) Tumour growth assays in NOD/SCID/γ^null^ mice. MCT2-depleted MDA-MB-231 and overexpressing MDA-MB-468 cells were subcutaneously injected in both flanks of each mouse w/ or w/o MGDAs. Tumour volumes were measured every 7 days. Four mice (*n*=4) were used for each group. (**e**). Tumour weights were increased in the presence of MCT2 after co-injection with MGDAs in mouse tumorigenicity assays. The experiments in **b**,**c** were performed in technical triplicate and repeated at least twice with similar results. In **b**–**e**, data show means±s.d. *P<0.05 (Student's *t*-test).

To further verify that ARMCX1, ENPP1 and MCT2 contribute to MGDAs-mediated effects, we depleted *ARMCX1*, *ENPP1* and *MCT2* expression in two additional cell lines (MCF7 and MDA-MB-231) and obtained similar results (Fig. 3a,b). However, we also observed that depletion of *ARMCX1*, *ENPP1* and *MCT2* inhibited the colony formation (Fig. 3b). To exclude the possibility that ARMCX1, ENPP1 and MCT2 are essential for breast cancer growth regardless of the MGDAs, we tested whether colony formation was enhanced by MGDAs in MDA-MB-468 and SK-BR3 cells ectopically expressing these genes (Fig. 3a). As shown in Fig. 3c only MCT2, but not ARMCX1 nor ENPP1, consistently enhanced colony formation in both MDA-MB-468 and SK-BR3 cells. Subcutaneous co-injection of MCT2-depleted MDA-MB-231 cells or MCT2-overexpressing MDA-MB-468 cells with MGDAs in NOD/SCID/γ^null^ mice reduced or accelerated tumour growth compared to the control groups, respectively (Fig. 3d,e). Taken together, these results from loss- and gain-of-function analyses suggest that the presence of MCT2 in breast cancer cells mediates the tumorigenic effect promoted by MGDAs.

### β-hydroxybutyrate enhances malignancy of breast cancer cells

**Figure 4:**
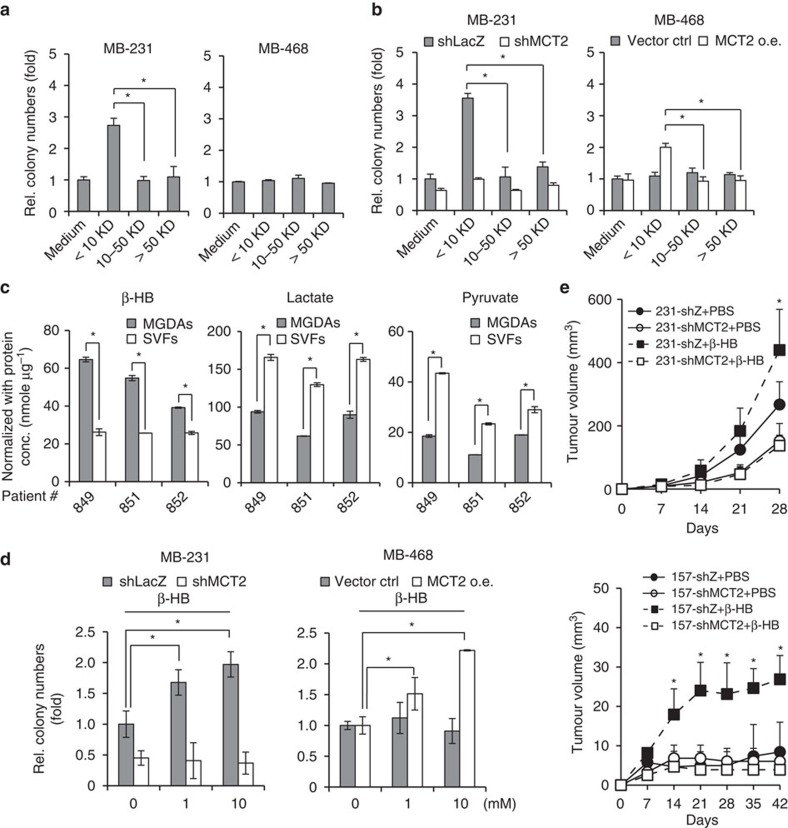
β-hydroxybutyrate from MGDAs promotes breast cancer progression. (**a**) Soft agar colony formation assays of MDA-MB-231 and MDA-MB-468 cells treated with conditioned medium collected from MGDAs culture. The MGDAs-conditioned medium was fractionated by ultrafiltration into <10 kD, 10–50 kD and >50 kD fractions. (**b**) Depletion or overexpression of MCT2 in MDA-MB-231 or MDA-MB-468 cells either abrogated or enhanced the susceptibility to treatment with fractionated MGDAs-conditioned medium. (**c**) Secretion levels of β-hydroxybutyrate, lactate and pyruvate in conditioned medium from MGDAs and stromal vascular fraction (SVF) cells were determined by ELISA analyses, respectively. The experiment was performed in technical triplicate. (**d**) MCT2-depleted MDA-MB-231 and MCT2-overexpressing MDA-MB-468 cells were treated with various doses of β-hydroxybutyrate in soft agar colony formation assays. (**e**) Tumour growth assays in NOD/SCID/γ^null^ mice. MCT2-depleted MDA-MB-231 and MDA-MB-157 cells were subcutaneously injected in both flank of each mouse, and the mice were administered PBS alone or PBS containing β-hydroxybutyrate (500 mg kg^−1^) through daily intraperitoneal injection. The tumour volumes were measured every 7 days. Six mice (*n*=6) were used for each group. The experiments in **a**,**b**,**d** were performed in technical triplicate and repeated at least twice with similar results. In **a**–**e**, data show means±s.d. *P<0.05 (Student's *t*-test).

Based on the design of the *in vitro* co-culture system, the effects from MGDAs are most likely mediated by secreted soluble factors. Although adipocytes secrete many adipokines^9^, a systematic analysis of factors from MGDAs is necessary. We collected and fractioned MGDAs-conditioned medium (CM) based on molecular weight into <10 kD, 10–50 kD and >50 kD fractions via selected filters and centrifugation. Only the <10 kD fraction stimulated colony formation when compared to cell culture medium (not MGDAs-CM) in the soft agar colony formation assay using MDA-MB-231 and MCF7 cells (Fig. 4a and Supplementary Fig. 3A). However, treatment with the same fraction did not enhance colony formation of MDA-MB-468, a non-responsive cell line. Furthermore, depletion or ectopic expression of MCT2 in MDA-MB-231, MCF7 and MDA-MB-468 cells, respectively, either abrogated or increased the response to the treatment with the <10 kD fraction (Fig. 4b and Supplementary Fig. 3B). These results strongly suggest that the <10 kD fraction of the MGDAs-CM contains at least one diffusible factor that exerts its effect through MCT2.

MCT2 is a monocarboxylate transporter for several monocarboxylic acids, including pyruvate, lactate and β-hydroxybutyrate, which functions to move these molecules from the extracellular space into cells^16^,^17^. Based on the fact that only the <10 kD fraction promoted colony formation in an MCT2-dependent manner, and that MCT2 is mainly for monocarboxylic acid transport, it is likely that one or more of the three small molecules mentioned above is essential for the observed effect of the MGDAs-CM on tumour malignancy.

To evaluate this possibility, we first compared the levels of pyruvate, lactate and β-hydroxybutyrate secreted from MGDAs with the levels from the corresponding stromal vascular fraction (SVF) cells, the second major cell type present in human breast adipose tissue. Although pyruvate, lactate and β-hydroxybutyrate were detected in CM collected from both MGDAs and SVF cells, the levels of β-hydroxybutyrate were significantly higher in the MGDAs-CM than in the corresponding SVF cells (Fig. 4c). To test the potential roles of pyruvate, lactate and β-hydroxybutyrate in promoting tumour malignancy, these small molecules were supplemented in colony formation assays for the MCT2-knockdown MDA-MB-231 and MCT2-overexpressing MDA-MB-468 cells, respectively. Interestingly, we observed that β-hydroxybutyrate (Fig. 4d) and lactate (Supplementary Fig. 3C) enhanced colony formation of MDA-MB-231 cells, but not MCT2-knockdown MDA-MB-231 cells. Supplementing with β-hydroxybutyrate, but not pyruvate or lactate, was also observed to significantly enhance colony formation in MCT2-overexpressing MDA-MB-468 cells (Fig. 4d and Supplementary Fig. 3C). Consistent with previous report that pyruvate was important for supporting proliferation of breast cancer cells^18^, deprivation of pyruvate dramatically abolished the colony formation (Supplementary Fig. 3D), suggesting that pyruvate was also essential for supporting the growth of breast cancer cell in anchorage-independent condition. Since the colony formation of MCT2-expressing breast cancer cells was enhanced when co-culturing with MGDAs in the medium containing 1 mM pyruvate as shown in Figs 1 and 3, simply supplementing with higher amounts of pyruvate did not further promote colony formation (Supplementary Fig. 3C), pyruvate was less likely playing a key role in promoting colony formation of breast cancer cells mediated by MGDAs in an MCT2-dependent manner. Consistently, daily administration of β-hydroxybutyrate via intra-peritoneal (i.p.) injection in mouse xenograft models promoted tumour growth of MDA-MB-231 and MDA-MB-157 cells, but not their MCT2-depleted counterparts (Fig. 4e). To further verify the importance of the role of adipocytes-derived β-HB in breast cancer progression, we designed an experiment using β-HB dehydrogenase (BDH) to remove β-HB in MGDAs-CM. As shown in Supplementary Fig. 4A, β-HB dehydrogenase could covert β-HB into acetoacetate in the presence of cofactor NAD^+^, and the BDH activity could be monitored by the production of NADH at 340 nm (OD_340 nm_). The dynamic range of BDH catalysing enzymatic reaction was shown in Supplementary Fig. 4B. Under this condition, BDH could efficiently convert β-HB up to 8 mM, which was sufficient to convert all the β-HB in the MGDAs-CM (1 ml adipocytes-CM was catalysed with 20 μl BDH (10 U ml^−1^) in the presence of 20 μl NAD^+^ (50 mM) at 37 °C for 1 h). After removal of the β-HB in fractionated adipocytes-CM (<10 kD), the activity to promote the colony number was significantly reduced (Supplementary Fig. 4C). Taken together, these results suggest that β-hydroxybutyrate secreted from adipocytes is responsible for promoting breast cancer progression mediated by MCT2.

### β-hydroxybutyrate alters expression profiles through HDAC

**Figure 5:**
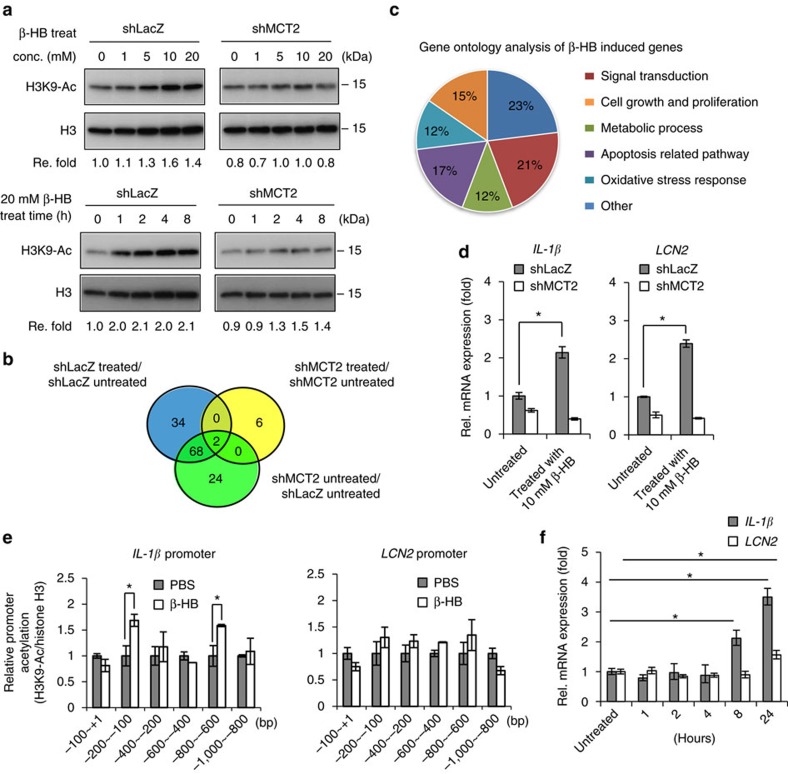
Treatment of β-hydroxybutyrate induces changes of gene expression profiles through epigenetic effects. (**a**) The level of H3K9 acetylation in MCT2-depleted MDA-MB-231 cells was assessed upon β-hydroxybutyrate treatment using a variety of doses and times. The experiment was repeated at least twice with similar results. (**b**) Summary of cDNA microarray analyses. Thirty-four genes were identified with at least 1.5-fold upregulation in β-hydroxybutyrate-treated MDA-MB-231 cells in an MCT2-dependent manner. (**c**) GO analysis of β-hydroxybutyrate-induced genes. The GO terms were classified into six groups and the percentages were shown. (**d**) Q-PCR analyses confirmed the expression levels of *IL-1β* and *LCN2* in MDA-MB-231 cells treated with β-hydroxybutyrate. (**e**) Chromatin immunoprecipitation of acetylated histone H3K9 on *IL-1β* and *LCN2* proximal promoter regions in MDA-MB-231 cells upon 20 mM β-hydroxybutyrate treatment for 1 h. (**f**) *IL-1β* and *LCN2* expression levels in MDA-MB-231 cells treated with 20 mM β-hydroxybutyrate for various times. The experiments in **d**–**f** were performed in technical triplicate and repeated at least twice with similar results. Data show means±s.d. *P<0.05 (Student's *t*-test).

Interestingly, β-hydroxybutyrate was recently recognized as an endogenous inhibitor of class I HDACs (ref. 19). Treatment with β-hydroxybutyrate increases global histone acetylation, such as at histone H3 Lys9 and Lys14 (H3K9 and H3K14), which in turn results in gene activation^19^. To test if β-hydroxybutyrate has a role in altering global histone acetylation of MCT2-expressing breast cancer cells, we treated MDA-MB-231, MCF7 and MDA-MB-157 cells, which all express MCT2, and the corresponding cells depleted with MCT2 with β-hydroxybutyrate. As shown in Fig. 5a and Supplementary Fig. 5A, the level of H3K9 acetylation was enhanced in a dose- and time-dependent manner, while such enhancement was not observed in MCT2-depleted cells. Consistently, when we co-cultured MDA-MB-231 cells with MGDAs, the acetylation of H3K9 was also increased (Supplementary Fig. 5B), arguing that the tumorigenic effect of β-hydroxybutyrate is likely mediated by epigenetic histone modification followed by upregulation of tumour-promoting genes.

To identify which genes were induced by β-hydroxybutyrate in an MCT2-dependent manner, we performed a subtractive microarray analysis as shown in Fig. 5b. About 34 genes in MDA-MB-231 cells treated with β-hydroxybutyrate were found to be upregulated in an MCT2-dependent manner. By performing gene ontology analysis using the Biological Process (BP) GO term, these genes were classified into six groups (Fig. 5c). Intriguingly, most genes on the list were highly expressed in breast cancers compared to normal mammary tissue (data were adapted from Oncomine database, https://www.oncomine.org/resource/login.html) (Table 2). The genes belonging to ‘Cell growth and proliferation' and ‘Apoptosis related pathway' were selected for further validation by Q-PCR analysis. Among these genes, *IL-1β* and *LCN2* were increased the most upon β-hydroxybutyrate treatment (Fig. 5d). Furthermore, the expressions of *IL-1β* and *LCN2* were also induced in breast cancer cells co-culture with MGDAs (Supplementary Fig. 5C). To test whether the upregulation of *IL-1β* and *LCN2* is attributed to histone H3K9 acetylation, we performed chromatin immunoprecipitation (ChIP) analysis of the *IL-1β* and *LCN2* promoters with six primer pairs of primers spanning each promoter region. It was noted that histone H3K9 acetylation of the *IL-1β* was immediately increased after β-hydroxybutyrate treatment for 1 h, but not *LCN2* (Fig. 5e). Consistently, the upregulation of *LCN2* occurred later than *IL-1β* (Fig. 5f), suggesting that the induction of *LCN2* expression may not be a direct response to β-hydroxybutyrate treatment. Taken together, these results suggest that promotion of breast tumorigenicity by β-hydroxybutyrate is likely resulted from upregulation of tumour-promoting genes through epigenetic modification of chromatin.

### IL-1β and LCN2 contribute to MGDAs-mediated malignancy

**Figure 6:**
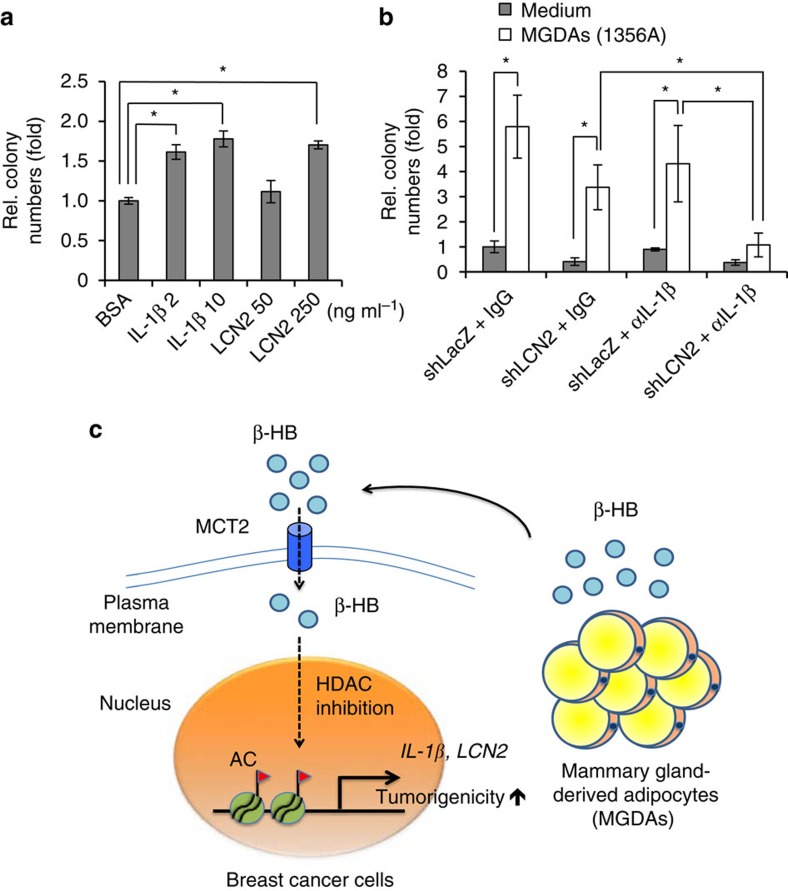
IL-1β and LCN2 play important roles in MGDAs-mediated tumorigenicity enhancement. (**a**) MDA-MB-231 cells were supplemented with recombinant IL-1β and LCN2 in soft agar colony formation assays. (**b**). Soft agar colony formation assays showed that knockdown of LCN2 combining IL-1β neutralizing antibody treatment (αIL-1β, 1 μg ml^−1^) significantly abrogated the increase of colonies induced by MGDAs co-culture with MDA-MB-231 cells. Mouse IgG (1 μg ml^−1^) was used as a control. The experiments in **a**,**b** were performed in technical triplicate and repeated at least twice with similar results. Data show means±s.d. *P<0.05 (Student's *t*-test). (**c**) Schematic shows the heterotypic interaction between MGDAs and MCT2-expressing breast cancer cells in the breast tissue microenvironment. MGDAs promote the tumorigenicity of MCT2-expressing breast cancer cells in a paracrine manner. β-hydroxybutyrate secreted from MGDAs can be transported into breast cancer cells via MCT2. Intracellular β-hydroxybutyrate functions as a class I HDAC inhibitor and induces the expression of tumour-promoting genes through epigenetic modification, leading to tumorigenicity enhancement.

The roles of IL-1β and LCN2 in breast cancer progression have been reported^20^,^21^. To examine their roles on promoting colony formation mediated by MGDAs, we added recombinant IL-1β and LCN2 proteins in soft agar colony formation assays, and found that either of them increased colony numbers in a dose-dependent manner (Fig. 6a). On the other hand, knockdown of either *IL-1β* or *LCN2* in MDA-MB-231 only slightly abrogated MGDAs-mediated colony promotion (Supplementary Fig. 6A). Although depletion of both *IL-1β* and *LCN2* abolished the MDGAs-mediated colony promotion of MDA-MB-231 breast cancer cells, it also caused severe inhibition of cell growth (Supplementary Fig. 6B). To avoid the severely retarded growth effect caused by the double knockdown, we used IL-1β-neutralizing antibody to treat LCN2-depleted MDA-MB-231 breast cancer cells for evaluating the colony formation. As shown in Fig. 6b, adding IL-1β antibody further reduced the colony number of LCN2-depleted MDA-MB-231 breast cancer cells co-cultured with MGDAs, suggesting that both IL-1β and LCN2 contribute to MGDAs-mediated tumour malignancy. Based on the above results, a novel molecular mechanism linking adipocytes and breast cancer progression via MCT2 was established. The heterotypic cell-cell interaction within the breast cancer microenvironment upregulated IL-1β/LCN2 expressions in breast cancer cells, which in turn markedly enhanced breast cancer tumour progression (Fig. 6c).

### MCT2 and IL-1b/LCN2 expressions link to poor prognosis

**Figure 7:**
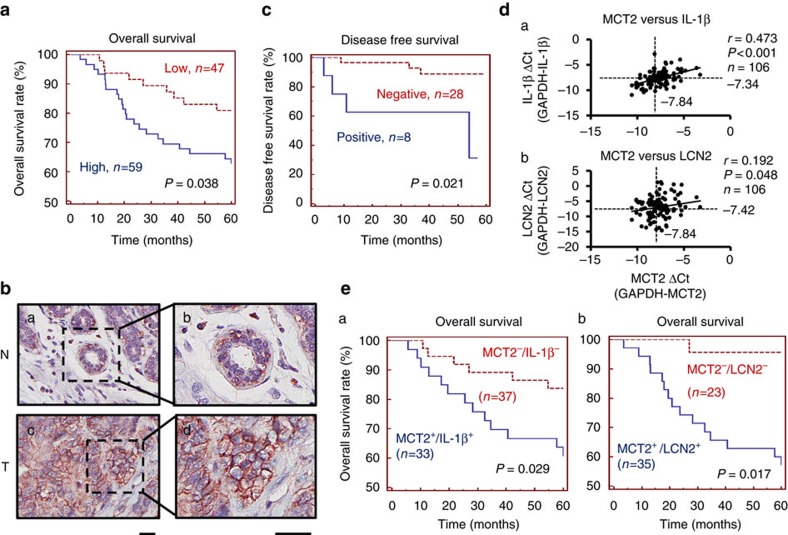
Elevated expressions of *IL-1β* and *LCN2* correlate with poor prognosis in breast cancer patients. (**a**) Kaplan-Meier analysis showed correlation between cumulative survival and *MCT2* expression levels in breast cancer patients. (**b**) IHC staining of MCT2. The pictures showed the negative (**a**,**b**) and positive (**c**,**d**) membrane staining of MCT2 in normal and breast cancer tissues, respectively. (Scale bars, 25 μm). (**c**) Kaplan-Meier survival analysis of patients with MCT2-positive and -negative IHC staining. (**d**) Correlation analyses of gene expression levels between *MCT2*, *IL-1β* and *LCN2*. *MCT2* versus: (**a**) *IL-1β* and (**b**) *LCN2*. (**e**) Survival analyses showed (a) *MCT2* and *IL-1β* (b) *MCT2* and *LCN2* double-positive group versus double-negative group, respectively.

To further affirm the significance of MCT2 in breast cancer malignancy, RNA samples from a cohort of 106 breast cancer specimens were analysed using Q-PCR. Threshold (−ΔCt=−7.84) was used as a cut-off value based on a receiver operating characteristic curve analysis to define ‘high or low' expression of *MCT2*. Kaplan-Meier (KM) analysis showed that patients with high *MCT2* expression had a shorter survival time compared to patients with low *MCT2* expression, *n*=106 (Fig. 7a, *P*=0.038, log-rank test). The association of *MCT2* expression with poor prognosis was also significant even after adjustment for age, tumour size, lymph node status, grade and ER expression (Table 3). These results suggest that MCT2 is an independent prognostic factor in patients with breast cancer. To validate this result, another independent breast cancer cohort with larger sample size (*n*=327, Supplementary Tables 3 and 4, data adapted from the breast tumour gene expression profiling by Kao *et al*.^22^ was used for further survival analysis. As shown in Supplementary Fig. 7A, KM analysis showed that patients with high *MCT2* expression had a shorter survival time compared to patients with low *MCT2* expression (*P*=0.003, log-rank test). Furthermore, expression of *MCT2* in breast ductal carcinoma was increased when compared to normal mammary tissues (Supplementary Fig. 7B, data adapted from the breast tumour gene expression profiling by Richardson *et al*.^23^

In addition, we have validated the specificity of MCT2 antibody in immunohistochemical staining as shown in Supplementary Fig. 8. We then performed examination of MCT2 expression in another independent cohort of 36 breast cancer specimens. Breast cancer patients with high MCT2 expression correlated with poor prognosis (Fig. 7b,c, 22% of the cases were MCT2 positive, *P*=0.021, log-rank test). Furthermore, the expressions of *IL-1β* and *LCN2* mRNA were positively correlated with *MCT2* expression in breast cancer specimens (Fig. 7d). The correlation between *MCT2* expression and poor prognosis in breast cancer was further strengthened when combined with either high expression of *IL-1β* or *LCN2* (cut-off value of −ΔCt: −7.34 for IL-1β and −7.42 for LCN2, respectively) (Fig. 7e). These findings suggest that high expression of *MCT2*, in combination with *IL-1β* or *LCN2* expression, may serve as an important clinical biomarker for poor prognosis in breast cancer patients.

## Discussion

It has been shown that larger breast size is associated with an increased risk of breast cancer progression. This correlation is even more significant in the study of decreased cancer risk by breast reduction^24^. Recent work further demonstrates that breast size is highly correlated with breast cancer mortality even after adjustment for BMI (ref. 25), suggesting that local adiposity in breast tissue may have a significant impact on cancer progression. Here, we find that the monocarboxylate transporter protein MCT2, expressed in a distinct subtype of breast cancer cells, plays a pivotal role in the interplay with MGDAs. We show that MGDAs promotion of malignant growth in MCT2-expressing breast cancer cells requires β-hydroxybutyrate. Consistently, treatment of MCT2-expressing breast cancer cells with either β-hydroxybutyrate or co-culture with MGDAs enhances tumorigenic properties and leads to increased acetylation of histones and transcriptional upregulation of tumour-promoting genes such as *IL-1β* and *LCN2* in breast cancer cells expressing MCT2. As IL-1β and LCN2 appear to promote breast tumour malignancy and lead to a poorer prognosis, we speculate that β-hydroxybutyrate induced upregulation of their expression might contribute to the observed increased tumorigenesis. This mode of crosstalk between breast tissue adipocytes (MGDAs) and breast cancer cells may have potential clinical ramifications.

Adipocytes secrete a battery of factors including growth factors and adipokines^9^. IL-6 was identified as an important factor involved in promoting tumour cell invasion^12^ and proliferation of ERα-positive breast cancer cells^26^. Interestingly, the IL-6 receptor was found to be upregulated in MGDAs-responding cell lines in our systematic screening reported here, suggesting that the screening strategy by subtraction of gene expression profiles was a rational design (Table 1). However, depletion of IL-6R did not completely abrogate the malignancy promotion conferred by MGDAs, indicating that an unknown pathway distinct from the IL-6R/IL-6 signalling mediates the promotion of tumour malignancy by adipocytes.

In addition to adipokines, an elevated level of oestrogen was thought to be the major factor for ERα-positive breast cancer risk in postmenopausal women^27^. When ovarian production of female steroid hormones decreases, adipose tissue becomes an important source for oestrogen production due to the expression of aromatase, a rate-limiting enzyme for estradiol synthesis^28^. Since MGDAs promoted tumour malignancy regardless of the ERα status of the tested cells (Fig. 1), the oestrogen level does not appear to be the determining factor. Interestingly, adipocytes secrete β-hydroxybutyrate, which promotes tumour malignancy in an MCT2-dependent manner (Figs 4 and 5). While it could not be directly determined *in vivo* that breast adipocytes secrete β-hydroxybutyrate specifically to promote tumour malignancy, our results that depletion of β-hydroxybutyrate present in primary MGDAs diminished MGDAs-mediated promotion of tumorigenic activity *in vitro* (Supplementary Fig. 4) suggest that β-hydroxybutyrate either from breast adipocytes or other source promotes tumorigenic activity.

It was reported that elevated expression of monocarboxylate transporters (MCTs) may have relevance to tumour progression^29^. MCT1 was observed in several cancers, such as breast and colorectal cancers^30^,^31^, while MCT2 and MCT4 were highly expressed in prostate cancer and renal cell carcinoma, respectively^32^,^33^. Intriguingly, MCT2, but not MCT1, played an important role in MGDAs-mediated promotion of tumorigenic activity as observed above. Several lines of evidence supported this notion; co-culturing with MGDAs and treatment of β-hydroxybutyrate shown in Figs 1 and 3 failed to promote the colony numbers of MDA-MB-468 cells, which expressed relatively higher level of MCT1 (Supplementary Fig. 9A). Furthermore, ectopic expression of MCT1 failed to rescue MCT2-depleted MDA-MB-231 cells in response to β-hydroxybutyrate treatment (Supplementary Fig. 9B,C). MCT2 showed higher affinity (about tenfold increase) to most monocarboxylates than MCT1 (ref. 34). Although the biological difference between MCT1 and MCT2 in response to β-hydroxybutyrate is evident, the detailed mechanism to explain this difference deserves further exploration.

MCT2 has been reported to locate in mitochondria with a role for mitochondrial metabolism^35^, and inhibition of MCT2 suppresses colorectal cancer progression via induction of mitochondrial dysfunction^36^. However, our results revealed that co-culture of MGDAs with breast cancer cells increased tumour malignancy in a paracrine and MCT2-dependent manner, suggesting that the major role of MCT2 is likely for the function across cell membrane of cancer cells. Through immunofluorescence staining analysis, a membrane-staining pattern of MCT2 in breast cancer cells was revealed (Supplementary Fig. 10). Consistently, membrane staining of MCT2 was correlated with poor prognosis in breast cancer patients (Fig. 7b,c).

Increase of the circulating level of β-hydroxybutyrate through supplementation or ketogenic diet was reported to prolong survival of mice with brain metastatic tumour^37^,^38^. At the same setting, the authors also found that decreased blood glucose was also correlated with longer survival in brain tumour bearing mice. Thus, it was not clear whether decrease of blood glucose or increase of β-hydroxybutyrate contributed to tumour reduction. Several experiments showed that ketogenic diet without calories restriction and blood glucose decrease did not reduce tumour growth^39^,^40^,^41^. These results suggest that reduction of blood glucose may be the key factor to restrict tumour progression *in vivo* despite the presence of high level of circulating ketone bodies. We have observed here that addition of β-hydroxybutyrate promoted breast tumour growth in an MCT2-dependent manner in a mouse xenograft model. In those mice fed with regular chow diet *ad libitum*, the blood glucose level was not changed (Supplementary Fig. 11). This finding reflects a delicate role of the metabolite balance in tumour progression. Although ketone supplement reportedly decreased brain tumour cell viability in *vitro*^38^, treatment of β-hydroxybutyrate did not reduce proliferation in breast cancer cells (Supplementary Fig. 12), suggesting that brain and breast cancer cells have different response to β-hydroxybutyrate. Unlike brain and muscle cells^42^, breast cancer cells could not use β-hydroxybutyrate as alternative energy source (Supplementary Fig. 13). Thus, β-hydroxybutyrate may serve as HDAC inhibitor, as reported recently^19^, to modulate gene expression in breast cancer cells.

Treatment of HDAC inhibitors globally increases the acetylation of histones H3 and H4 (ref. 43), which is generally associated with transcription activation and gene upregulation^44^. Although the HDAC inhibitors are considered as promising agents for certain haematological tumours treatment, the efficacy in treating solid tumours is limited^45^. It is likely that the accessibility of the HDAC inhibitors to these two kinds of malignancy may explain the discrepancy. In fact, treatment of low and non-cytotoxic dosages of HDAC inhibitors preferentially induces epithelial-to-mesenchymal transition and proliferation genes expression^46^,^47^,^48^. Our observation that treatment of cancer cells expressing MCT2 with β-hydroxybutyrate, a weak HDAC inhibitor, increased acetylation of histones, and upregulated expressions of *IL-1β* and *LCN2* (Figs 5 and 6), is consistent with low dose effect of HDAC inhibitor as reported^46^,^47^,^48^. Furthermore, the significant correlation between *MCT2* and *IL-1β*/*LCN2* expressions in clinical breast cancer specimens supports the role of an MCT2-mediated oncogenic pathway in breast cancer progression. Thus, our findings provide the mechanistic basis for a novel interaction between breast tissue adipocytes and breast tumour. Further research into this detailed mechanism may offer new insights for better diagnostic and prognostic assessments, and for new therapeutic opportunities.

## Methods

### Ethics statement

Human samples were obtained from National Taiwan University Hospital (NTUH) and Wan Fang Hospital, Taipei Medical University. The samples were encoded to protect patient confidentiality. All patients signed an informed consent, which was approved by the Institutional Review Board of Human Subjects Research Ethics Committee of National Taiwan University, Taipei, Taiwan (IRB no. 200902001R) and Wan Fang Hospital, Taipei Medical University (IRB no. WFH-IRB-99049). Clinical information was obtained from pathology reports, and the characteristics of these cases are given in Supplementary Tables 1 and 2. Patients with at least 5 years follow-up were included in this study. The animal studies were approved by the Institutional Animal Care and Use Committee of the Academia Sinica, Taipei, Taiwan (Protocol # 14-05-708). The experiments were performed in technical triplicate and repeated at least twice independently. Similar results were observed and one representative result was shown.

### Cell culture

Human breast cancer cell lines MCF7, MDA-MB-157, MDA-MB-231, MDA-MB-361, MDA-MB-468 and SKBR3 were obtained from American Type Culture Collection and cultured in Dulbecco's modified Eagle's medium (DMEM) supplemented with 10% fetal bovine serum (FBS), 2 mM L-glutamine, 1 mM non-essential amino acids, 1 mM sodium pyruvate and antibiotics/antimycotics in a humidified 37 °C incubator supplemented with 5% CO_2_. The cell lines were regularly checked for mycoplasma infections.

### Statistical methods

Except for the clinical correlation and quantification for specific immunoblots, all data were presented as means±s.d., and Student's *t*-test (two-tailed, unpaired) was used to compare control and treatment groups. *indicated statistical significance with *P*<0.05. Data distribution was assumed to be normal. The variance was similar between the groups that were being statistically compared (by *F*-test). We chose our sample sizes based on those commonly used in this field without predetermination by statistical methods. The following analyses were performed using MedCalc statistics software. KM method was plotted and log-rank test was used to evaluate the statistical significance between patients with high and low MCT2 expression in survival. To determine the effects of different variables on overall survival, univariate Cox proportional-hazards regression analysis was performed. Multivariate Cox regression analysis was used to adjust the association between survival and MCT2 gene expression level for varying clinical parameters including age, tumour size, lymph-node status, tumour grade and ER expression. The optimal cut-off values of MCT2, IL-1β and LCN2 gene expression levels for 5-year survival were determined using receiver operating characteristic analysis. Pearson's correlation was used to evaluate the correlation between MCT2, IL-1β and LCN2 gene expression levels.

### Preparation of primary mammary gland-derived adipocytes

Human breast adipose tissue samples were collected from breast cancer mastectomy specimens in NTUH. Adipose tissue pieces were minced and immediately incubated with digestion buffer (Collagenase I (250 U ml^−1^, Sigma) dissolved in PBS containing 2% bovine serum albumin (Sigma))^14^. For the incubation, the tubes are closed tightly incubated in a 37 °C incubator for 2 h. SVF was separated by centrifugation (5 min, room temperature, 300*g*). Primary MGDAs were cultured in DMEM/F12 medium supplemented with 10% FBS and antibiotics/antimycotics. The purity of the isolated MGDAs was assessed by fluorescence-activated cell sorting analysis using the lipophilic fluorescent dye Nile red at a final concentration of 1 μg ml^−1^ (Sigma, 1 mg ml^−1^ DMSO stock, diluted 1:100 in PBS immediately before use) and nuclear stain DAPI (Sigma) at a final concentration of 1 μg ml^−1^.

### Soft agar colony formation assay

Primary MGDAs (10∼25 μl) mixed with 0.5% agar/complete DMEM/F12 growth medium were added into one well of 12-well plate to form a base layer. After the agar was solidified, 1,500 breast cancer cells suspended in 0.35% agar/1% FBS DMEM medium were seeded on top of bottom agar. Cells were maintained in a humidified 37 °C incubator for 14 days and the colonies were fixed with ethanol containing 0.05% crystal violet for quantification. For supplement experiments, 50 μl serum-free medium containing various dose of β-hydroxybutyrate/lactate/pyruvate (Sigma), human recombinant IL-1β/LCN2 (R&D) and αIL-1β antibody (16–7018, eBioscience) were added every 4 days.

### Xenograft assay in NOD/SCID/γ^null^ mice

For tumorigenicity assay, 10^6^ breast cancer cells mixed with PBS or 50 μl primary MGDAs were mixed with equal volume of Matrigel (BD bioscience), and then co-injected in both flanks of each NOD/SCID/γ^null^ mouse (4∼5-week-old, female) subcutaneously. Tumour volumes were evaluated every 4 days after initial detection. Student's *t*-test was used to test the significant differences. We chose our sample sizes based on those commonly used in this field without predetermination by statistical methods. The age- and weight-matched mice were randomly divided into each experimental group. The systemic administration of β-hydroxybutyrate (500 mg kg^−1^ per mouse) was performed by daily i.p. injection, and PBS was injected as control. The investigators were not blinded to the group allocation during experiments and outcome assessment.

### Immunoblotting

Whole-cell extracts were prepared by scraping in RIPA lysis buffer (50 mM Tris-HCl (pH, 7.4), 150 mM NaCl, 1% NP-40, 1% Triton X-100, 2 mM EDTA, 50 mM NaF, 0.5% sodium deoxycholate, 0.1% SDS, 1 mM PMSF and 1 × protease inhibitor cocktail (cOmplete, Roche)) followed by centrifugation at 13,000 r.p.m. at 4 °C. Immunoblot analysis was performed after 10 or 15% SDS-PAGE, with overnight incubation of 1:200 dilution of anti-MCT2 (sc-50322, Santa Cruz), or 1:1,000 dilution of anti-ENPP1 (GTX103447, GeneTex), anti-acetyl-Histone H3 (Lys9) (#9649, Cell Signaling Technology), anti-Histone H3 (#4499, Cell Signaling Technology), anti-MCT1 (AB3538P, Merck Millipore) antibodies followed by a 1:10,000 dilution of horseradish peroxidase-conjugated anti-mouse or anti-rabbit secondary antibody (GeneTex). Signals were detected using Immobilon Western Chemiluminescent HRP Substrate (Merck Millipore). Protein concentration was determined by the Bradford assay (Bio-Rad) before loading and verified by α-tubulin level using a 1:10,000 dilution of anti-α-tubulin antibody (GTX72360, GeneTex). The intensity of western blot bands was quantified using ImageJ software (NIH). The uncropped scans of immuno-blots can be found in Supplementary Figs 14 and 15.

### Immunohistochemistry

Formalin-fixed paraffin embedded primary tumour tissue sections were stained by using the Ventana automated immunostainer BenchMark LT (Ventana Medical Systems). Homemade mouse anti-MCT2 monoclonal antibody (1 μg μl^−1^) was diluted to 1:200, and incubated at room temperature for 2 h. Signals were detected using the OptiView DAB IHC Detection Kit (Ventana Medical Systems). All slides were counterstained with haematoxylin, and the images were taken using an Aperio Digital Pathology System. Samples were identified as MCT2-positive if more than 5% of the tumour cells were positive for membrane staining.

### Co-culture and conditioned medium collection

CM from MGDAs or SVF cells were collected using a Transwell culture system (0.4-mm pore size; Merck Millipore). MGDAs (50–100 μl) or 10^5^ SVF cells were seeded in the top or bottom chambers of the Transwell system (12-well format) in the culture medium of MGDAs/SVF cells. CM was collected by overnight cultivation in serum-free medium. The fractionated CM was collected using ultrafiltration with different molecular weight cut-off filters (Amicon, Merck Millipore). For co-culture of MGDAs and breast cancer cells, MGDAs and breast cancer cells were seeded into the top and bottom chambers of the Transwell system (12-well format) in the culture medium of MGDAs and breast cancer cells, respectively.

### Plasmids and shRNA

The cDNA of *ARMCX1*, *ENPP1* and *MCT2* were amplified from MDA-MB-231 cells and cloned into pLVX-IRES-Neo mammalian expression vector (Clontech) using restriction enzymes and primers listed in Supplementary Table 5. The lentiviral shRNA expression vectors of pLKO.1-shLacZ, shABCG2, shAREG, shARMCX1, shATP2B1, shENPP1, shFRAS1, shFRMD5, shGPC6, shGPR126, shIL6R, shOSBPL8, shRAB8B, shRFTN1, shRHOF, shMCT2, shTHBD, shIL-1β and shLCN2 were purchased from the National RNAi Core Facility (Taipei, Taiwan). The target sequences of shRNA were listed in Supplementary Table 6. For lentivirus production, HEK-293T cells were co-transfected with shRNA containing the lentiviral vector, envelope plasmid pMD.G and packaging plasmid pCMVΔR8.91. Virus-containing supernatants were collected at 24 and 48 h post-transfection. MDA-MB-157, MDA-MB-231 and MCF7 cells were infected with lentivirus and then selected with 2 μg ml^−1^ puromycin.

### RNA extraction and cDNA preparation for Q-PCR and microarray

Total RNA from cell culture and tumour tissue was isolated using TRIzol reagent (Invitrogen) and reverse-transcribed with Transcriptor First Strand cDNA Synthesis kit (Roche) for gene expression analysis according to instructions from the manufacturers. Quantitative real-time RT-PCR was performed using KAPA SYBR FAST qPCR kit (KAPA Biosystems) for gene expression according to the manufacturer's instructions and analysed on a StepOnePlus Real-Time PCR system (Applied Biosystems). GAPDH mRNA was used as an internal control for mRNA expression. Expression levels were calculated according to the relative ΔCt method, and the specificity of each primer pair was determined by dissociation curve analysis. All primers were listed in Supplementary Table 7. Phalanx Human OneArray Ver. 6 Release 1 (Phalanx Biotech Group) was used to detect gene expression.

### Measurements of metabolites in CM

The measurements of β-hydroxybutyrate, lactate and pyruvate levels in CM were performed using ELISA colorimetric assay kits (BioVision) according to instructions from the manufacturer. The levels of β-hydroxybutyrate, lactate and pyruvate were normalized with protein concentration.

### ChIP assay

MDA-MB-231 cells were starved by 1% FBS medium overnight and treated with 20 mM β-hydroxybutyrate for 1 h. Two 10-cm plates cells per antibody were treated with 1% formaldehyde for 10 min at room temperature. Cross-linking reaction was stopped by the addition of 0.125 M glycine (final concentration). Cells were collected in cold PBS containing 1 mM PMSF. Cell pellet was resuspended in ChIP Swelling buffer (5 mM PIPES (pH, 8.0), 85 mM KCl, 1% NP-40, and 1 mM PMSF) and incubated at 4 °C for 20 min. Then the nuclei pellet collected by centrifugation was resuspended in Nuclei Lysis buffer (50 mM Tris-HCl (pH, 8.0), 10 mM EDTA, 1% SDS and 1 mM PMSF) and sonicated to an average size of 500 bp. After sonication and centrifugation at 13,000 r.p.m. for 15 min at 4 °C, the supernatant was added ChIP dilution buffer (16.7 mM Tris-HCl (pH, 8.0), 167 mM NaCl, 1.2 mM EDTA, 0.01% SDS, 1.1% Triton X-100 and 1 mM PMSF). IPs were performed with anti-acetyl-Histone H3 (Lys9) (#9649, Cell Signaling Technology), anti-Histone H3 (ab1791, Abcam) and rabbit IgG control (sc-2027, Santa Cruz Biotechnology) antibodies and incubated at 4 °C overnight. The beads were washed twice and eluted by 1% SDS elution buffer (1% SDS, 0.1 M NaHCO_3_, and 0.01 mg ml^−1^ herring sperm DNA). After treatment with Proteinase K, DNA was extracted with phenol-chloroform, precipitated with ethanol and dissolved in Tris-EDTA buffer (10 mM Tris-HCl (pH, 8.0) and 1 mM EDTA). ChIP products and input DNA were analysed by Q-PCR. Primers spanning the promoter regions of the human IL-1β and LCN2 genes are shown in Supplementary Table 8.

### Immunofluorescence staining

MDA-MB-231 and MDA-MB-468 cells were seeded on coverslips for immunofluorescence staining. The cells were washed with PBS, fixed in 4% paraformaldehyde for 10 min and permeabilized in 0.2% Triton X-100 for 15 min at room temperature. After washing with PBS, the cells were blocked with PBS containing 10% FBS for 30∼60 min, and incubated with homemade mouse anti-MCT2 monoclonal antibody (1:2,000) overnight at 4 °C. The cells were washed with PBS and incubated with fluorochrome-conjugated secondary antibody (1:500, Alexa Fluor 488 goat-α-mouse, Invitrogen) for 1 h. The coverslips with stained cells were then washed, stained with DAPI (1:1,000) and mounted onto glass slides with mounting medium and examined by Leica TCS-SP5-MP-SMD confocal microscope.

### Mitochondrial respiration analysis

The mitochondrial bioenergetics profiles in breast cancer cells were performed using a Seahorse XF96 Cell Mito Stress Test Kit (Seahorse Biosciences) according to instructions from the manufacturers. MDA-MB-231 cells were seeded at 20,000 cells per well (96-well Seahorse cell culture plate) in pyruvate-free condition and incubated overnight. On the day of assay, the culture medium was replaced with 175 μl of Seahorse assay medium supplemented with different concentrations of pyruvate or β-hydroxybutyrate and then incubated for 1 h at 37 °C without CO_2_. The oxygen consumption rate (OCR) was determined by Seahorse XFe96 analyser (Seahorse Biosciences). During the period of OCR measurement, sequential injection of 1 μM oligomycin, 0.5 μM carbonyl cyanide p-trifluoromethoxyphenyl hydrazone (FCCP) and 0.5 μM rotenone/antimycin were used to determine the mitochondrial function parameters including basal OCR, maximal OCR and reserve respiratory capacity.

### Data availability

Gene expression microarray data of β-hydroxybutyrate treatment were deposited in the Gene Expression Omnibus (GEO) database with an accession number of GSE84053. The Oncomine data referenced during the study are available in a public repository from the website (https://www.oncomine.org/resource/login.html). The authors declare that all the other data supporting the findings of this study are available within the article and its Supplementary Information files and from the corresponding author on reasonable request.

## Additional information

**How to cite this article:** Huang, C.-K. *et al*. Adipocytes promote malignant growth of breast tumours with monocarboxylate transporter 2 expression via β-hydroxybutyrate. *Nat. Commun.* **8,** 14706 doi: 10.1038/ncomms14706 (2017).

**Publisher's note:** Springer Nature remains neutral with regard to jurisdictional claims in published maps and institutional affiliations.

## Supplementary Material

Supplementary Information Supplementary Figures and Supplementary Tables

Supplementary Data 1 Gene expression microarray data for screening potential factors involved in MDGAs-mediated tumorigenic enhancement.

Peer Review File

## Figures and Tables

**Table 1:** List of membrane or membrane-associated candidate genes.

**Gene name**	**MB361/MB468**	**MB231/MB468**	**MB157/MB468**
	**Log**_**2**_ **ratio**		
*ABCG2*	2.3	4.4	6.3
*AREG*	2.0	4.4	4.0
*ARMCX1*	6.8	6.6	6.9
*ATP2B1*	2.6	2.9	2.3
*ENPP1*	4.0	2.8	4.6
*FRAS1*	2.9	2.7	2.6
*FRMD5*	4.4	4.0	1.7
*GPC6*	4.5	5.4	3.2
*GPR126*	1.6	1.8	1.8
*IL6R*	1.0	2.4	2.4
*OSBPL8*	1.8	2.2	1.9
*RAB8B*	3.4	3.4	1.4
*RFTN1*	5.8	5.7	2.8
*RHOF*	3.2	3.2	2.0
*MCT2*	5.1	5.3	6.2
*THBD*	5.0	4.6	4.1

**Table 2:** List of β-hydroxybutyrate-induced genes for each GO term.

**Term**	**Genes (ranked by induction fold)**
Signalling transduction	IL-1B*, IL6*, LCN2*, ISG20*, PIK3CD*, TNIP2*, CXCL1, IL11*, WNT5B*, ANKRD1*, EIF2C4*
Cell growth and proliferation	IL-1B*, SOD2, IL6*, ISG20*, LEPREL2*, GATA2*, CXCL1, IL11*
Metabolic process	IL-1B*, ADA*, ISG20*, KYNU*, ANKRD1*, TUBB2B
Apoptosis-related pathway	IL-1B*, OLR1*, SOD2, IL6*, ADA*, LCN2*, TNIP2*, TREX1*, ANKRD1*
Oxidative stress response	IL-1B*, OLR1*, SOD2, IL6*, ADA*, LCN2*

Asterisks indicated that genes were highly expressed in breast tumour compared to normal mammary tissue (data were adapted from Oncomine database, https://www.oncomine.org/resource/login.html).

**Table 3:** Univariate and multivariate proportional hazards analyses of mortality in breast cancer patients according to *MCT2* expression levels.

**Variables**	**Univariate HR (95% CI)**	****P** value**	**Multivariate HR (95% CI)**	***P* value**
MCT2 high-risk group (versus low-risk group)	2.22 (1.03–4.82)	**0.043**	3.59 (1.28–10.08)	**0.016**
Age (≥48 years old)*	0.76 (0.37–1.53)	0.440	0.47 (0.19–1.17	0.107
Tumour size (>2 cm)	4.83 (1.16–20.09)	**0.031**	4.00 (0.49–32.70)	0.199
Lymph-node status (per grade)†	2.36 (1.38–4.04)	**0.002**	3.87 (1.77–8.45)	**0.001**
Grade (per grade)	0.60 (0.31–1.16)	0.134	1.09 (0.51–2.34)	0.823
Estrogen-receptor positive stain (versus negative group)	0.40 (0.19–0.81)	**0.012**	0.10 (0.04–0.29)	**<0.0001**

CI, confidence interval; HR, hazard ratio. Bold entries indicate *P* value <0.05.

^*^Median of age: 48 years old.

^†^Lymph-node positive, 0:0, 1:1∼3, 2: >4.
