# Supplementary material for: Adipocytes promote malignant growth of breast tumours with monocarboxylate transporter 2 expression via β-hydroxybutyrate
Source: Nat Commun. 2017 Mar 10;8:14706. doi: 10.1038/ncomms14706 (PMC5353665; doi:10.1038/ncomms14706)
Supplement: Supplementary Information — Supplementary Figures and Supplementary Tables [file ncomms14706-s1.pdf]

Supplementary information:

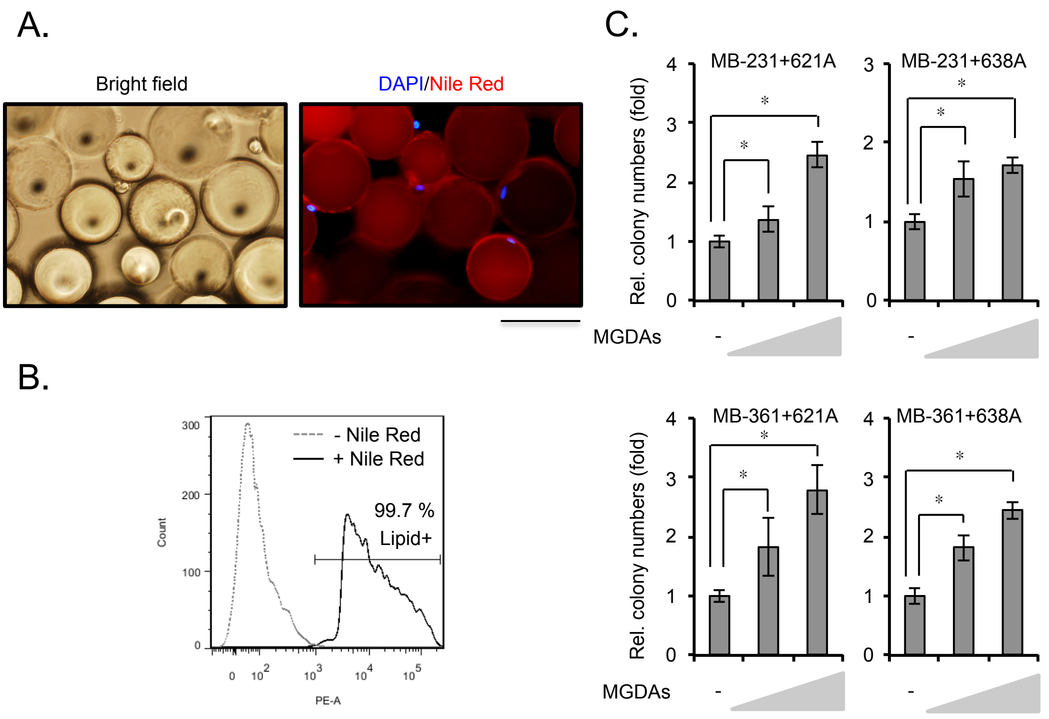

Supplementary Figure 1. Co-culturing with primary MGDAs increases the number of breast cancer cell colonies

(A) Representative images of primary MGDAs isolated from breast cancer clinical specimens. The MGDAs were double-stained with Nile red and DAPI nuclear stain (Bar, 100 μm). (B) The purity of isolated MDGAs was characterized by Nile red/DAPI double staining and analyzed by FACS. (C) Soft agar colony formation assays using MDA-MB-231 and MDA-MB-361 breast cancer cells co-cultured with two different MGDAs. The experiment in C was performed in technical triplicate and repeated at least twice with similar results. Data show means ± s.d. *, $p < 0.05$ (Student's t-test).

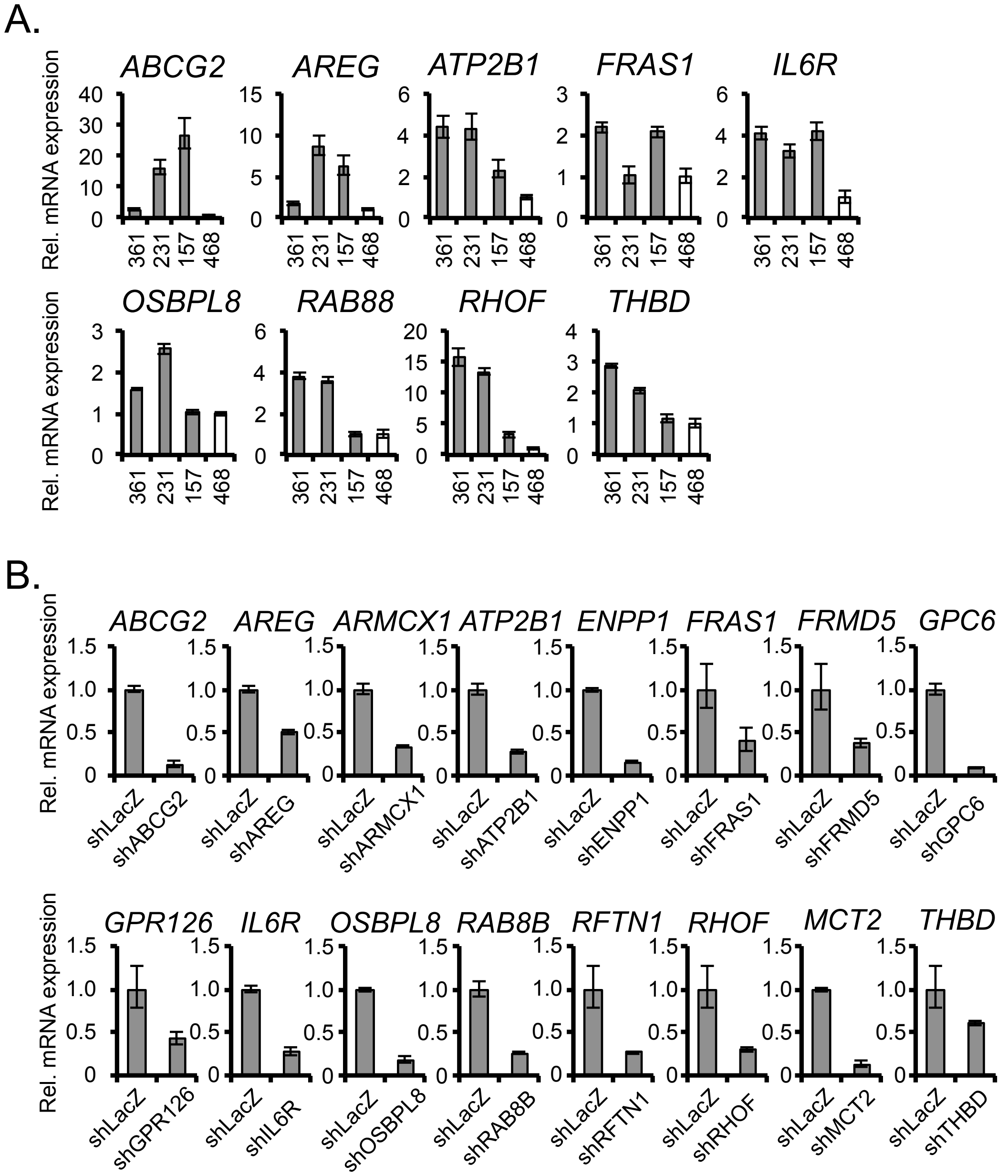

Supplementary Figure 2. Q-PCR analyses confirm the expression levels and knockdown efficiencies of selected candidate genes from the microarray data subtractions

(A) Expression level of each candidate gene in MDA-MB-157, MDA-MB-231, and MDA-MB-361 relative to MDA-MB-468 is indicated, see also Figure 2C. (B) RNAi knockdown efficiency for each candidate gene in MDA-MB-157 breast cancer cells was confirmed by Q-PCR analyses. The experiments in A and B were performed in technical triplicate. Data show means ± s.d.

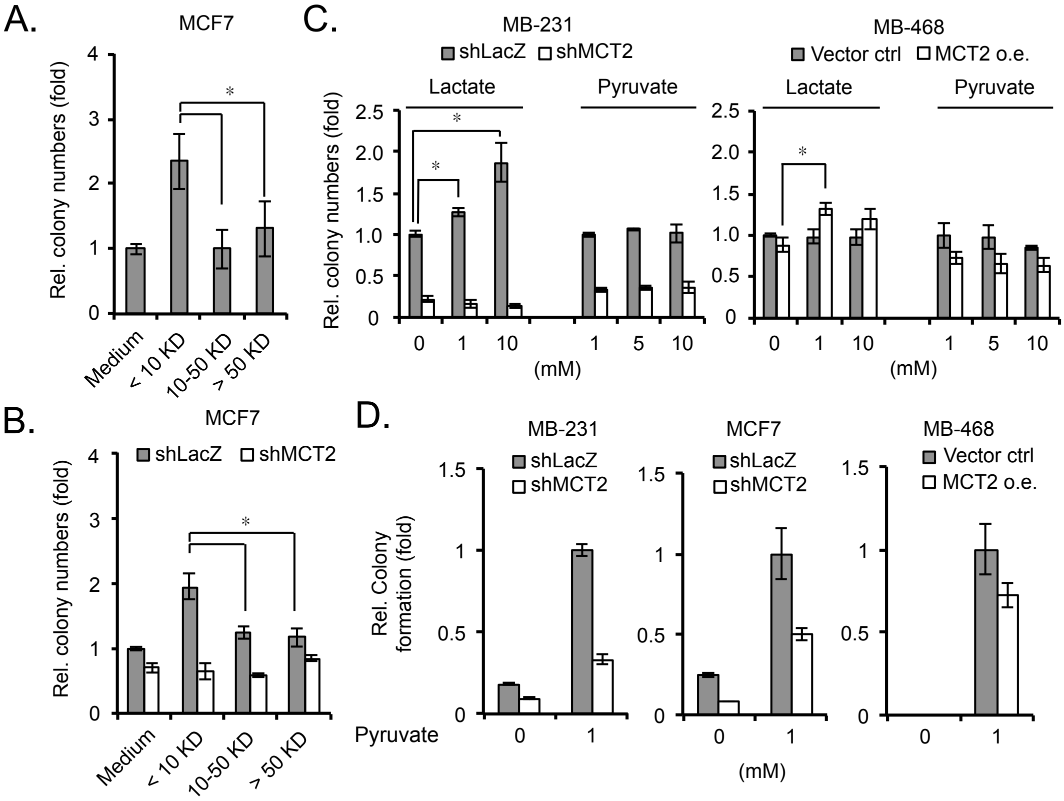

Supplementary Figure 3. Lactate and pyruvate show partial or no effect on colony formation in MCT2-expressing breast cancer cells

(A) Soft agar colony formation assay of MCF7 cells treated with fractionated MGDAs-conditioned medium. (B) Soft agar colony formation assay of MCT2-depleted MCF7 cells treated with fractionated MGDAs-conditioned medium. (C) MCT2-depleted MDA-MB-231 and overexpressing MDA-MB-468 cells were treated with various doses of lactate and pyruvate in soft agar colony formation assays. (D) Colony-forming ability was dramatically reduced when pyruvate was completely removed from the culture medium. The experiments in A to D were performed in technical triplicate and repeated at least twice with similar results. Data show means ± s.d. *, $p < 0.05$ (Student's t-test).

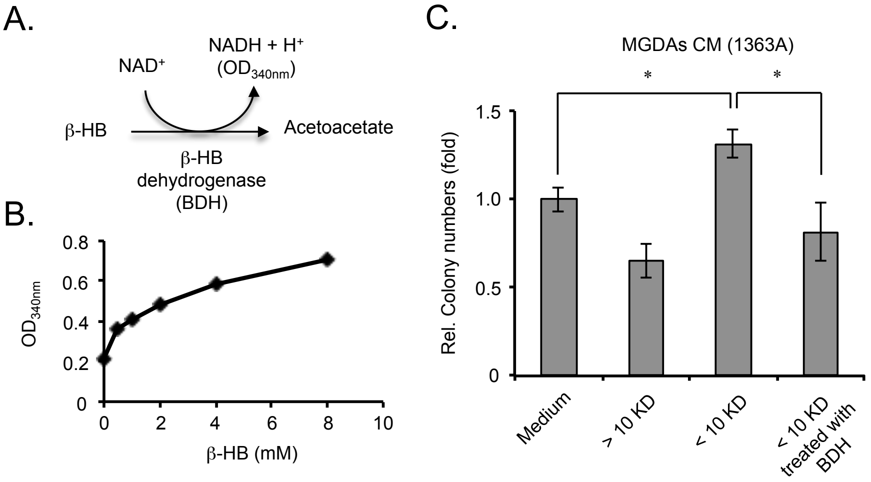

Supplementary Figure 4. Soft agar colony formation assays using β-HB depleted MGDAs-conditioned medium

(A) β-HB could be converted to acetoacetate by the enzyme beta-hydroxybutyrate dehydrogenase (BDH). The BDH activity could be monitored by the production of NADH at 340 nm (OD_{340nm}). (B) The dynamic range of BDH catalyzing enzymatic reaction. 0, 0.5, 1, 2, 4, and 8 mM β-HB (1ml) were converted to acetoacetate by BDH (10 U/ml, 20 μl) in the presence of NAD^+ (50 mM, 20 μl) at 37 °C for 1h. (C) Removal of the β-HB in fractionated MGDAs-CM (< 10 kD) decreased the promoting activity in soft agar colony formation using MDA-MB-231 breast cancer cells. Data show means ± s.d. *, $p < 0.05$ (Student's t-test).

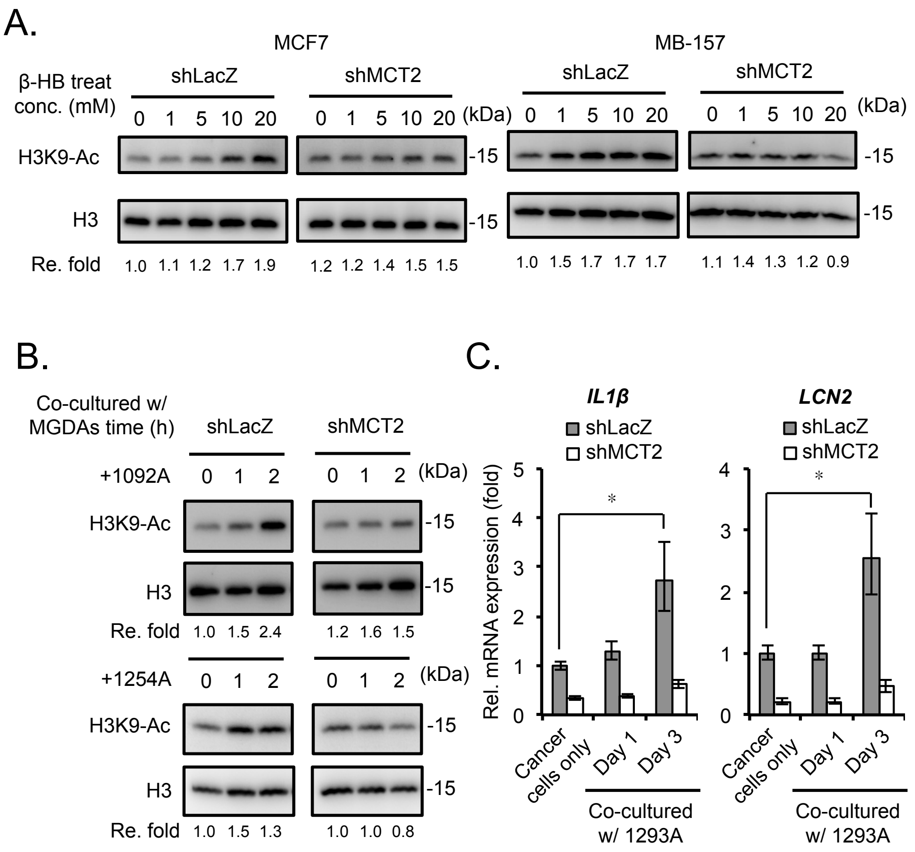

Supplementary Figure 5. Treatment with β-hydroxybutyrate and co-culturing with MGDAs both increase H3K9 acetylation and induce expression of *IL-1β* and *LCN2* in MCT2-expressing breast cancer cells

(A) The levels of H3K9 acetylation were increased when MCF7 and MDA-MB-157 cells were treated with β-hydroxybutyrate in the presence of MCT2. (B) Co-culture with MGDAs increased the histone H3 Lys 9 (H3K9) acetylation in MCT2-expressing cells. (C) The expression levels of *IL-1β* and *LCN2* were induced upon MGDAs co-culture. The experiment in C was performed in technical triplicate and repeated at least twice with similar results. Data show means ± s.d. *, $p < 0.05$ (Student's t-test).

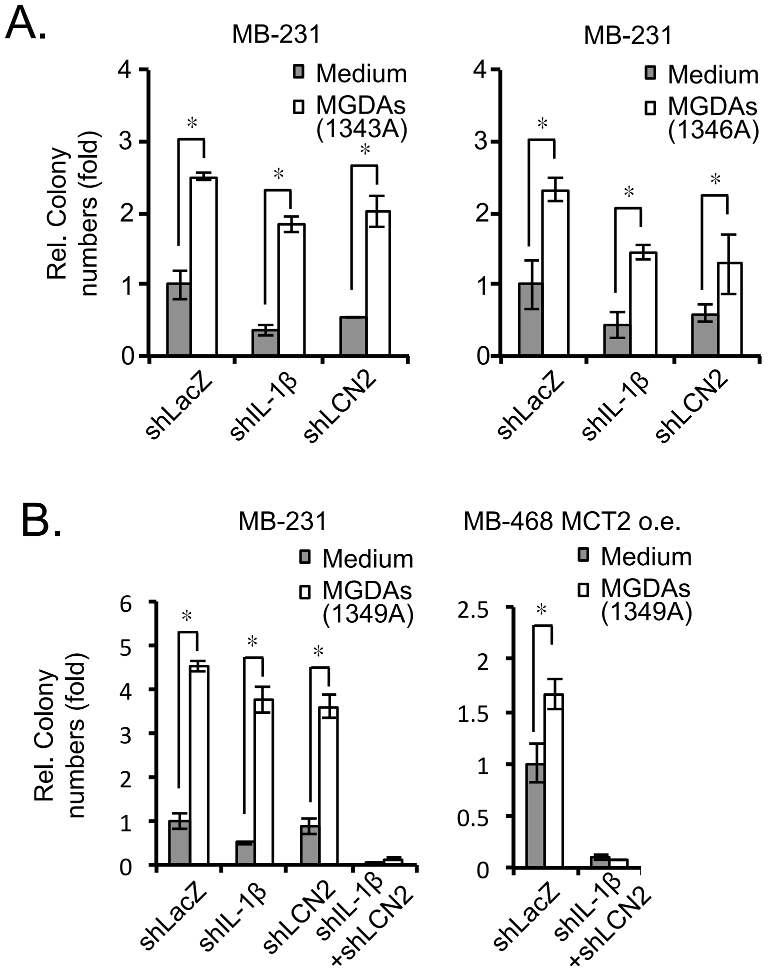

Supplementary Figure 6. Concurrent depletion of *IL-1β* and *LCN2* abolishes MGDAs-mediated colony promotion

(A) Depletion of *IL-1β* and *LCN2* alone only slightly abrogated the colony promotion mediated by MGDAs co-culture. (B) Double knockdown of *IL-1β* and *LCN2* completely abrogated the increase of colonies induced by MGDAs co-culture in MDA-MB-231 and MDA-MB-468 MCT2-overexpressing cells. The experiments in A and B were performed in technical triplicate and repeated at least twice with similar results. Data show means ± s.d. *, $p < 0.05$ (Student's t-test).

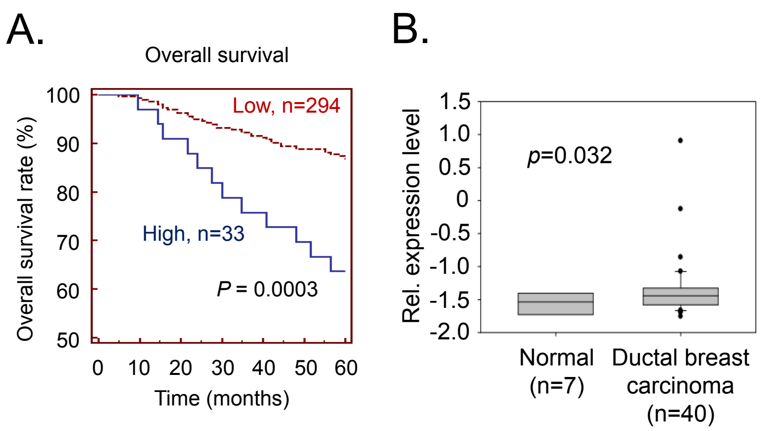

Supplementary Figure 7. The expression of *MCT2* correlates with poor prognosis and is upregulated in ductal carcinoma

The expression of *MCT2* was associated with poor prognosis (A) and higher in breast cancer tissues compared to normal tissues (B). (Data was adapted from the breast tumor gene expression profiling done by Kao *et al*, BMC Cancer, 2011, 11 (143) and Richardson *et al*, Cancer cell, 2006, 9(2): 121-132, respectively, http://www.oncomine.org)

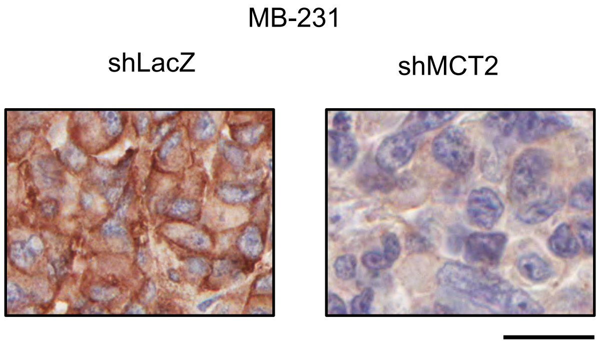

Supplementary Figure 8. Immunohistochemistry staining of MCT2 in MDA-MB-231 shLacZ and shMCT2 xenograft tumors

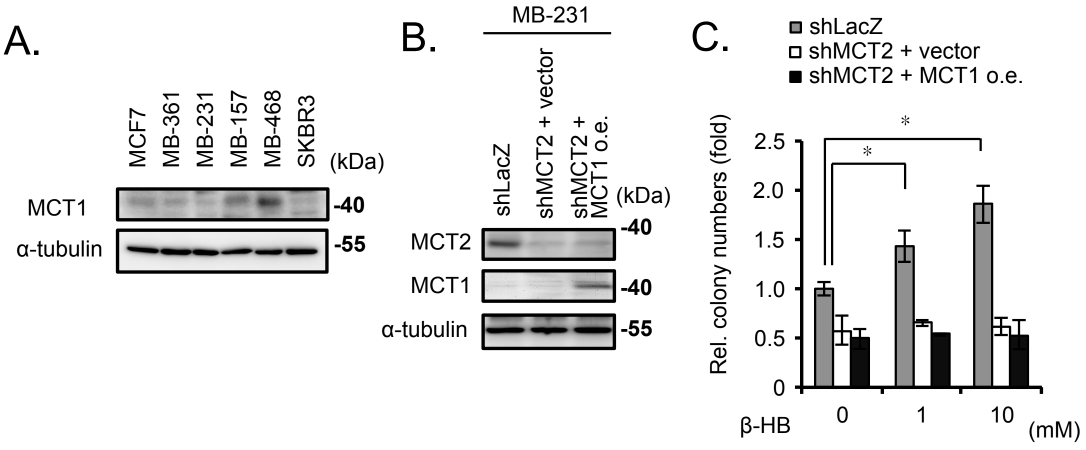

Supplementary Figure 9. Ectopic expression of MCT1 fails to rescue the response to β-hydroxybutyrate supplement in MCT2-depleted MDA-MB-231 cells

(A) Western blot analysis of MCT1 in six different breast cancer cell lines. (B) Overexpression of MCT1 in MCT2-depleted MDA-MB-231 cells. (C) Ectopic expression of MCT1 in MCT2-depleted MDA-MB-231 cells did not rescue the response to β-hydroxybutyrate treatment in soft agar colony formation assays. The experiment in C was performed in triplicate and repeated at least twice with similar results. Data show means ± s.d. *, $p < 0.05$ (Student's t-test).

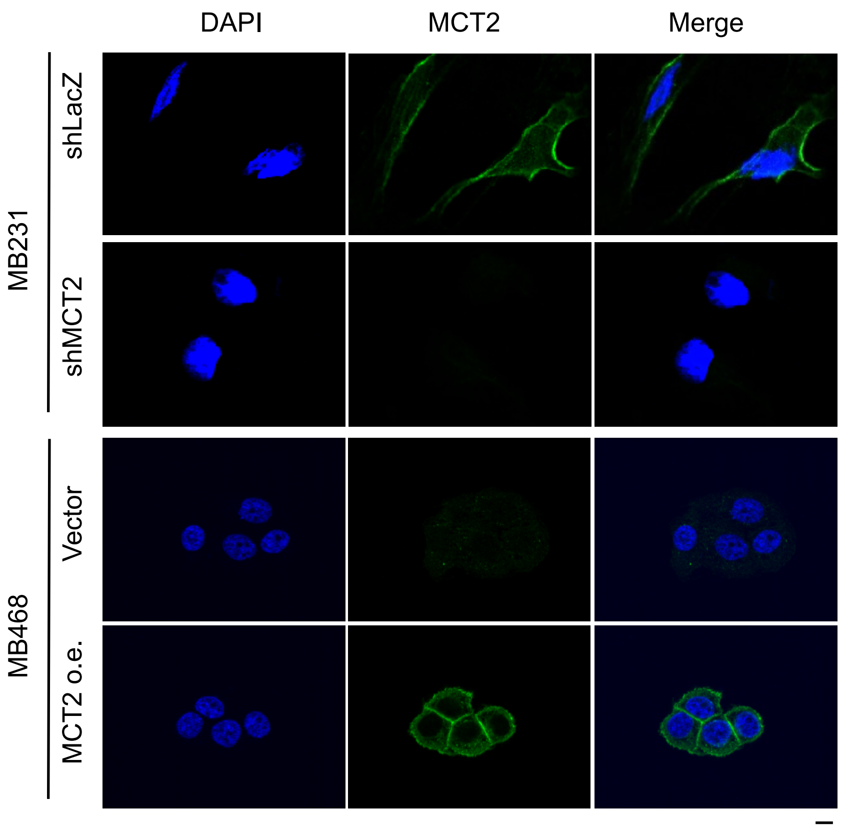

Supplementary Figure 10. Immunofluorescence staining of MCT2 in breast cancer cells

The intracellular location of MCT2 was determined by IF staining in MDA-MB-231 (Upper panel, shLacZ vs. shMCT2) and MDA-MB-468 breast cancer cells (Lower panel, vector control vs. MCT2 o.e.). Bar, 10 μm

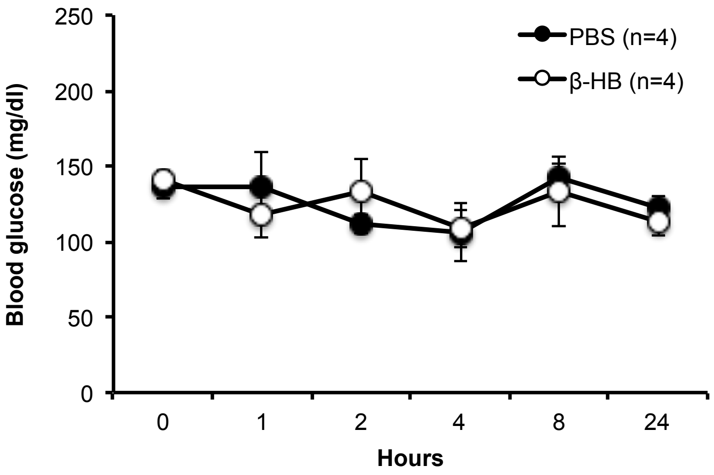

Supplementary Figure 11. Blood glucose levels after intraperitoneal administration of β-hydroxybutyrate in mice

Blood glucose levels were determined at the indicated time following the administration of β-hydroxybutyrate (500 mg/kg/). (PBS group, n=4; β-HB group n=4). Data show means ± s.d.

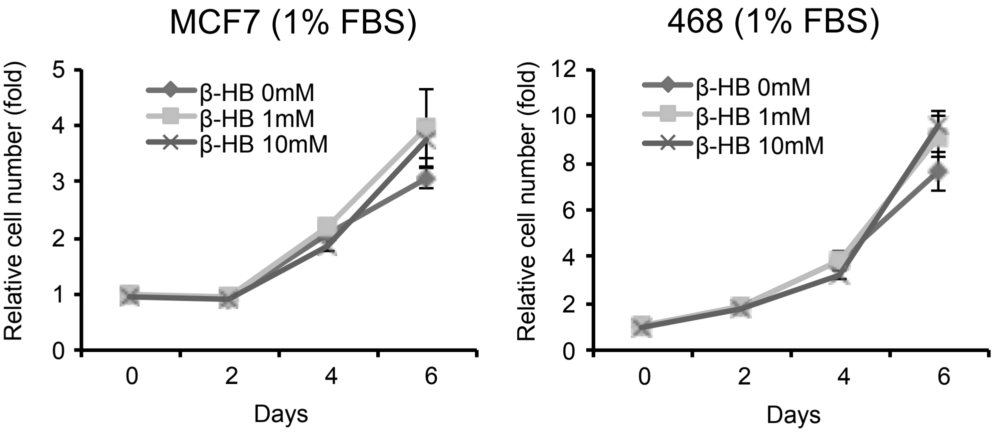

Supplementary Figure 12. Proliferation of breast cancer cells was not influenced upon β-hydroxybutyrate treatment

The relative proliferation rate of MCF7 and MDA-MB-468 cells were determined by MTT assay upon different dosages of β-hydroxybutyrate treatment. Data show means ± s.d.

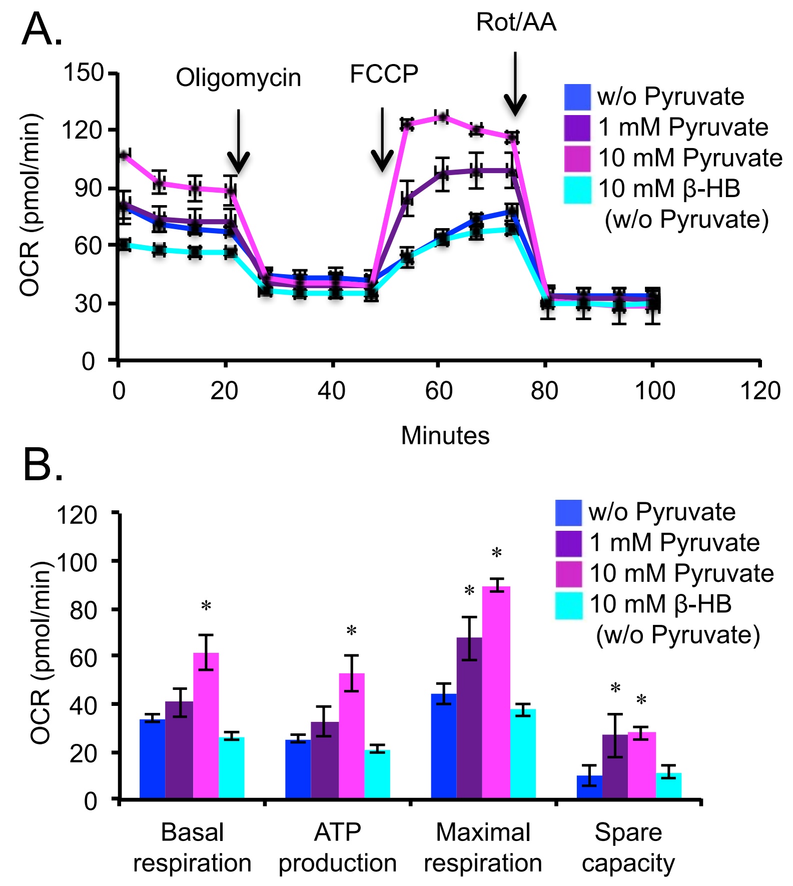

Supplementary Figure 13. β-hydroxybutyrate would not be a preferential mitochondrial fuel in breast cancer cells

(A) Raw data of oxygen consumption rate (OCR) determined by the Seahorse XFe96 analyzer. MDA-MB-231 cells were seeded in pyruvate free condition. Different concentrations of pyruvate and/or β-hydroxybutyrate were added and then incubated for 1 hr before Seahorse measurement. (B) Analysis of the Seahorse data. Pyruvate, but not β-hydroxybutyrate efficiently increased basal respiration, ATP production, maximal respiration, and spare capacity of MDA-MB-231 cells. The experiment was performed in technical triplicate. Data show means ± s.d. *, $p < 0.05$ (Student's t-test).

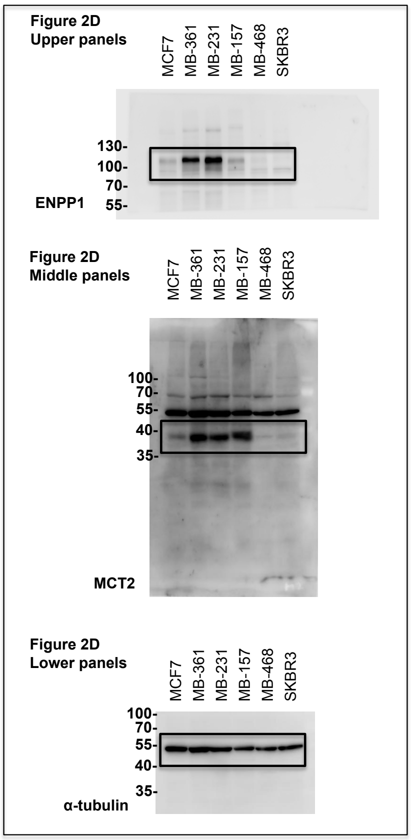

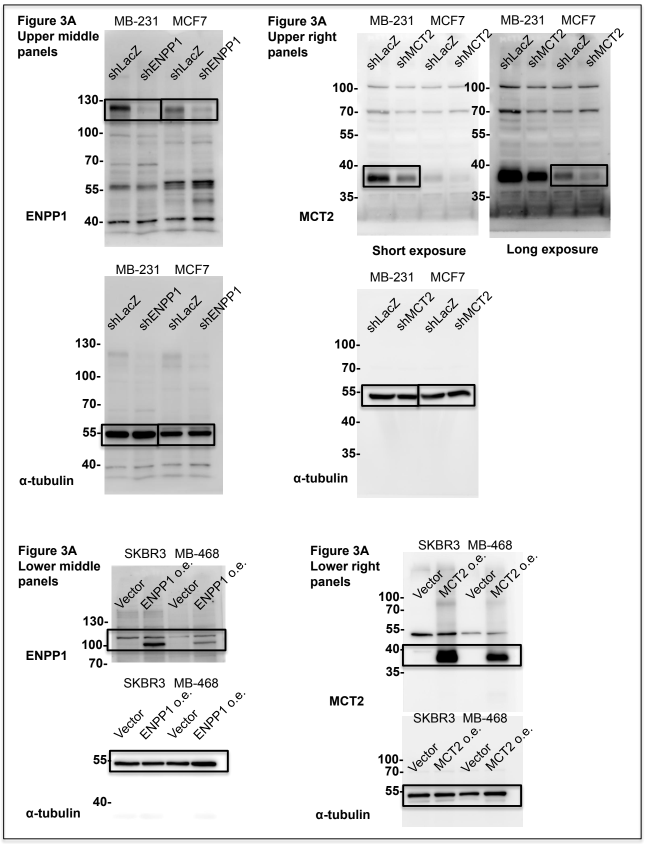

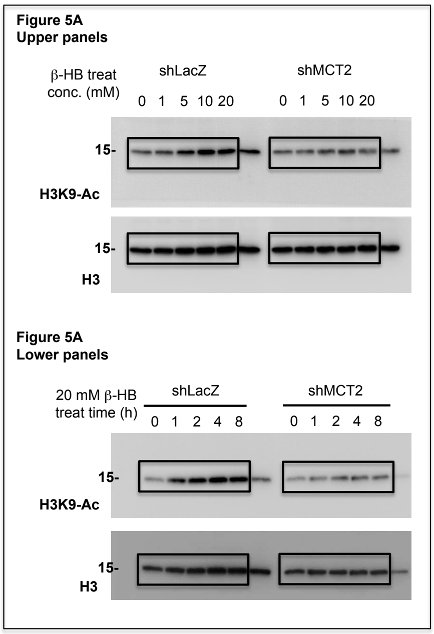

Supplementary Figure 14. Uncropped scans of immuno-blots shown in the main figures

Boxes indicate the cropped areas used in the figure.

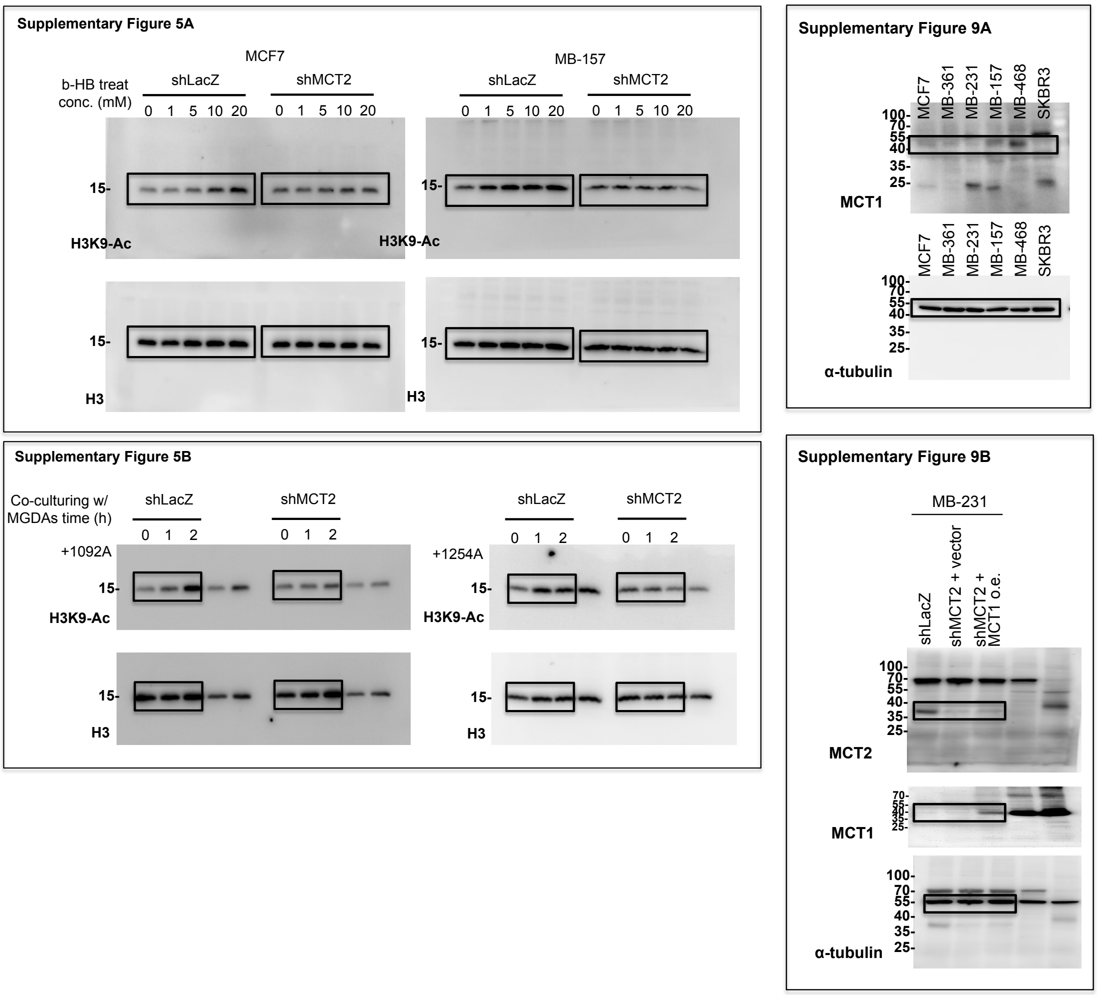

Supplementary Figure 15. Uncropped scans of immuno-blots shown in the supplementary figures

Boxes indicate the cropped areas used in the figure.

Supplementary Table 1. Characteristics of patients and tumors in this study (cohort 1, Q-PCR)

Factors	No of patients (%) n = 106
Age (years old)	
≤ 50	59 (55.7 %)
> 50	47 (44.3 %)
Tumor size (cm)	
≤ 2	23 (21.7 %)
> 2	82 (77.4 %)
unknown	1 (0.9 %)
Lymph node status	
0	29 (27.4 %)
1-3	34 (32.1 %)
≥ 4	36 (33.9 %)
unknown	7 (6.6 %)
Grade	
1	28 (26.4 %)
2	53 (50.0 %)
3	13 (12.3 %)
unknown	12 (11.3 %)
Stage	
0 & I	14 (13.2 %)
II	67 (63.2 %)
III	18 (17.0 %)
IV	4 (3.8 %)
unknown	3 (2.8 %)
ER	
(+)	68 (64.2 %)
(-)	35 (33.0 %)
unknown	3 (2.8 %)
PR	
(+)	54 (50.9 %)
(-)	49 (46.3%)
unknown	3 (2.8 %)

Supplementary Table 2. Characteristics of patients and tumors in this study (cohort 2, IHC)

Factors	No of patients (%) n = 36
Age (years old)	
≤ 50	10 (27.8 %)
> 50	26 (72.2 %)
Grade	
1	3 (8.3 %)
2	19 (52.8 %)
3	14 (38.9 %)
TNM Stage	
0 & I	4 (11.1 %)
II	22 (61.1 %)
III	10 (27.8 %)
IV	0 (0 %)
ER	
(+)	27 (75 %)
(-)	9 (25 %)
PR	
(+)	18 (50 %)
(-)	18 (50 %)
HER2	
(+)	8 (22.2 %)
(-)	28 (78.8 %)

Supplementary Table 3. Characteristics of patients and tumors

(Data was adapted from the breast tumor gene expression profiling by Kao *et al*, BMC Cancer, 2011, 11 (143), http://www.oncomine.org)

Factors	No of patients (%) n = 327
Age (years old)	
≤ 50	209 (63.9 %)
> 50	118 (36.1 %)
T status	
T1	99 (30.3 %)
T2	188 (57.5 %)
T3	26 (7.9 %)
T4	12 (3.7 %)
unknown	2 (0.6 %)
N status	
N0	137 (41.9 %)
N1	87 (26.6 %)
N2	63 (19.3 %)
N3	40 (12.2 %)
M status	
M0	319 (97.6 %)
M1	8 (2.4 %)
TNM stage	
I	67 (20.5 %)
II	147 (45.0 %)
III	103 (31.5 %)
IV	8 (2.4 %)
unknown	2 (0.6 %)
ER	
(+)	204 (62.4 %)
(-)	123 (37.6 %)
HER2	
(+)	75 (22.9 %)
(-)	252 (77.1%)

Supplementary Table 4. Univariate and multivariate proportional hazards analysis of the influence of MCT2 expression on the overall survival of 327 breast cancer patients

(Data was adapted from the breast tumor gene expression profiling by Kao *et al*, BMC Cancer, 2011, 11 (143), http://www.oncomine.org)

Variables	Univariate HR (95% CI)	*P*	Multivariate HR (95% CI)	*P*
MCT2 high-risk group (vs. low-risk group)	3.13 (1.64-5.97)	**0.0005**	2.03 (1.01-4.08)	**0.049**
Age (≥ 46 years old)[a]	0.84 (0.49-1.45)	0.5363	1.06 (0.60-1.87)	0.8510
ER (vs negative group)	0.38 (0.22-0.67)	**0.0007**	0.51 (0.26-1.02)	0.0581
HER2 (vs negative group)	2.35 (1.35-4.12)	**0.0028**	1.42 (0.74-2.70)	0.2865
T status (per grade)[b]	2.04 (1.49-2.80)	**<0.0001**	1.48 (0.95-2.32)	0.0842
N status (per grade)[c]	1.92 (1.50-2.46)	**<0.0001**	1.62 (1.22-2.14)	**0.0008**
M status (per grade)[d]	4.52 (1.63-12.50)	**0.0038**	1.92 (0.52-7.09)	0.3321

[a]Median age = 46 years old; [b]T status, T1-T4; [c]N status, N0-N3; [d]M status, M0-M1

HR: hazard ratio, CI: confidence interval

Supplementary Table 5. Primer sequences for cloning

Gene		Sequence (5’-3’)
ARMCX1	forward	GATCACTAGTATGGGCCGCACTCGGGAA
	reverse	GATCGCGGCCGCTTAGAGTTTGGTTAATACTTTC
ENPP1	forward	GATCCTCGAGATGGAGCGCGACGGCTGC
	reverse	GATCTCTAGATCAGTCTTCTTGGCTAAAGGT
MCT2	forward	GATCCTCGAGATGCCACCAATGCCAAGT
	reverse	GATCTCTAGATTAAATGTTAGTTTCTCTTTC

Supplementary Table 6. Target sequences of shRNA

Gene	Clone ID	Target sequence (5’-3’)
ABCG2	TRCN59798	GCCTCGATATTCCATCTTCAA
	TRCN59799	GCAACAACTATGACGAATCAT
	TRCN59800	CCTTCTTCGTTATGATGTTTA
	TRCN59801	GCTGTGGCATTAAACAGAGAA
	TRCN59802	CCTGCCAATTTCAAATGTAAT
AREG	TRCN117993	GCCGACTATGACTACTCAGAA
	TRCN117995	GAACGAAAGAAACTTCGACAA
	TRCN117996	GAACCACAAATACCTGGCTAT
ARMCX1	TRCN133686	GCTAAGACTGTTAACCAACAT
	TRCN138265	CTTGGTCACTCTGGGTAACAA
	TRCN160564	CTTCTTAATATCCTTACCCTA
	TRCN160630	CACTTCACCAAGATACAGATT
	TRCN160631	CAGAATTGATTTCCCTCTTTA
	TRCN161844	GCAGTTGGTACGATGTGATTT
ATP2B1	TRCN43068	GCCTACAATTTACCTTGTTAA
	TRCN43069	CCAGAGAAAGAGGGTGGATTA
	TRCN43070	GCCTCTCATCTCACGTACAAT
	TRCN43071	GCAGATTTAGAAAGAAGAGAA
ENPP1	TRCN2537	CGTGCATAGAACCAGAACATA
	TRCN2540	CTGCGAAAGTATGCTGAAGAA
FRAS1	TRCN73564	CCCTCACATCTCTCTTACCAA
	TRCN73566	GTGCCAAATGTCTGTGTAGAA
	TRCN73567	CGTTGGCGGAATTTGCAGTAT
FRMD5	TRCN128657	CATCTTTCCAATTTGCAGGAT
	TRCN131096	GAGGAGGAGAAGGAATCTGAA
GPC6	TRCN123094	GCTTCCTCTTTCCTTCAGCTA
	TRCN123095	GCCATTATGAACATGCAAGAA
	TRCN123096	GCCAACAAAGCAAACTCGAAT
	TRCN123097	GCACAGCAAAGCCAGATACTT
	TRCN123098	ACCCGATAGATGTCAAGATTT

Supplementary Table 6. Target sequences of shRNA (continued)

Gene	Clone ID	Target sequence (5'-3')
GPR126	TRCN11562	CCTATCTTACATCCAAATCTA
	TRCN273811	ACTTAACCTCAGCCAATATTA
	TRCN273865	CCTATCTTACATCCAAATCTA
IL6R	TRCN372671	ATGCATCCGCCGTACTCTTTG
	TRCN372728	CTGGACCCTGTGGATGATAAA
	TRCN378748	TATCGGGCTGAACGGTCAAAG
OSBPL8	TRCN281229	GTGACACAGATACATCAGAAA
RAB8B	TRCN379718	ACCTGTTGCAGAAGCGGTTAT
	TRCN380132	TTACTGCCTTGGTAGCATTTA
	TRCN380248	GTCGTGAAGTTCTAGACAAAT
	TRCN380390	AGAAGCTAGCAATTGACTATG
	TRCN382238	GAATGATCCTGGGTAACAAAT
RFTN1	TRCN130134	CGACTCTTCTGCTTTGACAAA
	TRCN130387	GCGATTCTCCAGAGAAGAAAT
	TRCN130642	CCAGAAACTCATCCCAGAGTT
RHOF	TRCN48050	CAACGTCCTCATCAAGTGGTT
	TRCN48051	GCTCATCTGCTATGACGTCAT
MCT2 (SLC16A7)	TRCN38508	CCTTGAGCAAATCTAAACATT
	TRCN286881	CCTTGAGCAAATCTAAACATT
	TRCN294347	CAACCCGCCTTAACCATAATT
THBD	TRCN53923	GCCGATGTCATTTCCTTGCTA
	TRCN53924	CCAGTGGGTTACGGGAGACAA
	TRCN53925	CTTGCTCATAGGCATCTCCAT
	TRCN53927	CTTCCTCAATGCCAGTCAGAT
	TRCN174209	GCTAGCTGTGAGTGCCCTGAA
IL-1β	TRCN358600	CTGACTTCACCATGCAATTTG
	TRCN358661	TCACCTCTCCTACTCACTTAA
LCN2	TRCN60290	GTACTTCAAGATCACCCTCTA
	TRCN372769	CAATTCTCAGAGAAGACAAAG

Supplementary Table 7. Primer sequences for real-time PCR

Gene		Sequence (5'-3')
ABCG2	forward	CACAAGGAAACACCAATGGCT
	reverse	ACAGCTCCTTCAGTAAATGCCTTC
AREG	forward	TGCTGGATTGGACCTCAATG
	reverse	TCCCGAGGACGGTTCACTAC
ARMCX1	forward	CAACATCCTGGAGCGAACAA
	reverse	ACGTATGGCATTCTGGTTAAATGA
ATP2B1	forward	GAGCTGCGGGCTCTCATG
	reverse	TGGTGCAAATTCCATAGACATCTC
ENPP1	forward	AGTGGCAACTTGCATTGAATCC
	reverse	CCAACAAAGAGGGCTTGCAT
FRAS1	forward	GGCAAAATACACACCCCTAGTCTT
	reverse	GGGAGTGCTCACAACATGAAATAG
FRMD5	forward	CAAGCCAAGTCCGCACAGT
	reverse	GCACTTGATTCCATGACTTCCTT
GPC6	forward	CAGGCTGACCTCGACACAGA
	reverse	GGTCCATGACCGACTCAATGT
GPR126	forward	ACCTGGGGACCCTCTGTCAAGATG
	reverse	AGCCATTCTGCCACCTTGCTCTG
IL6R	forward	ATGCGACAAGCCTCCCAGTGC
	reverse	GCAATGCAGAGGAGCGTTCCGA
OSBPL8	forward	GAAGGTGCTGGATGGATGCT
	reverse	GAAACGCTCAGGTCATGTTCCT
RAB8B	forward	CAGGAGCAGGTGGACCAGTGA
	reverse	TGAGGCTGGGTGACCTGTGCT
RFTN1	forward	AACGCACGTGGGGATCACGC
	reverse	GGGTGAGCTGGGCTGCTTGG
RHOF	forward	CCGGGCAGGAAGGAGCTGAAGA
	reverse	CCTTGCTGCCAACGGTCACG
MCT2	forward	TGGCCCAGTTCTTCTTGGCCC
	reverse	GCCACACGCTTGCTGCTACC
THBD	forward	ACCGGTGCGAGGACGTGGAT
	reverse	CTGAAGCACGGGTCCACGGG

Supplementary Table 7. Primer sequences for real-time PCR (continued)

Gene		Sequence (5'-3')
GAPDH	forward	ACCCAGAAGACTGTGGATGG
	reverse	TCTAGACGGCAGGTCAGGTC
IL-1β	forward	GCTGCTCTGGGATTCTCTTCA
	reverse	GGCCATCAGCTTCAAAGAACA
LCN2	forward	GAGTTACCTCGTCCGAGTGG
	reverse	TTGGTTCTCCCGTAGAGGGT
OLR1	forward	ACTGGGAAAAGAGCCAAGAGAA
	reverse	TGGATGAAGTCCAGATCAGC

Supplementary Table 8. Primer sequences for Chromatin immunoprecipitation (ChIP)

Promoter Region		Sequence (5'-3')
IL-1β (-100~+1)	forward	AACTTGCCAGGTGTTTCAGG
	reverse	GGCCATCAGCTTCAAAGAAC
IL-1β (-200~-100)	forward	GGGAGAGGGAGAGCTCAGAT
	reverse	TCTCAAAGCTGCCTGAAACA
IL-1β (-400~-200)	forward	TACAAGTCCCTCCAGCCTTG
	reverse	GCTCCCTGTTGGATCTTGAG
IL-1β (-600~-400)	forward	GAGCTCGCCAGTGAAATGAT
	reverse	CCTTGCAACAACACATCTGG
IL-1β (-800~-600)	forward	TCTGCTCCAGCTCTCCTAGC
	reverse	GGCGAGCTCAGGTACTTCTG
IL-1β (-1000~-800)	forward	TCTCCCTCTCCTTCCCTCTC
	reverse	TTGCTAAACCAAACCCCAAC
LCN2 (-100~+1)	forward	TGCAGAAATCTTGCCAAGTG
	reverse	ATTTCAGGGCCGAGGAAG
LCN2 (-200~-100)	forward	AAGGAAGGCACAGAGGGAGT
	reverse	GGGATCTAGGGTGGGTTGAT
LCN2 (-400~-200)	forward	ACCCTTCCCTGACCCTTAAA
	reverse	ACTCCCTCTGTGCCTTCCTT
LCN2 (-600~-400)	forward	GCACTCACAGGAGAGGAAGG
	reverse	TCATGTGCTGTTTCCTGGTT
LCN2 (-800~-600)	forward	TCAGTGACCCAAAGCAACAG
	reverse	CCTTCCTCTCCTGTGAGTGC
LCN2 (-1000~-800)	forward	CTCATGGGTGAGCCATCTCT
	reverse	AGTGCAAGGATCTGGCCTTA
