# Supplementary material for: Adipocytes promote malignant growth of breast tumours with monocarboxylate transporter 2 expression via β-hydroxybutyrate
Source: Nat Commun. 2017 Mar 10;8:14706. doi: 10.1038/ncomms14706 (PMC5353665; doi:10.1038/ncomms14706)
Supplement: Peer Review File [file ncomms14706-s3.pdf]

Reviewers' comments:

Reviewer #1 (Remarks to the Author):

The manuscript "β-hydroxybutyrate secreted from adipocytes of mammary glands promotes malignant growth of breast tumor with monocarboxylate transporter 2 expression" addresses the role of adipocytes in breast cancer. The authors show that mammary gland-derived adipocytes (MGDAs) promote growth of breast tumors that express monocarboxylate transporter 2 (MCT2) that transports transports β-hydroxybutyrate. The authors link adipocyte-derived β-hydroxybutyrate signaling with increased histone H3K9 acetylation in cancer cells and with up-regulation of IL1β and LCN2, which may have a clinical relevance.

First of all, the term tumorigenesis is misused in the manuscript. This study does not address cancer initiation - it is cancer progression that they get at.
The role of adipocytes in breast cancer progression has been studied in over a hundred of publications. The discussion of other mechanisms through which adipocytes promote cancer is largely lacking in the manuscript. β-hydroxybutyrate physiology is not well introduced. The use of HDAC inhibitors for cancer treatment is also not well discussed.

It is not clear if there is a substantial reason to consider that adipocyte-derived β-hydroxybutyrate (β-HB) plays a role in cancer. How does circulating β-HB concentration in ketosis compare to concentrations in adipocyte-conditioned medium? β-HB is known to be mainly produced by the liver. The concentration of circulating blood β-HB rises from ~0.1 mM observed in normal fed state to ~1 mM after few hours of fasting, and up to 5-7 mM after prolonged starvation (Cahill, 1970; Robinson and Williamson, 1980; Laffel, 1999; Cahill and Veech, 2003). Does fasting promote breast cancer progression? That would be expected if the claims in the manuscript are true. Ketone supplementation decreases tumor cell viability and prolongs survival of mice with metastatic cancer (Int J Cancer. 135(7): 1711-1720, 2014). How do authors reconcile this observation?

The mechanism proposed in the study is novel. However, it is not sufficiently substantiated by data. While there are reports that β-HB serves as an inhibitor of class I histone

deacetylases (HDACs) (Shimazu et al., 2013), it is certainly not its main function. β-HB is a ketone body used as a preferred energy source for myocytes and other cell types. It is possible that it could be used as fuel by cancer cells. Without investigating this more apparent mechanism thoroughly, the manuscript does not stand.

There are also issues with data.

In Fig. 5, pyruvate (ketone body/ nutrient) and butyrate (HDAC inhibitor) should be used as controls.

Supplementary Fig. S1A, image resolution is too low. MGDAs purity needs to be presented. In Fig 1B and C, adipocytes from healthy women would be important to show as a control.

GPC6 expression is the highest in ER-positive breast cancer cell lines, but not expressed in ER-negative MDA-MB-468 cell line (Fig. 3s). It has been reported that GPC6 enhances migration and invasion of breast cancer cells [Biochem J. 2011 Nov 15; 440(Pt 1): 157-166]. Depletion of GPC6 abolished the colony number increase (Fig. 3). However, there was no data to exclude GPC6 effects in clone formation. Fig. 3 lacks ARMCX1, ENPP1 and MCT2 ectopically expressing data in mRNA or protein level.

In colony formation assay, culture medium contains 1mM pyruvate. This condition may conceal the effect of pyruvate (Figure S3 D). It was reported that Pyruvate fuels mitochondrial respiration and proliferation of breast cancer cells. Biochem J. 15;444 (3):561-71, 2012. Pyruvate-free medium should be used in this assay. Pyruvate or b-HB analogs such as butyrate, amy-butyrate etc. should be used as controls for in vivo tumor studies (Fig. 4).

Inhibition of HDAC by β-HB was correlated with global changes in transcription. IL-1 may not be a direct target of b-HB. High glucose also induces IL-1 in cell culture.

In the experimental system used, it is possible that molecules other than b-HB, such as FFA or lysophospholipids, leptin and IGF-1, stimulate breast cancer cells. Comparing the effects to those of adipocyte-derived molecules reported to stimulate cancer cells would be

important.

Some labels in Figs. 1, 3, 4 and 7 are missing.

Reviewer #2 (Remarks to the Author):

This manuscript reports that b-hydroxybutyrate secreted by mammary gland-derived adipocytes promotes the growth of breast cancer cells in vitro and in vivo and that this is dependent on the cancer cells expressing MCT2 and is mediated by upregulation of IL1b and lipocalin 2 (LCN2). LCN2 is well known to promote tumorigenesis, thus, this part is not novel. Overall it's a fairly comprehensive study, but there are numerous issues that need to be addressed.

Specific comments:

1. The MDA-MB-361 cell line is not an ER+ line, but a luminal HER2+. There are many real ER+ cell lines to be used like T47D and ZR-75-1.

2. The concentration of b-hydroxybutyrate needed to stimulate the growth of breast cancer cells is very high - 1-10mM. Is this anywhere close to the physiologic range in the mammary gland?

3. MCT2 is also known as a key transporter of pyruvate and lactate - both of which are highly relevant to tumor growth. Can the authors exclude the possibility that these metabolites also play a role in adipocyte-mediated tumor promotion?

4. There are small molecule inhibitors of MCT proteins. It would be useful to try some of these in a larger panel of breast cancer cell lines to see if this mimics the MCT2 shRNA effects.

5. What is the half life and PK characteristics of b-hydroxybutyrate in the mouse? Can 1-

10mM levels be achieved in the tumors by intraperitoneal injection?

6. It appears that IL1b and LCN2 are essential for breast cancer growth regardless of the adipocytes - at least this is what Figure 6B shows. Thus, the authors' interpretation is not correct.

7. Both IL1b and LCN2 act through receptors - do the presence of these on breast cancer cells correlates with response to adipocytes?

8. Figure 7A-B: For clinical outcome analysis ER+, HER2+, and basal-like breast tumors have to be analyzed separately, since they have very different outcomes. It is puzzling how the authors can get such significant p values in multivariate analysis using such a small and highly heterogeneous cohort. The same criticism is true for the survival data in Figure 7F.

9. The immunohistochemical staining in Figure 7C seems to show myoepithelial staining in the normal breast although overall the staining quality is very poor. The authors need to provide evidence for the specificity of the antibody (use knock out or shMCT2 controls) and provide better quality images.

10. The differences between control and experimental groups are modest in numerous figures. It would be important to know how many replicates were included in each experiments and how many independent experiments reproduced the findings.

Reviewer #3 (Remarks to the Author):

In the present study, the authors describe the role of MCT2 in promoting breast cancer malignancy via crosstalk with mammary gland-derived adipocytes.

The results are original, with interest to scientists in the cancer field and, importantly, could have potential clinical application.

The manuscript is presented in a clear way, with a good abstract and introduction. The experimental approaches are adequate; the quality of data is good, the authors provide strong evidence for their conclusions and references are adequate.

Suggested improvements:

- Fig.2D. Western-blot results should be also presented for ARMCX1 gene.
- Fig. 2. Since the function of MCT2 as a plasma membrane transporter is being investigated, the authors should also present evidence for its presence at the plasma membrane (IF/cell fractionation/...) of the cell lines.
- Results. Description of Fig. 3B. The sentence "... only MCT2, but not ARMCX1 nor ENPP1, enhanced colony formation (Figure 3B)" should be rephrased since there is also enhancement of colony formation for ENPP1 in SKBr3 cells.
- Figure 6C. Are the squares in the figure supposed to be there? Please check.
- The cutoff for MCT2 positivity in clinical cases was considered as 5% of membrane staining and only 7/36 cases were presented positive. Why did the authors choose 5%? What is the biological significance of 5% positivity in a tissue sample? This cutoff should be justified.
- With exception of 231 cells, the breast cancer cells used also express MCT1, which is also able to transport β-hydroxybutyrate. Why is not MCT1 involved in β-hydroxybutyrate transport in breast cancer cells? Is it a matter of concentration, owing to the different affinities of the transporters? This issue should be discussed.

Detailed point-by-point response to reviewers' comments (bold)

Reviewer #1 (Comments to the Author):

The manuscript "β-hydroxybutyrate secreted from adipocytes of mammary glands promotes malignant growth of breast tumor with monocarboxylate transporter 2 expression" addresses the role of adipocytes in breast cancer. The authors show that mammary gland-derived adipocytes (MGDAs) promote growth of breast tumors that express monocarboxylate transporter 2 (MCT2) that transports transports β-hydroxybutyrate. The authors link adipocyte-derived β-hydroxybutyrate signaling with increased histone H3K9 acetylation in cancer cells and with up-regulation of IL1β and LCN2, which may have a clinical relevance.

Ans: We appreciate the critical suggestions from the reviewer for this work.

Comments:

1. **First of all, the term tumorigenesis is misused in the manuscript. This study does not address cancer initiation - it is cancer progression that they get at.**

Answer: We thank the reviewer's suggestion. We have replaced the term tumorigenesis with tumor progression in the revised main text.

2. **The role of adipocytes in breast cancer progression has been studied in over a hundred of publications. The discussion of other mechanisms through which adipocytes promote cancer is largely lacking in the manuscript.**

Answer: By reading several decent review articles such as (Park et al., 2014; Tan et al., 2011; Vona-Davis and Rose, 2007), we have discussed the other potential mechanisms through which adipocytes affect in breast cancer progression. For example, the importance of adipocyte-secreted factors and hormones was described in the introduction and discussion, respectively.

3. **β-hydroxybutyrate physiology is not well introduced.**

Answer: We thank the reviewer's suggestion. We have described and discussed the physiology of β-hydroxybutyrate in this revised manuscript. Please read the discussion parts.

4. **The use of HDAC inhibitors for cancer treatment is also not well discussed.**

Answer: We have discussed the use of HDAC inhibitor for cancer treatment in the discussion part in this revised manuscript.

5. **It is not clear if there is a substantial reason to consider that adipocyte-derived β-hydroxybutyrate (β-HB) plays a role in cancer.**

Answer: First, we observed that the expression of MCT2 increased the response to the MGDAs-mediated tumor promotion. Second, we found that supplementing with <10 kD fraction of MGDAs-conditioned medium enhanced the colony formation in a MCT2-dependent manner. Third, we have individually tested the effect of pyruvate, lactate, and β-hydroxybutyrate (the known monocarboxylates transported via MCT2) in soft agar colony formation. As shown in the original Figure 4D and S3C, we observed that β-hydroxybutyrate and lactate enhanced the colony formation of MDA-MB-231 cells, but not MCT2-knockdown MDA-MB-231 cells. Supplementing with β-hydroxybutyrate, but not pyruvate or lactate, was also observed to significantly enhance colony formation in MCT2-overexpressing MDA-MB-468 cells (Figure 4D and S3C). Moreover, administration of β-hydroxybutyrate via daily intra-peritoneal (i.p.) injection in mouse xenograft models promoted MCT2-expressing tumor growth. Thus, β-hydroxybutyrate play a more important role than lactate and pyruvate in MCT2- and MGDAs-mediated promotion of tumor progression.

Original Figure 4D

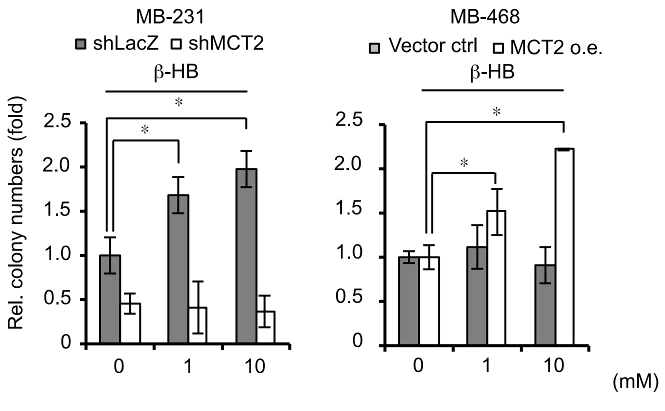

Original Figure 4D: MCT2-depleted MDA-MB-231 and overexpressing MDA-MB-468 cells were treated with various doses of β-hydroxybutyrate in soft agar colony formation assays

Original Figure S3C

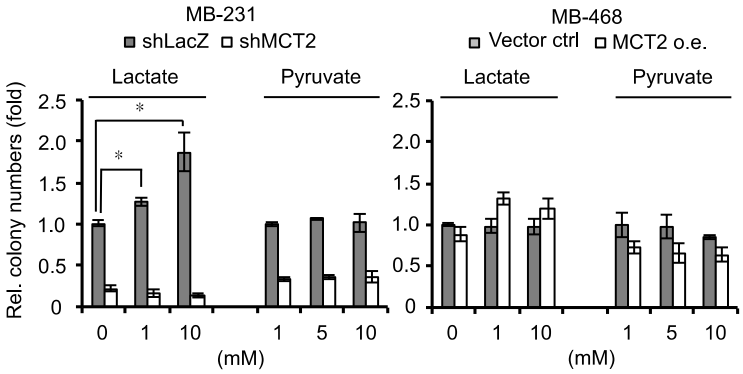

Original Figure S3C: MCT2-depleted MDA-MB-231 and overexpressing MDA-MB-468 cells were treated with various doses of lactate and pyruvate in soft agar colony formation assays

6. How does circulating β-HB concentration in ketosis compare to concentrations in adipocyte-conditioned medium?

Answer: As the reviewer mentioned later, basal circulating level of β-hydroxybutyrate is less than 0.1 mM, but begins to rise to 1 mM after few hours of fasting (Cahill and Veech, 2003; Laffel, 1999). The concentration of β-hydroxybutyrate in the conditioned medium from MGDAs is in sub-mM range (as shown in the original Figure 4C, the concentration of β-hydroxybutyrate is equivalent to 0.2~0.8 mM).

Original Figure 4C

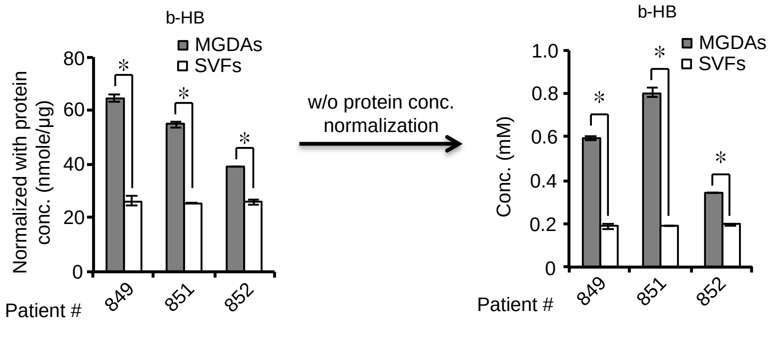

Original Figure S4C: Secretion levels of β-hydroxybutyrate in conditioned medium from MGDAs and stromal vascular fraction (SVF) cells were determined by ELISA analyses

7. **β-HB is known to be mainly produced by the liver. The concentration of circulating blood β-HB rises from ~0.1 mM observed in normal fed state to ~1 mM after few hours of fasting, and up to 5-7 mM after prolonged starvation (Cahill, 1970; Robinson and Williamson, 1980; Laffel, 1999; Cahill and Veech, 2003). Does fasting promote breast cancer progression? That would be expected if the claims in the manuscript are true. Ketone supplementation decreases tumor cell viability and prolongs survival of mice with metastatic cancer (Int J Cancer. 135(7): 1711-1720, 2014). How do authors reconcile this observation?**

Answer: As reported, β-hydroxybutyrate is mainly synthesized in the liver of mammals. However, we found that β-hydroxybutyrate was also secreted by mammary gland-derived adipocytes. In our *in vitro* co-culture system, adipocytes constantly secreted β-hydroxybutyrate and promoted breast cancer tumor growth in a MCT2-dependent manner. Moreover, daily intra-peritoneal (i.p.) administration of β-hydroxybutyrate in mouse xenograft models also promoted tumor growth in the presence of MCT2. Without MCT2 expression, the tumorigenicity of breast cancer was not enhanced despite treating with high level of β-hydroxybutyrate (10 mM), suggesting that MCT2 played a pivotal role in β-hydroxybutyrate-mediated breast tumorigenicity promotion. This study mainly focused on the microenvironment of breast cancer cells.

In the state of fasting, the elevation of circulating blood β-hydroxybutyrate is concomitant with the decrease of blood glucose. Since tumor growth is directly correlated with blood glucose levels (Seyfried et al., 2003), decrease of blood glucose level, but not elevation of blood β-hydroxybutyrate, resulted from calorie restriction serves as the basis for cancer prevention by nutritional interventions. Thus, fasting generates a much more complicated effect than just simply increasing β-hydroxybutyrate on tumor progression.

As the study referred by the reviewer (Int J Cancer. 135(7): 1711-1720, 2014), ketone supplementation decreases brain tumor cell viability in *vitro*, and prolongs survival of mice with brain metastatic tumor *in vivo*. In this study, the author also demonstrates that the decreased blood glucose resulted from ketone supplementation and calorie restriction correlates with longer survival in brain tumor bearing mice, suggesting that decrease of blood

glucose level may also contribute to restrict tumor growth *in vivo*, not solely resulted from the elevation of circulating β-hydroxybutyrate. In this situation, brain tumor cells are starved due to their heavy reliance on glucose consumption as the major energy source, therefore, prolongs survival of mice with brain metastatic tumor *in vivo*. This explanation is consistent with the finding that ketogenic diet without calories restriction does not reduce tumor growth (Fearon et al., 1985; Seyfried et al., 2003). Thus, reduced glucose is the key factor to restrict tumor progression despite in the presence of high level of circulating ketone bodies.

In our study, we observed that addition of β-hydroxybutyrate promoted breast tumor growth in a MCT2-dependent manner in a mice xenograft model. In those mice fed with regular chow diet *ad libitum*, the blood glucose level was not changed, as shown in Rebuttal Figure 1A. This finding was novel and did not contradict to the above reported observation. Moreover, as shown in Rebuttal Figure 1B, we observed that *in vitro* treatment of β-hydroxybutyrate did not reduce the proliferation of breast cancer cells, suggesting that the response to β-hydroxybutyrate was varied between brain and breast cancer cells *in vitro*. Based on the above analyses, a plausible explanation to this apparent discrepancy should be at the decreased blood glucose and different response to β-hydroxybutyrate treatment between brain and breast cancer cells. However, direct exploration of this detailed mechanism remained to be done.

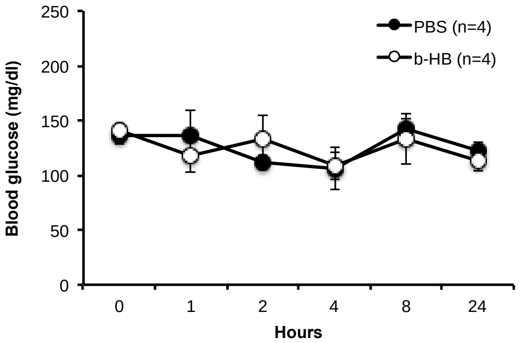

Rebuttal Figure 1A: Blood glucose levels after intraperitoneal administration of β-hydroxybutyrate in mice. Blood glucose levels were determined at the indicated time following the administration of β-hydroxybutyrate (500 mg/kg/). (PBS group, n=4; β-HB group n=4). Data show means ± s.d.

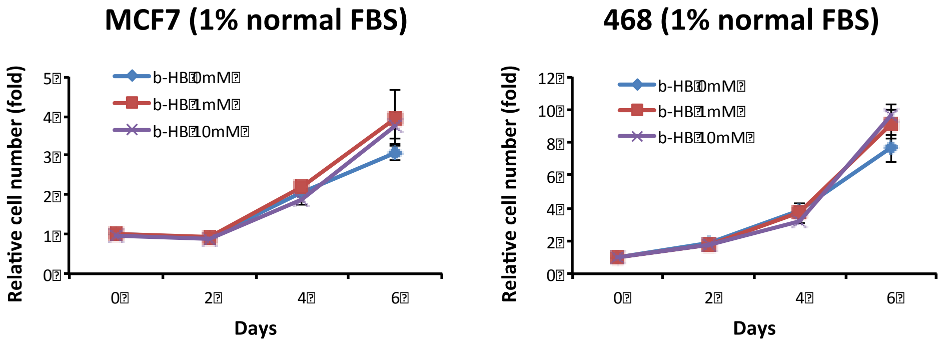

Rebuttal Figure 1B: Proliferation of breast cancer cells was not influenced upon β-hydroxybutyrate treatment The relative proliferation rate was determined by MTT assay upon different dosages of β-hydroxybutyrate treatment.

8. **The mechanism proposed in the study is novel. However, it is not sufficiently substantiated by data. While there are reports that β-HB serves as an inhibitor of class I histone deacetylases (HDACs) (Shimazu et al., 2013), it is certainly not its main function. β-HB is a ketone body used as a preferred energy source for myocytes and other cell types. It is possible that it could be used as fuel by cancer cells. Without investigating this more apparent mechanism thoroughly, the manuscript does not stand.**

Answer: We thank the reviewer's suggestion. β-hydroxybutyrate can be used as the alternative energy source for the brain and muscles during fasting or prolonged exercise (Newman and Verdin, 2014). While the cells uptake β-hydroxybutyrate from the blood, it can be reconverted into acetyl-CoA, and then used as a fuel for citric acid cycles in mitochondria. To test whether β-hydroxybutyrate can fuel breast cancer cells, we evaluated the mitochondrial bioenergetics profiles in breast cancer cells upon β-hydroxybutyrate treatment by measuring their oxygen consumption rates (OCR), indicative of mitochondrial respiration during oxidative phosphorylation, by Seahorse extracellular flux analyzer XFe96. As shown in Rebuttal Figure 2, supplement of different concentrations of pyruvate efficiently increased

OCR in a dose-dependent manner, suggesting that pyruvate could be an efficient fuel for mitochondrial respiration. However, treatment of β-hydroxybutyrate failed to do so, indicating that β-hydroxybutyrate was not the preferential fuel for mitochondria in breast cancer cells.

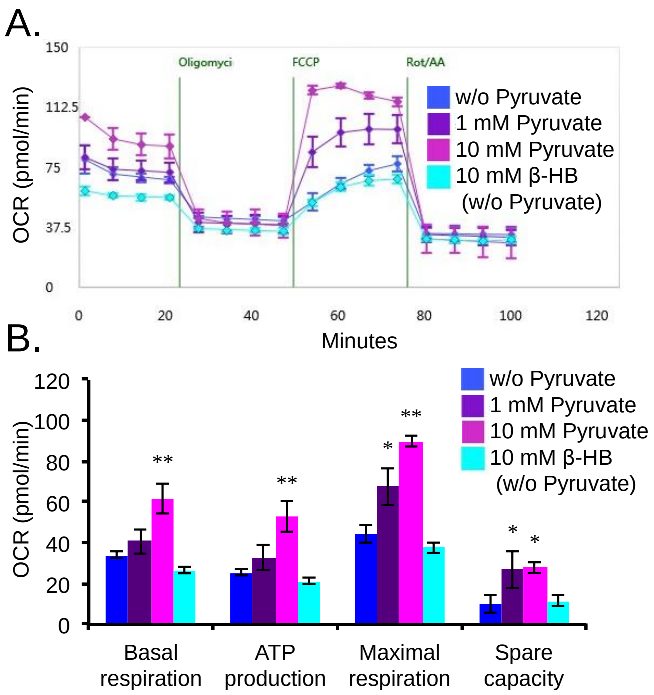

Rebuttal Figure 2: β-hydroxybutyrate would not be a preferential mitochondrial fuel in breast cancer cells. (A) Raw data of oxygen consumption rate (OCR) determined by the Seahorse XFe96 analyzer. MDA-MB-231 cells were seeded in pyruvate free condition. Different concentrations of pyruvate or β-hydroxybutyrate were added and then incubated for 1 hr before Seahorse measurement. (B) Analysis of the Seahorse data. Pyruvate, but not β-hydroxybutyrate efficiently increased basal respiration, ATP production, maximal respiration, and spare capacity of MDA-MB-231 cells. n = 3 per group; Data show means ± s.d. *, $p < 0.05$; **, $p < 0.01$ (Student's t-test).

There are also issues with data.

9. In Fig. 5, pyruvate (ketone body/ nutrient) and butyrate (HDAC inhibitor) should be used as controls.

Answer: We thank the reviewer's suggestion. As shown in Rebuttal Figure 3, treatment of pyruvate failed to induce H3K9 acetylation. However, treatment of butyrate prominently

induced H3K9 acetylation regardless of MCT2 expression in MDA-MB-231 breast cancer cells.

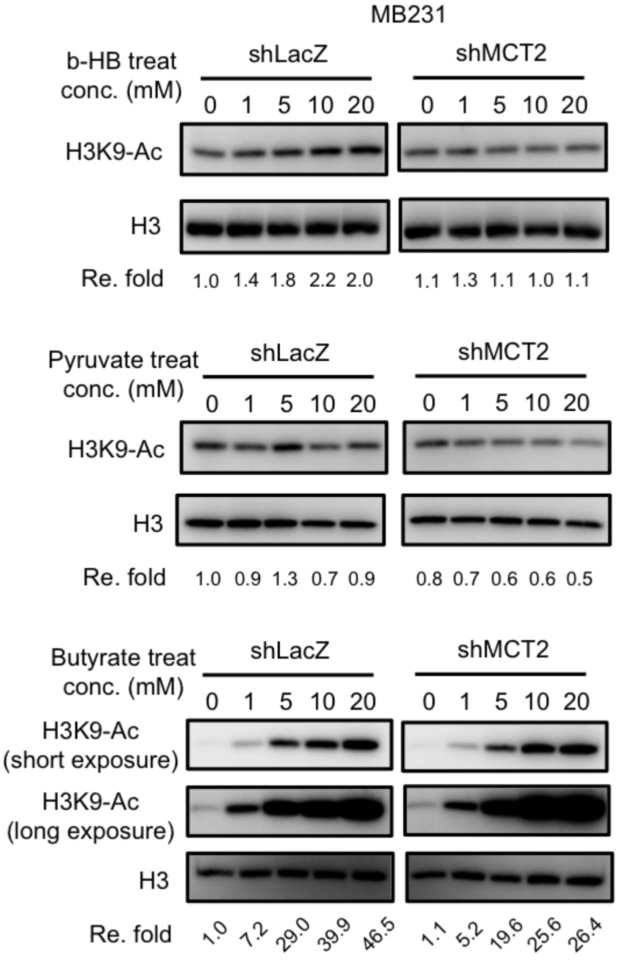

Rebuttal Figure 3: Treatment of pyruvate failed to increase H3K9 acetylation. The levels of H3K9 acetylation were assessed upon β-hydroxybutyrate, pyruvate, and butyrate treatment for 8h using a variety of doses in MDA-MB-231 breast cancer cells.

10. Supplementary Fig. S1A, image resolution is too low. MGDAs purity needs to be presented.

Answer: We thank the reviewer's suggestion. Original Figure S1A was replaced with Rebuttal Figure 4A and 4B, and the MGDAs purity was analyzed by FACS shown in Rebuttal Figure 4B (also shown as new Figure S1A and S1B in revised main text).

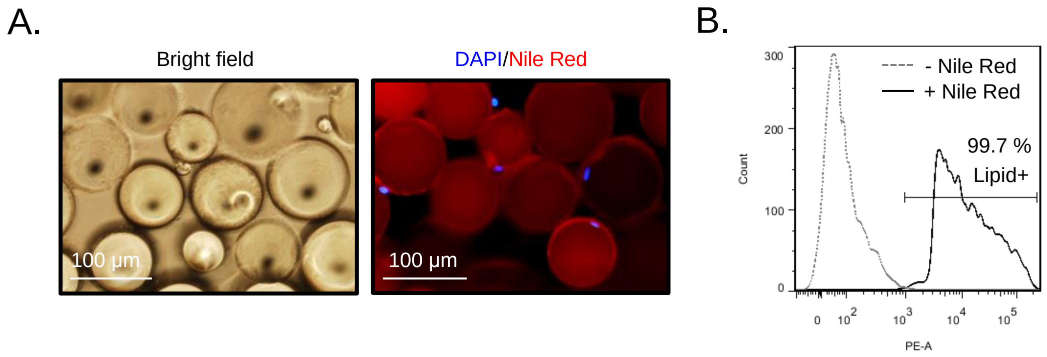

Rebuttal Figure 4: Characterization of isolated MGDA. (A) Representative images of primary MGDAs isolated from breast cancer clinical specimens. The MGDAs were double-stained with Nile red and DAPI nuclear stain (Bar, 100 μm). (B) The purity of isolated MDGAs was characterized by Nile red/DAPI double staining and analyzed by FACS.

11. In Fig 1B and C, adipocytes from healthy women would be important to show as a control.

Answer: We understood that healthy controls are important for our experiments. However, there is an ethic issue to obtain adipocytes from healthy women. Adipocytes derived from breast reduction surgery may not be considered as normal as we wish. Thus, the adipocytes derived from mastectomy of breast cancer patients with different breast cancer types showed identical effects in a MCT2-dependent manner, suggesting that the bias from adipocyte sources can be excluded.

12. GPC6 expression is the highest in ER-positive breast cancer cell lines, but not expressed in ER-negative MDA-MB-468 cell line (Fig. 3s). It has been reported that GPC6 enhances migration and invasion of breast cancer cells [Biochem J. 2011 Nov 15; 440(Pt 1): 157-166]. Depletion of GPC6 abolished the colony number increase (Fig. 3). However, there was no data to exclude GPC6 effects in clone formation.

Answer: We apologize for this missing information. As shown in Rebuttal Figure 5, we excluded GPC6 due to the high expression level in SKBR3 cells (for which no significant effect was observed when co-cultured with MGDAs). This data was incorporated into the new Figure 2D in this revised manuscript.

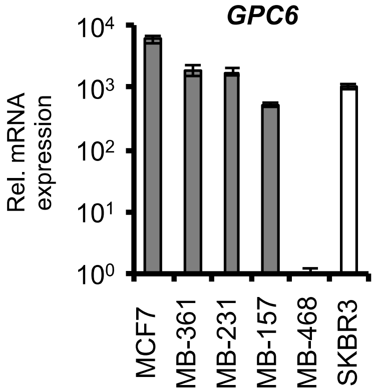

Rebuttal Figure 5: Q-PCR analysis of GPC6 in six different breast cancer cell lines.

13. Fig. 3 lacks ARMCX1, ENPP1 and MCT2 ectopically expressing data in mRNA or protein level.

Answer: We thank the reviewer's suggestion. ARMCX1, ENPP1 and MCT2 ectopically expressing data were incorporated into the new Figure 3A in this revised manuscript (also shown as Rebuttal Figure 6 herein). Overexpression of ARMCX1 in SKBR3 or MB-468 cells was measured by qRT-PCR since commercially available antibodies against ARMCX1 including ab129591 from Abcam and H00051309-M01 from Abnova both failed to detect a specific band by western blot.

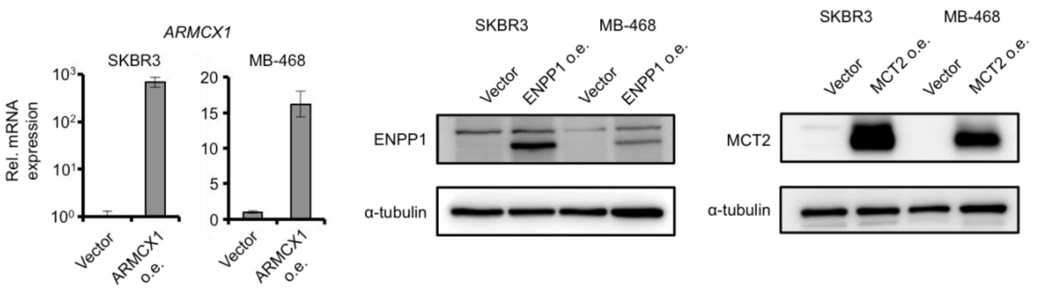

Rebuttal Figure 6: Q-PCR and western blot analyses of ARMCX1, ENPP1 and MCT2 in ARMCX1, ENPP1 and MCT2 overexpressing breast cancer cells.

14. In colony formation assay, culture medium contains 1mM pyruvate. This condition may conceal the effect of pyruvate (Figure S3 D). It was reported that Pyruvate fuels mitochondrial respiration and proliferation of breast cancer cells. Biochem J. 15;444 (3):561-71, 2012. Pyruvate-free medium should be used in this assay.

Answer: We thank the reviewer's suggestion. As shown in the original Figure S3D, we have done the colony formation assay without the supplement of pyruvate and found that deprivation of pyruvate dramatically abolished the colony formation. Consistent with previous report that pyruvate was important for supporting proliferation of breast cancer cells (Diers et al., 2012), our result demonstrated that pyruvate was also essential for supporting the growth of breast cancer cells in anchorage-independent condition. Since the colony formation of MCT2-expressing breast cancer cells shown in the original Figure 1 and 3 was further enhanced by co-culturing with MGDAs in the medium already containing 1 mM pyruvate, simply supplementing with higher concentration of pyruvate did not further promote colony formation (Figure S3C), pyruvate was less likely playing a key role in promoting colony formation of breast cancer cells mediated by MGDAs in a MCT2-dependent manner.

Original Figure S3C

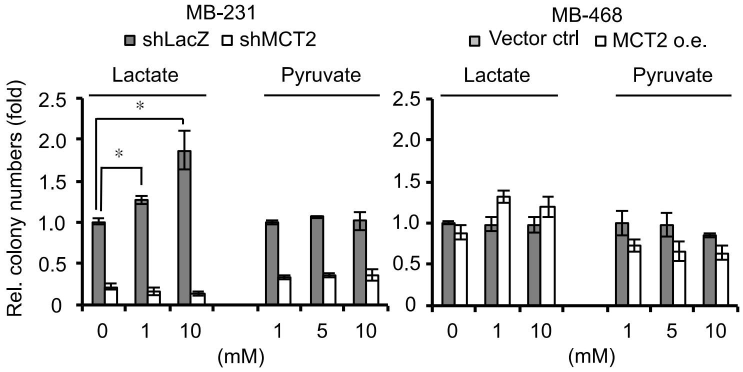

Original Figure S3C: MCT2-depleted MDA-MB-231 and overexpressing MDA-MB-468 cells were treated with various doses of lactate and pyruvate in soft agar colony formation assays

15. Pyruvate or b-HB analogs such as butyrate, amy-butyrate etc. should be used as controls for in vivo tumor studies (Fig. 4).

Answer: Butyrate is a multi-potent compound that not only serves as an energy source for intestinal epithelial cells, but also functions as a differentiating agent and HDAC inhibitor in cultured mammalian cells (Davie, 2003). To test whether butyrate exerts a similar function as β-hydroxybutyrate in breast cancer cells, we performed soft agar colony formation assay with a variety concentrations of butyrate in MDA-MB-231 cells. As shown in rebuttal Figure 7A, we found that treatment of low dose butyrate (0.1 mM) promoted colony formation, while

high dose of butyrate treatment (1 and 10 mM) showed an opposite effect. The similar bell-shaped response was also observed by Singh *et al*. and Donohoe *et al*. Low dose of butyrate treatment (0.5 mM) promoted proliferation and reduced apoptosis of colorectal cancer cells under glucose withdrawal condition (Donohoe et al., 2012; Singh et al., 1997).

Through transcriptome profiling, Donohoe *et al.* further demonstrated that low dose of butyrate treatment (0.5 mM) preferentially upregulated proliferation genes, while high dose (5 mM) exhibited reciprocal pattern with enrichment for genes associated with cell death (Donohoe et al., 2012). These results suggest that butyrate may not function equally to β-hydroxybutyrate, because treatment of high dose of β-hydroxybutyrate does not inhibit breast cancer growth *in vitro* and *in vivo*. One possibility to explain the different responses between high doses of β-hydroxybutyrate and butyrate treatment was that β-hydroxybutyrate was a relative weak HDAC inhibitor. High dose β-hydroxybutyrate treatment may mimic the low dose of butyrate treatment condition. Due to the complexity of butyrate treatment, we chose pyruvate as a control for the xenograft test. As shown in rebuttal Figure 7B, administration of pyruvate into mice failed to promote the growth of MCT2-expressing MDA-MB-231 tumor, consistent with the *in vitro* results in our study.

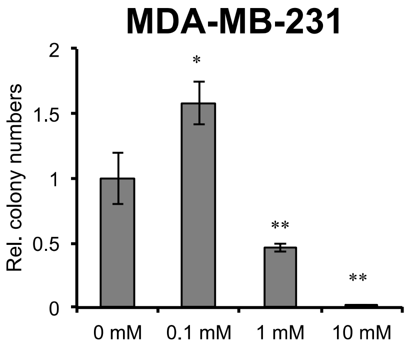

Rebuttal Figure 7A: MDA-MB-231 cells were treated with various doses of butyrate for soft agar colony formation assays. Data show means ± s.d. *, $p < 0.05$; **, $p < 0.01$ (Student's t-test).

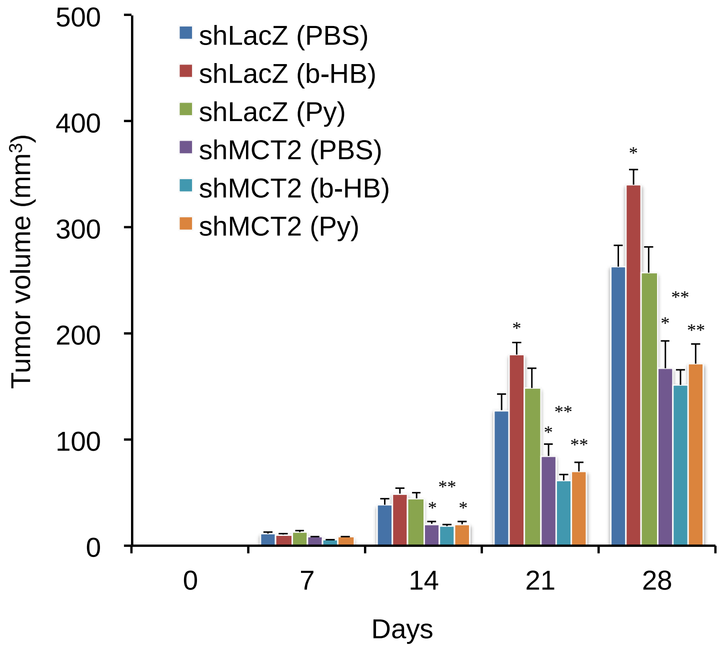

Rebuttal Figure 7B: Tumor growth assays in NOD/SCID/γ^{null} mice. Control or MCT2-depleted MDA-MB-231 cells was subcutaneously injected into both flanks of each mouse, and then the mice were administered with PBS alone, PBS containing β-hydroxybutyrate or pyruvate (500mg/kg) through daily intraperitoneal injection. The tumor volumes were measured every seven days. Six mice (n=12) were used for each group. Data show means ± S.E.M. *, $p < 0.05$; **, $p < 0.01$ (Student's t-test). b-HB, β-hydroxybutyrate; Py, Pyruvate.

16. Inhibition of HDAC by β-HB was correlated with global changes in transcription. IL-1 may not be a direct target of b-HB. High glucose also induces IL-1 in cell culture.

Answer: Treatment of β-hydroxybutyrate induced global changes in transcription including the upregulation of IL-1β. However, this did not indicate that IL-1β would be a direct target. The description was rephrased in the revised main text as follows: "Chromatin immunoprecipitation (ChIP) analysis of the IL-1β and LCN2 promoters with six primer pairs spanning the promoter regions for each promoter revealed increased histone H3K9 acetylation of the IL-1β, but not LCN2, promoter regions after β-hydroxybutyrate treatment for 1h (Figure 5F). However, the induction of LCN2 expression may be a SECONDARY effect, because upregulation of LCN2 occurred later than IL-1β (Figure 5G)."

17. In the experimental system used, it is possible that molecules other than b-HB, such as FFA or lysophospholipids, leptin and IGF-1, stimulate breast cancer cells. Comparing the effects to those of adipocyte-derived molecules reported to stimulate cancer cells would be important.

Answer: We thank the reviewer's suggestion. As shown in Rebuttal Figure 8, we compared the effects of β-hydroxybutyrate, IGF-1, and leptin treatment in control and MCT2-depleted MDA-MB-231 cells. We found that IGF-1 promoted colony formation regardless of MCT2 expression. However, treatment of leptin only slightly increased the colony formation.

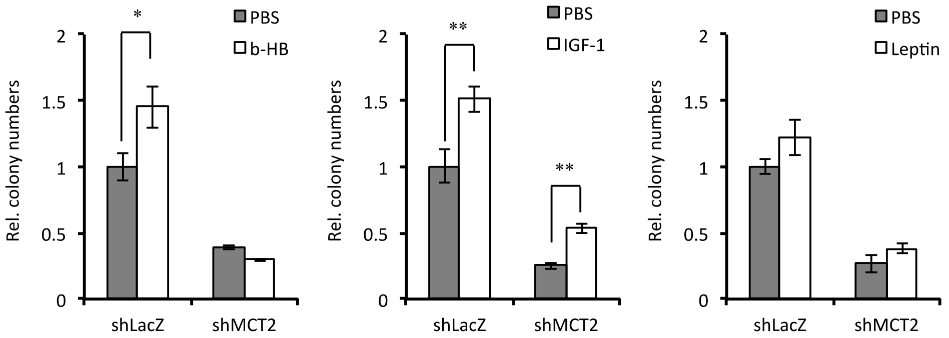

Rebuttal Figure 8: Treatment of β-hydroxybutyrate, IGF-1 and leptin in soft agar colony formation assay using MDA-MB-231 breast cancer cells. MDA-MB-231 breast cancer cells were treated with β-hydroxybutyrate (10 mM), IGF-1 (200 ng/ml), and leptin (200 ng/ml) in soft agar colony formation assay. Data show means ± s.d. *, $p < 0.05$; **, $p < 0.01$ (Student's t-test).

18. Some labels in Figs. 1, 3, 4 and 7 are missing.

Answer: We apologize for these typos. All missing labels were corrected in the revised main text.

Reviewer #2 (Remarks to the Author):

This manuscript reports that b-hydroxybutyrate secreted by mammary gland-derived adipocytes promotes the growth of breast cancer cells in vitro and in vivo and that this is dependent on the cancer cells expressing MCT2 and is mediated by upregulation of IL1b and lipocalin 2 (LCN2). LCN2 is well known to promote tumorigenesis, thus, this part is not novel. Overall it's a fairly comprehensive study, but there are numerous issues that need to be addressed.

Ans: We appreciate the suggestions from the reviewer for this work.

Specific comments:

1. **The MDA-MB-361 cell line is not an ER+ line, but a luminal HER2+. There are many real ER+ cell lines to be used like T47D and ZR-75-1.**

Answer: MDA-MB-361 is an ER-positive cell line of which the proliferation rate is stimulated upon estrogen treatment, but inhibited upon Tamoxifen treatment, respectively (Kim et al., 2016; Reddel et al., 1985).

2. The concentration of b-hydroxybutyrate needed to stimulate the growth of breast cancer cells is very high - 1-10mM. Is this anywhere close to the physiologic range in the mammary gland?

Answer: In humans, the basal level of circulating β-hydroxybutyrate is less than 0.1 mM, but begins to rise to 1 mM after fasting for few hours (Cahill and Veech, 2003; Laffel, 1999). Although we do not know the physiologic concentration of β-hydroxybutyrate in the mammary gland, the concentration of β-hydroxybutyrate in the conditioned medium from MGDAs is in sub-mM range (as shown in the original Figure 4C, the concentration of β-hydroxybutyrate is equivalent to 0.2~0.8 mM without protein concentration normalization). We believe that the local concentration of β-hydroxybutyrate in breast cancer cells surrounded by adipocytes in the mammary gland is likely higher than that in the conditioned medium.

Original Figure 4C

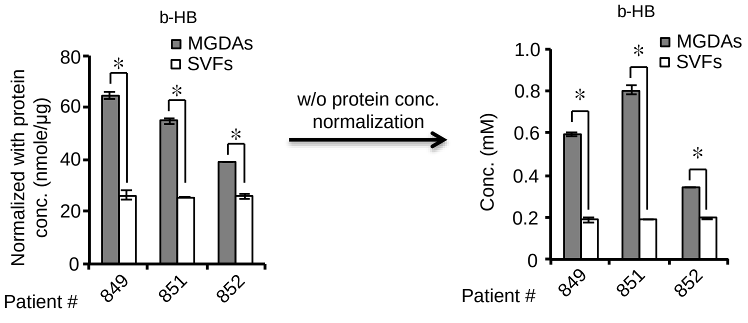

Original Figure S4C: Secretion levels of β-hydroxybutyrate in conditioned medium from MGDAs and stromal vascular fraction (SVF) cells were determined by ELISA analyses

3. MCT2 is also known as a key transporter of pyruvate and lactate - both of which are highly relevant to tumor growth. Can the authors exclude the possibility that these metabolites also play a role in adipocyte-mediated tumor promotion?

Answer: We have individually tested the effect of pyruvate, lactate, and β-hydroxybutyrate in soft agar colony formation assay. As shown in the original Figure 4D and S3C, we observed that β-hydroxybutyrate and lactate enhanced colony formation of MDA-MB-231 cells, but not MCT2-knockdown MDA-MB-231 cells. Supplementing with β-hydroxybutyrate, but not pyruvate or lactate, was also observed to significantly enhance colony formation in MCT2-overexpressing MDA-MB-468 cells (Figure 4D and S3C). Moreover, administration of β-hydroxybutyrate via daily intra-peritoneal (i.p.) injection in mouse xenograft models promoted MCT2-expressing tumor growth. Thus, β-hydroxybutyrate play a more important role than lactate and pyruvate in MCT2- and MGDAs-mediated promotion of tumor progression.

Original Figure 4D

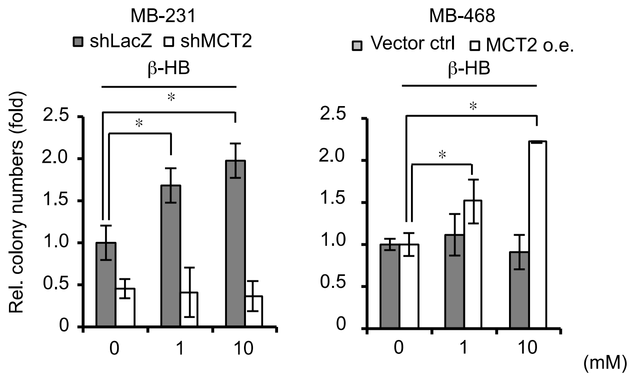

Original Figure 4D: MCT2-depleted MDA-MB-231 and overexpressing MDA-MB-468 cells were treated with various doses of β-hydroxybutyrate in soft agar colony formation assays

Original Figure S3C

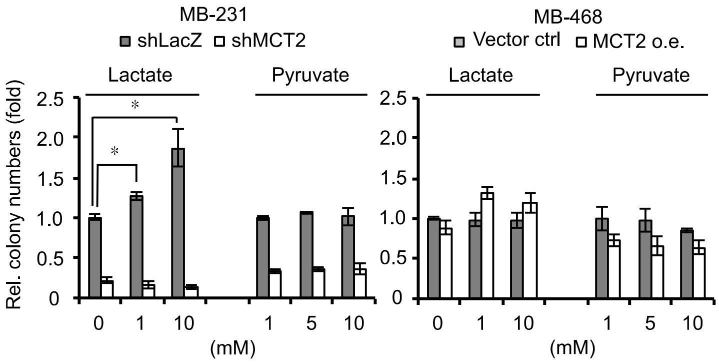

Original Figure S3C: MCT2-depleted MDA-MB-231 and MCT2-overexpressing MDA-MB-468 cells were treated with various doses of lactate and pyruvate in soft agar colony formation assays

4. There are small molecule inhibitors of MCT proteins. It would be useful to try some of these in a larger panel of breast cancer cell lines to see if this mimics the MCT2 shRNA effects.

Ans: We thank the reviewer's suggestion. As shown in Rebuttal Figure 9, the effect of β-hydroxybutyrate in colony promotion was abolished when MDA-MB-231 and MCF7 cells were treated with 1 mM CHC, a monocarboxylate transporter inhibitor.

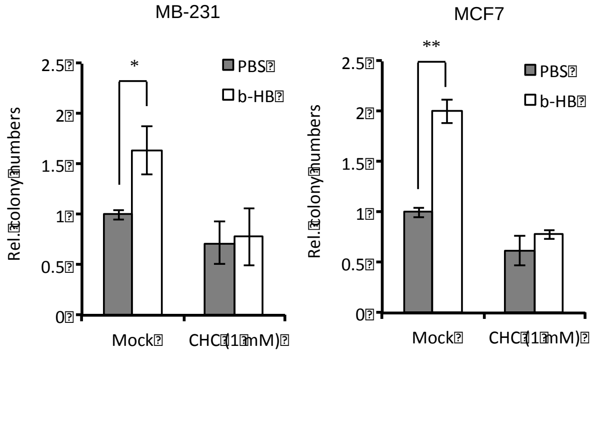

Rebuttal Figure 9: Treatment of CHC, a monocarboxylate transporter inhibitor, abolished the effect of β-hydroxybutyrate in colony promotion. MDA-MB-231 and MCF7 breast cancer cells were treated with PBS or 10 mM β-hydroxybutyrate, and then 1 mM CHC was added to investigate its effect on colony formation. Data show means ± s.d. *, $p < 0.05$; **, $p < 0.01$ (Student's t-test).

5. What is the half life and PK characteristics of b-hydroxybutyrate in the mouse? Can 1-10mM levels be achieved in the tumors by intraperitoneal injection?

Ans: As shown in Rebuttal Figure 10, the serum level of β-hydroxybutyrate was elevated at 5 min after intraperitoneal injection of β-hydroxybutyrate (500 mg/kg) and reached maximal level (~ 3 mM) at 15 min.

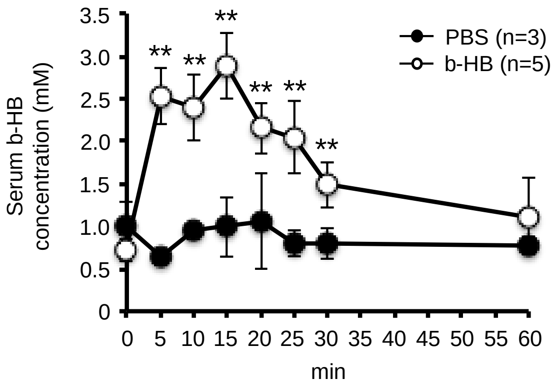

Rebuttal Figure 10: Pharmacokinetics of β-hydroxybutyrate after intraperitoneal injection in mice. The serum level of β-hydroxybutyrate was determined by ELISA analysis. (PBS group, n=3; β-HB group n=5). Data show means ± s.d. *, $p < 0.05$; **, $p < 0.01$ (Student's t-test).

6. It appears that IL1b and LCN2 are essential for breast cancer growth regardless of the adipocytes - at least this is what Figure 6B shows. Thus, the authors' interpretation is not correct.

Ans: Although depletion of both IL-1β and LCN2 abolished the MDGAs-mediated colony promotion of MDA-MB-231 breast cancer cells, it also caused severe inhibition of cell growth as shown in the original Figure 6B. To avoid the severely retarded growth effect caused by the double knockdown, we used IL-1β-neutralizing antibody to treat LCN2-depleted MDA-MB-231 breast cancer cells for evaluating the colony formation. As shown in Rebuttal Figure 11, adding IL-1β antibody further reduced the colony number of LCN2-depleted MDA-MB-231 breast cancer cells co-cultured with MGDAs. These results suggest that both IL-1β and LCN2 are essential for MGDAs-mediated colony promotion. To improve this point, the original Figure 6B was replaced with Rebuttal Figure 11. The original Figure 6B was moved to supplementary data as Figure S5B.

Original Figure 6B

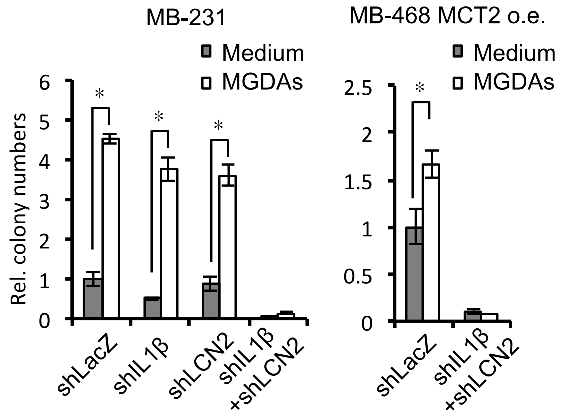

Original Figure 6B: Double knockdown of IL1β and LCN2 completely abrogated the increase of colonies induced by MGDA co-culture in MDA-MB-231 and MDA-MB-468 MCT2-overexpressing cells

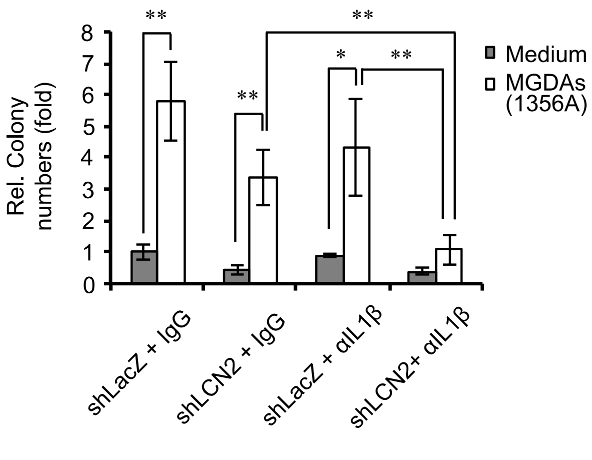

Rebuttal Figure 11: Soft agar colony formation assays using IL1β neutralizing antibody in MDA-MB-231 breast cancer cells

Soft agar colony formation assays showed that knockdown of LCN2 combining IL-1β neutralizing antibody treatment (αIL-1β, 1μg/ml) significantly abrogated the increase of colonies induced by MGDA co-culture with MDA-MB-231 cells. Mouse IgG (1μg/ml) was used as a control. Data show means ± s.d. *, $p < 0.05$; **, $p < 0.01$ (Student's t-test).

7. Both IL1b and LCN2 act through receptors - do the presence of these on breast cancer cells correlates with response to adipocytes?

Ans: As shown in Rebuttal Figure 12, IL-1R1 (IL-1β receptor) and SLC22A7 (LCN2 receptor) were detected in different breast cancer cell lines. Molt-4 and Jurkat cells were used as positive controls for IL-1R1 and SLC22A7, respectively. However, the expression of IL-1R1 and SLC22A7 did not correlate with the response to adipocytes. This was consistent with the result that the upregulation of these two receptor genes in MDGAs responsive breast cell lines was not observed in our microarray screening.

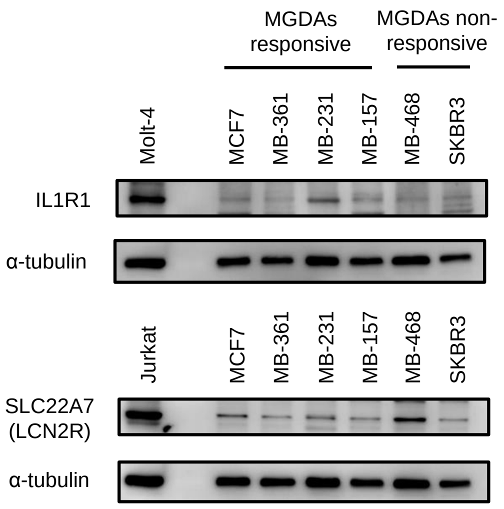

Rebuttal Figure 12: Western blots analyses of IL-1R1 (IL-1β receptor) and SLC22A7 (LCN2 receptor) in breast cancer cells.

8. **Figure 7A-B: For clinical outcome analysis ER+, HER2+, and basal-like breast tumors have to be analyzed separately, since they have very different outcomes. It is puzzling how the authors can get such significant p values in multivariate analysis using such a small and highly heterogeneous cohort. The same criticism is true for the survival data in Figure 7F.**

Ans: We thank the reviewer's suggestion. As shown in Rebuttal Figure 13, we analyzed the association between MCT2 gene expression and the survival rate in each type of breast cancer patients, including ER+, HER2+, and triple negative ones. The result indicated that higher expression of MCT2 correlated with worse survival rate among different types of breast cancer patients, suggesting that MCT2 was actually an independent prognostic factor. Consistently, a significant *p* value was also obtained by using multivariate analysis, which was commonly used to adjust the effect from other parameters.

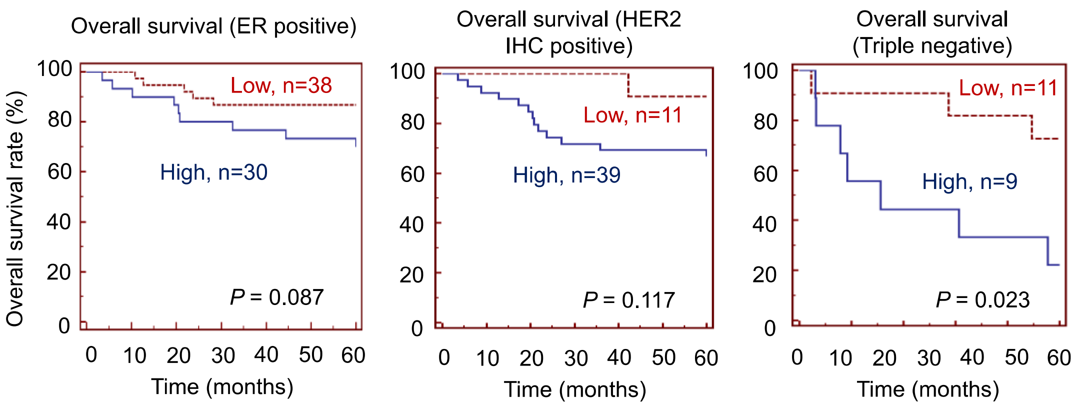

Rebuttal Figure 13: Comparison analysis between cumulative survival and MCT2 expression levels in each type of breast cancer patients using Kaplan-Meier method.

9. **The immunohistochemical staining in Figure 7C seems to show myoepithelial staining in the normal breast although overall the staining quality is very poor. The authors need to provide evidence for the specificity of the antibody (use knock out or shMCT2 controls) and provide better quality images.**

Ans: We thank the reviewer's suggestion. As shown in Rebuttal Figure 14 (also shown as Figure S6 in the revised main text), the specificity of MCT2 antibody was demonstrated by

IHC staining in tumors from xenograft mouse model. Moreover, original Figure 7C was replaced with a new Figure 7C. Occasionally we can observe weak myoepithelial staining in normal breast. This may also explain why high MCT2 expression is correlated with poor prognosis in breast cancer patients, because basal type breast tumors are associated with aggressive behavior and poor prognosis.

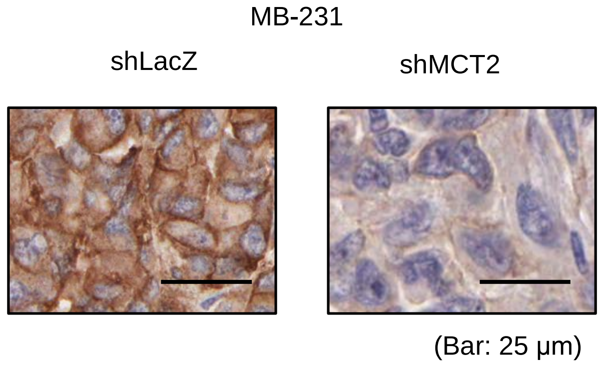

Rebuttal Figure 14: Immunohistochemistry staining of MCT2 in MDA-MB-231 shLacZ and shMCT2 xenograft tumors.

10. The differences between control and experimental groups are modest in numerous figures. It would be important to know how many replicates were included in each experiments and how many independent experiments reproduced the findings.

Ans: We apologize for this missing information. This information was corrected in Methods and Figure legends in the revised main text. That is, we performed these experiments in technical triplicate and repeated as least twice independently. Similar results were observed and one representative result was shown.

Reviewer #3 (Remarks to the Author):

In the present study, the authors describe the role of MCT2 in promoting breast cancer malignancy via crosstalk with mammary gland-derived adipocytes.

The results are original, with interest to scientists in the cancer field and, importantly, could have potential clinical application.

The manuscript is presented in a clear way, with a good abstract and introduction. The experimental approaches are adequate; the quality of data is good, the authors provide strong evidence for their conclusions and references are adequate.

Ans: We appreciate the kindest encouragement from the reviewer for this work.

Suggested improvements:

1. **Fig.2D. Western-blot results should be also presented for ARMCX1 gene.**

Ans: We have tested two commercially available antibodies against ARMCX1 including ab129591 from Abcam and H00051309-M01 from Abnova, but both failed to detect a specific band by western blot.

2. **Fig. 2. Since the function of MCT2 as a plasma membrane transporter is being investigated, the authors should also present evidence for its presence at the plasma membrane (IF/cell fractionation/...) of the cell lines.**

Ans: We thank the reviewer’s suggestion. As shown in Rebuttal Figure 15 (also shown as Figure S7 in the revised main text), a cell membrane staining of MCT2 was observed in breast cancer cells by immunofluorescence assay.

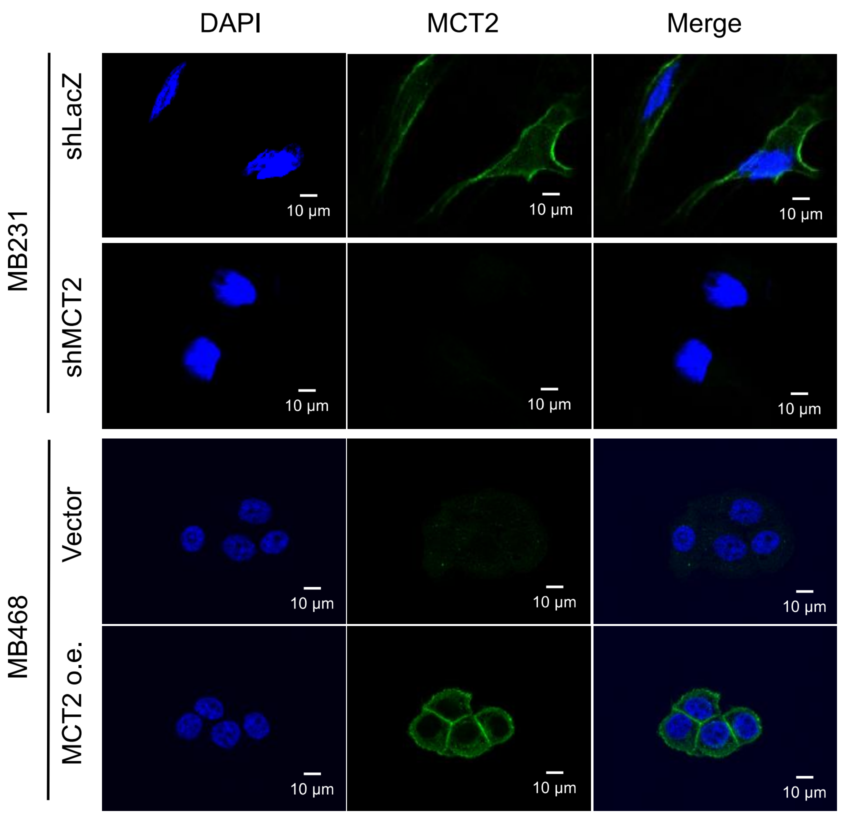

Rebuttal Figure 14: Immunofluorescence staining of MCT2 in breast cancer cells. The intracellular location of MCT2 was determined by IF staining in MDA-MB-231 (Upper panel, shLacZ vs. shMCT2) and MDA-MB-468 breast cancer cells (Lower panel, vector control vs. MCT2 o.e.). Bar, 10 μm

3. **Results. Description of Fig. 3B. The sentence "... only MCT2, but not ARMCX1 nor ENPP1, enhanced colony formation (Figure 3B)" should be rephrased since there is also enhancement of colony formation for ENPP1 in SKBr3 cells.**

Ans: We apologize for this confusion. The description was rephrased in the revised main text. “As shown in Figure 3C only MCT2, but not ARMCX1 nor ENPP1, consistently enhanced colony formation in both MDA-MB-468 and SK-BR3 cells.”

4. **Figure 6C. Are the squares in the figure supposed to be there? Please check.**

Answer: We apologize for these typos. These errors were corrected in the revised main text.

5. **The cutoff for MCT2 positivity in clinical cases was considered as 5% of membrane staining and only 7/36 cases were presented positive. Why did the authors choose 5%? What is the biological significance of 5% positivity in a tissue sample? This cutoff should be justified.**

Ans: We thank the reviewer for suggestion. Empirically, 5% is a widely acceptable cut-off value (Chu and Weiss, 2002; Lee et al., 2009). To further verify this criterion in our cohort, we performed receiver operating characteristic (ROC) analysis by MedCalc statistical software shown in Rebuttal Figure 16 to determine the optimal cut-off value of MCT2-positive staining for disease free survival correlation analysis, and the result showed that the cut-off value must be great than 0%. Thus, we believe that 5% positivity is also acceptable here in our study.

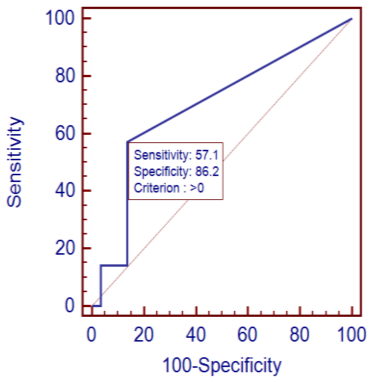

Criterion	Sensitivity	95% CI	Specificity	95% CI
>=0	100.00	59.0 - 100.0	0.00	0.0 - 11.9
>0 *	57.14	18.4 - 90.1	86.21	68.3 - 96.1
>5	28.57	3.7 - 71.0	86.21	68.3 - 96.1
>30	14.29	0.4 - 57.9	86.21	68.3 - 96.1
>40	14.29	0.4 - 57.9	93.10	77.2 - 99.2
>55	14.29	0.4 - 57.9	96.55	82.2 - 99.9
>60	0.00	0.0 - 41.0	96.55	82.2 - 99.9
>100	0.00	0.0 - 41.0	100.00	88.1 - 100.0

Rebuttal Figure 16: ROC (receiver operating characteristic) analysis for optimal cut-off value of MCT2 positive IHC staining selection for disease free survival correlation analysis.

6. **With exception of 231 cells, the breast cancer cells used also express MCT1, which is also able to transport β-hydroxybutyrate. Why is not MCT1 involved in β-hydroxybutyrate transport in breast cancer cells? Is it a matter of concentration, owing to the different affinities of the transporters? This issue should be discussed.**

Ans: We thank the reviewer's suggestion. In general, MCT2 shows higher affinity (about 10-fold increase) to most monocarboxylates than MCT1 (Halestrap and Meredith, 2004). Among the breast cancer cell lines used in this study, MDA-MB-468 cells express relatively higher level of MCT1 than the others (Rebuttal Figure 17A, also shown as Figure S8 in the revised main text). However, treatment of β-hydroxybutyrate failed to promote the colony numbers of MDA-MB-468 cells. Ectopic expression of MCT1 also failed to rescue the response to β-hydroxybutyrate supplement in MCT2-depleted MDA-MB-231 cells, suggesting that MCT2 played a more important role in promotion of breast tumor progression (Rebuttal Figure 17B and 17C).

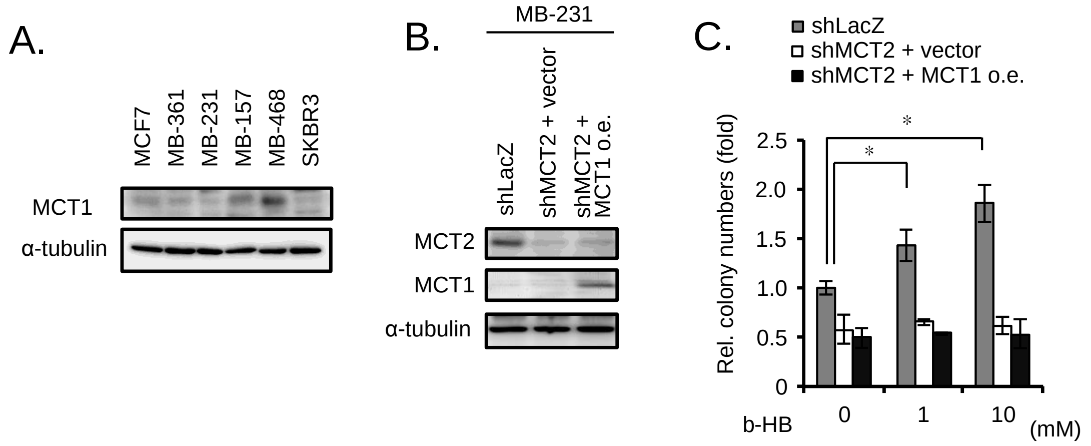

Rebuttal Figure 17: Ectopic expression of MCT1 failed to rescue the response to β-hydroxybutyrate supplement in MCT2-depleted MDA-MB-231 cells. (A) Western blot analysis of MCT1 in six different breast cancer cell lines. (B) Overexpression of MCT1 in MCT2-depleted MDA-MB-231 cells. (C) Ectopic expression of MCT1 in MCT2-depleted MDA-MB-231 cells did not rescue the response to β-hydroxybutyrate treatment in soft agar colony formation assays. Data show means ± s.d. *, $p < 0.05$ (Student's t-test).

References:

1. Cahill, G. F., Jr., and Veech, R. L. (2003). Ketoacids? Good medicine? Trans Am Clin Climatol Assoc *114*, 149-161; discussion 162-143.
2. Chu, P. G., and Weiss, L. M. (2002). Expression of cytokeratin 5/6 in epithelial neoplasms: an immunohistochemical study of 509 cases. Mod Pathol *15*, 6-10.
3. Davie, J. R. (2003). Inhibition of histone deacetylase activity by butyrate. J Nutr *133*, 2485S-2493S.
4. Diers, A. R., Broniowska, K. A., Chang, C. F., and Hogg, N. (2012). Pyruvate fuels mitochondrial respiration and proliferation of breast cancer cells: effect of monocarboxylate transporter inhibition. The Biochemical journal *444*, 561-571.
5. Donohoe, D. R., Collins, L. B., Wali, A., Bigler, R., Sun, W., and Bultman, S. J. (2012). The Warburg effect dictates the mechanism of butyrate-mediated histone acetylation and cell proliferation. Mol Cell *48*, 612-626.
6. Fearon, K. C., Tisdale, M. J., Preston, T., Plumb, J. A., and Calman, K. C. (1985). Failure of systemic ketosis to control cachexia and the growth rate of the Walker 256 carcinosarcoma in rats. Br J Cancer *52*, 87-92.
7. Halestrap, A. P., and Meredith, D. (2004). The SLC16 gene family-from monocarboxylate transporters (MCTs) to aromatic amino acid transporters and beyond. Pflugers Archiv : European journal of physiology *447*, 619-628.
8. Kim, J., Lee, J., Jang, S. Y., Kim, C., Choi, Y., and Kim, A. (2016). Anticancer effect of metformin on estrogen receptor-positive and tamoxifen-resistant breast cancer cell lines. Oncol Rep *35*, 2553-2560.
9. Laffel, L. (1999). Ketone bodies: a review of physiology, pathophysiology and application of monitoring to diabetes. Diabetes Metab Res Rev *15*, 412-426.
10. Lee, C. H., Turbin, D. A., Sung, Y. C., Espinosa, I., Montgomery, K., van de Rijn, M., and Gilks, C. B. (2009). A panel of antibodies to determine site of origin and malignancy in smooth muscle tumors. Mod Pathol *22*, 1519-1531.
11. Newman, J. C., and Verdin, E. (2014). Ketone bodies as signaling metabolites. Trends Endocrinol Metab *25*, 42-52.
12. Park, J., Morley, T. S., Kim, M., Clegg, D. J., and Scherer, P. E. (2014). Obesity and cancer--mechanisms underlying tumour progression and recurrence. Nat Rev Endocrinol *10*, 455-465.
13. Reddel, R. R., Murphy, L. C., Hall, R. E., and Sutherland, R. L. (1985).

Differential sensitivity of human breast cancer cell lines to the growth-inhibitory effects of tamoxifen. Cancer research *45*, 1525-1531.

14. Seyfried, T. N., Sanderson, T. M., El-Abbadi, M. M., McGowan, R., and Mukherjee, P. (2003). Role of glucose and ketone bodies in the metabolic control of experimental brain cancer. Br J Cancer *89*, 1375-1382.
15. Singh, B., Halestrap, A. P., and Paraskeva, C. (1997). Butyrate can act as a stimulator of growth or inducer of apoptosis in human colonic epithelial cell lines depending on the presence of alternative energy sources. Carcinogenesis *18*, 1265-1270.
16. Tan, J., Buache, E., Chenard, M. P., Dali-Youcef, N., and Rio, M. C. (2011). Adipocyte is a non-trivial, dynamic partner of breast cancer cells. The International journal of developmental biology *55*, 851-859.
17. Vona-Davis, L., and Rose, D. P. (2007). Adipokines as endocrine, paracrine, and autocrine factors in breast cancer risk and progression. Endocrine-related cancer *14*, 189-206.

Reviewers' comments:

Reviewer #1 (Remarks to the Author):

The authors adequately addressed the majority of the concerns. Point 8 (the possibility that β-hydroxybutyrate can be used as the alternative energy source) is very important and should be included as a display item.

For point 5, the authors misunderstood the concern. This reviewer had no issue with the conclusion that β-HB plays a role in cancer. The question is whether ADIPOCYTE-DERIVED β-HB plays a role in cancer. Points 6-7 do not convincingly address this, and point 11 had been raised to get more data related to this issue. The 5-7 mM β-HB in circulation that can be achieved simply by dietary intervention is an order of magnitude above the 0.2~0.8 mM β-HB in the adipocyte-conditioned medium as reported here. Therefore, without positive data from new experiments including adipocytes NOT secreting β-HB, the title is unacceptable. If such experiments are not possible, the authors need to reduce the title and conclusions to "β-hydroxybutyrate promotes malignant growth of breast tumor with monocarboxylate transporter 2 expression". The hypothesis that it is "secreted from adipocytes of mammary glands" should only be considered as a working model.

Finally, the discussion of glucose-related discrepancies of the data with previous cancer ketone supplementation studies should be included in the paper.

Reviewer #2 (Remarks to the Author):

The authors have responded to each of the reviewers' criticism and revised the manuscript accordingly. The revised manuscript is improved. However, several issues still remain:

1. Figure 7A-B: this figure is still the same despite the criticism. In breast cancer combining all subtypes for survival analysis is meaningless, since the subtypes differ so much for survival. They should show rebuttal Figure 13, which actually shows that the survival difference is only significant in triple negative breast cancer patients. But, this new

figure also highlights the small size of the cohort making any associations with survival not particularly convincing. The same criticism applies for Figure 7C-D: have to show survival associations within a specific subtype. Besides, association with survival following currently used therapies is not necessarily a good/bad thing for a potential new therapeutic target/biological mechanism.

2. The use of adipocytes only from breast cancer patients and not from normal reduction mammoplasties was also a prior criticism that was not addressed. Reduction mammoplasties may not be fully normal, but certainly more normal than adipocytes from breast cancer patients and testing two cultures is certainly not a significant number that can control for interindividual variability.

Reviewer #3 (Remarks to the Author):

The authors made the required changes in the manuscript. In my opinion, the manuscript is now ready for publication.

Detailed point-by-point response to reviewers' comments (bold)

Reviewer #1 (Comments to the Author):

The authors adequately addressed the majority of the concerns.

Ans: We appreciate the critical and constructive suggestions from the reviewer for this work.

Comments:

19. **Point 8 (the possibility that β-hydroxybutyrate can be used as the alternative energy source) is very important and should be included as a display item.**

Answer: We thank the reviewer's suggestion. We have included this part as discussion in the revised main text and the relevant result was shown as Figure S13.

20. **For point 5, the authors misunderstood the concern. This reviewer had no issue with the conclusion that β-HB plays a role in cancer. The question is whether ADIPOCYTE-DERIVED β-HB plays a role in cancer. Points 6-7 do not convincingly address this, and point 11 had been raised to get more data related to this issue. The 5-7 mM β-HB in circulation that can be achieved simply by dietary intervention is an order of magnitude above the 0.2~0.8 mM β-HB in the adipocyte-conditioned medium as reported here. Therefore, without positive data from new experiments including adipocytes NOT secreting β-HB, the title is unacceptable. If such experiments are not possible, the authors need to reduce the title and conclusions to "β-hydroxybutyrate promotes malignant growth of breast tumor with monocarboxylate transporter 2 expression". The hypothesis that it is "secreted from adipocytes of mammary glands" should only be considered as a working model.**

Answer: Undoubtedly, we understand that the adipocytes without β-HB secretion would be the best control to demonstrate the importance of the role of adipocytes-derived β-HB in breast cancer progression. However, such adipocytes may not possibly exist. To circumvent this difficulty, we designed an experiment using β-HB dehydrogenase (BDH) to remove β-HB in adipocytes-CM. As shown in rebuttal Figure 1A, β-HB dehydrogenase could covert β-HB into acetoacetate in the presence of cofactor NAD^+, and the BDH activity could be monitored by the production of NADH at 340 nm (OD_{340nm}). The dynamic range of BDH

catalyzing enzymatic reaction was shown in rebuttal Figure 1B. Under this condition, BDH could efficiently convert β-HB up to 8 mM, which was sufficient to convert all the β-HB in the adipocytes-CM (1 ml adipocytes-CM was catalyzed with 20 μl BDH (10U/ml) in the presence of 20 μl NAD^+ (50mM) at 37 °C for 1h). After removal of the β-HB in fractionated adipocytes-CM (< 10 kD), the activity to promote the colony number was significantly reduced (Rebuttal Figure 1C, also shown as Figure S4C in the revised main text). This result suggested that adipocytes derived β-HB had a role in cancer progression, which was consistent with the role of MCT2 in β-HB transport from extracellular space into the cells. Although 5-7 mM β-HB in circulation could be achieved by dietary intervention, most of the breast cancer patients did not use ketogenic diet as nutritional interventions or cancer adjuvant therapy, suggesting that the circulating concentration of β-HB in these patients was maintained at basal level (less than 0.1 mM). Under this situation, the importance of adipocytes-derived β-HB in breast cancer microenvironment would be revealed. Consistent with this concept, recent report demonstrated that long-term high fat ketogenic diet promoted renal tumor growth in a rat model (Liskiewicz et al., 2016).

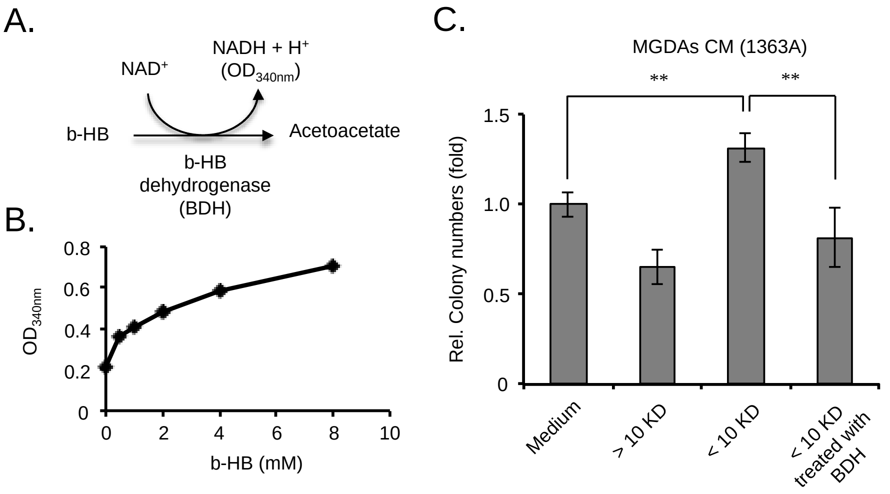

Rebuttal Figure 1: Soft agar colony formation assays using β-HB depleted adipocytes-CM.

(A) β-HB could be converted to acetoacetate by the enzyme beta-hydroxybutyrate dehydrogenase (BDH). The BDH activity could be monitored by the production of NADH at 340 nm (OD_{340nm}). (B) The dynamic range of BDH catalyzing enzymatic reaction. 0, 0.5, 1, 2, 4, and 8 mM β-HB (1ml) were converted to acetoacetate by BDH (10 U/ml, 20µl) in the presence of NAD^+ (50 mM, 20µl) at 37 °C for 1h. (C) Removal of the β-HB in fractionated adipocytes-CM (< 10 kD) decreased the promoting activity in soft agar colony formation using MDA-MB-231 breast cancer cells. Data show means ± s.d. *, $p < 0.05$; **, $p < 0.01$ (Student's t-test).

For the issue using adipocytes from healthy women, we understood that the control could have different meaning. Unfortunately, we didn't have healthy women receiving breast reduction surgery from our cooperated physicians. However, we have tested more than ten different adipocytes derived from breast cancer patients to minimize the individual genetic variability, and obtained similar results of promoting breast cancer progression (as shown in rebuttal Figure 2).

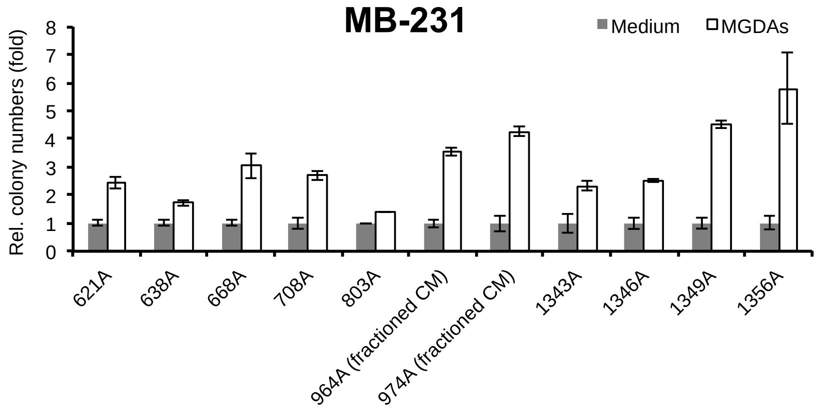

Rebuttal Figure 2: Soft agar colony formation assays using MDA-MB-231 breast cancer cells co-cultured with MGDAs or treated with CM.

21. **Finally, the discussion of glucose-related discrepancies of the data with previous cancer ketone supplementation studies should be included in the paper.**

Answer: We thank the reviewer's suggestion. We have included this part as discussion in the revised main text and incorporated the relevant data as Figure S11 and S12.

Reviewer #2 (Remarks to the Author):

The authors have responded to each of the reviewers' criticism and revised the manuscript accordingly. The revised manuscript is improved. However, several issues still remain.

Ans: We appreciate the critical and constructive suggestions from the reviewer for this work.

Specific comments:

2. **Figure 7A-B: this figure is still the same despite the criticism. In breast cancer combining all subtypes for survival analysis is meaningless, since the subtypes differ so much for survival. They should show rebuttal Figure 13, which actually shows that the survival difference is only significant in triple negative breast cancer patients. But, this new figure also highlights the small size of the cohort making any associations with survival not particularly convincing. The same criticism applies for Figure 7C-D: have to show survival associations within a specific subtype. Besides, association with survival following currently used therapies is not necessarily a good/bad thing for a potential new therapeutic target/biological mechanism.**
3.

Answer: As the issue mentioned by the reviewer, the survival probability and outcome after treatment are varied among different subtypes of breast cancer patients. Although the subtypes of breast cancer classified by ER and HER2 status potentially affect the survival time of a patient, many other clinical parameters, such as age, tumor size, and lymph node metastasis also contribute to the prognosis. To eliminate the influence of these factors on the survival analysis, the Cox model is the most commonly used and well accepted multivariate approach for survival analysis in biomedical research (Bradburn et al., 2003). As shown in

Figure 7B, the association of MCT2 expression with poor prognosis was still significant after adjustment for age, tumor size, lymph node status, grade, and ER expression using multivariate Cox regression model, suggesting that MCT2 is an independent prognostic factor in patients with breast cancer.

To strengthen our conclusion that MCT2 is a poor prognostic factor, another independent breast cancer cohort with greater size (n=327, characteristics of patients and tumors were shown in rebuttal table 2) was enrolled for survival analysis. The data was adapted from the breast tumor gene expression profiling by Kao *et al*, BMC Cancer, 2011, 11 (143), http://www.oncomine.org. As shown in rebuttal Figure 3, Kaplan-Meier (KM) analysis showed that patients with high MCT2 expression had a shorter survival time compared to patients with low MCT2 expression (p= 0.0003). High or low expression of MCT2 was determined by ROC (Receiver operating characteristic) curve analysis. Furthermore, the association of MCT2 expression with poor prognosis was significant after adjustment for age, ER, HER2, T, N, and M status using multivariate Cox regression model (as shown in rebuttal table 1). We would like to put Rebuttal Figure 3 and Rebuttal table 1&2 in the main text as Figure S6 and Table S4&S5 to share the readers because the observation is significant.

In the present study, we have demonstrated that MCT2 was not only a biomarker for poor prognosis, but also an effector playing an important role in breast cancer progression. Thus, MCT2 could be a potential new therapeutic target undoubtedly.

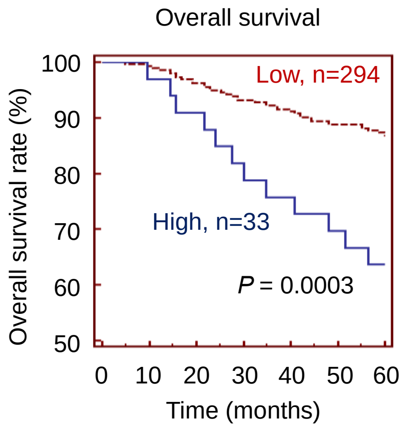

Rebuttal Figure 3: Kaplan-Meier analysis showed correlation between cumulative survival and MCT2 expression levels in breast cancer patients. (Data was adapted from the breast tumor gene expression profiling by Kao *et al*, BMC Cancer, 2011, 11 (143), http://www.oncomine.org)

Rebuttal Table 1. Univariate and multivariate proportional hazards analysis of the influence of MCT2 expression on the overall survival of 327 breast cancer patients

(Data was adapted from the breast tumor gene expression profiling by Kao *et al*, BMC Cancer, 2011, 11 (143), http://www.oncomine.org)

Variables	Univariate HR (95% CI)	*P*	Multivariate HR (95% CI)	*P*
MCT2 high-risk group (vs. low-risk group)	3.13 (1.64-5.97)	**0.0005**	2.03 (1.01-4.08)	**0.049**
Age (≥ 46 years old)[a]	0.84 (0.49-1.45)	0.5363	1.06 (0.60-1.87)	0.8510
ER (vs negative group)	0.38 (0.22-0.67)	**0.0007**	0.51 (0.26-1.02)	0.0581
HER2 (vs negative group)	2.35 (1.35-4.12)	**0.0028**	1.42 (0.74-2.70)	0.2865
T status (per grade) [b]	2.04 (1.49-2.80)	**<0.0001**	1.48 (0.95-2.32)	0.0842
N status (per grade) [c]	1.92 (1.50-2.46)	**<0.0001**	1.62 (1.22-2.14)	**0.0008**
M status (per grade) [d]	4.52 (1.63-12.50)	**0.0038**	1.92 (0.52-7.09)	0.3321

[a]Median age = 46 years old; [b]T status, T1-T4; [c]N status, N0-N3; [d]M status, M0-M1

HR: hazard ratio, CI: confidence interval

Rebuttal Table 2. Characteristics of patients and tumors

(Data was adapted from the breast tumor gene expression profiling by Kao *et al*, BMC Cancer, 2011, 11 (143), http://www.oncomine.org)

Factors	No of patients (%) n = 327
Age (years old)	
≤ 50	209 (63.9 %)
> 50	118 (36.1 %)
T status	
T1	99 (30.3 %)
T2	188 (57.5 %)
T3	26 (7.9 %)
T4	12 (3.7 %)
unknown	2 (0.6 %)
N status	
N0	137 (41.9 %)
N1	87 (26.6 %)
N2	63 (19.3 %)
N3	40 (12.2 %)
M status	
M0	
M1	319 (97.6 %)

	8 (2.4 %)
TNM stage	
I	67 (20.5 %)
II	147 (45.0 %)
III	103 (31.5 %)
IV	8 (2.4 %)
unknown	2 (0.6 %)
ER	
(+)	204 (62.4 %)
(-)	123 (37.6 %)
HER2	
(+)	75 (22.9 %)
(-)	252 (77.1%)

2. **The use of adipocytes only from breast cancer patients and not from normal reduction mammoplasties was also a prior criticism that was not addressed. Reduction mammoplasties may not be fully normal, but certainly more normal than adipocytes from breast cancer patients and testing two cultures is certainly not a significant number that can control for inter individual variability.**

Answer: We appreciate that the reviewer also points out the importance of using adipocytes from healthy women in controlling individual variability for our experiments. We understood that the control could have different meaning. Unfortunately, we didn't have healthy women receiving breast reduction surgery from our cooperated physicians. However, we have tested more than ten different adipocytes derived from breast cancer patients to minimize the individual genetic variability, and obtained similar results of promoting breast cancer progression (as shown in rebuttal Figure 2).

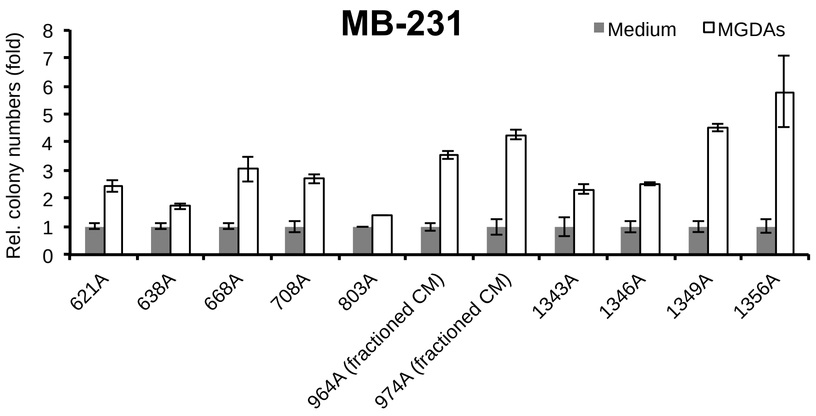

Rebuttal Figure 2: Soft agar colony formation assays using MDA-MB-231 breast cancer cells co-cultured with MGDAs or treated with CM.

Reviewer #3 (Remarks to the Author):

The authors made the required changes in the manuscript. In my opinion, the manuscript is now ready for publication.

Ans: We appreciate the kindest encouragement from the reviewer for this work.

References:

Bradburn, M. J., Clark, T. G., Love, S. B., and Altman, D. G. (2003). Survival analysis part II: multivariate data analysis--an introduction to concepts and methods. Br J Cancer *89*, 431-436.

Liskiewicz, A. D., Kasprowska, D., Wojakowska, A., Polanski, K., Lewin-Kowalik, J., Kotulska, K., and Jedrzejowska-Szypulka, H. (2016). Long-term High Fat Ketogenic Diet Promotes Renal Tumor Growth in a Rat Model of Tuberous Sclerosis. Sci Rep *6*, 21807.

REVIEWERS' COMMENTS:

Reviewer #1 (Remarks to the Author):

The authors adequately addressed the concerns.

Reviewer #2 (Remarks to the Author):

The authors did not really address the issues raised by the reviewers but rather explained why they are not addressing it. Since this is the second round of review and they repeatedly not respond properly to the request, I have no additional comments on this paper.

Detailed point-by-point response to reviewers' comments (bold)

Reviewer #1 (Remarks to the Author):

The authors adequately addressed the majority of the concerns.

Ans: We appreciate the critical and constructive suggestions from the reviewer for this work.

Reviewer #2 (Remarks to the Author):

The authors did not really address the issues raised by the reviewers but rather explained why they are not addressing it. Since this is the second round of review and they repeatedly not respond properly to the request, I have no additional comments on this paper.

Ans: It is very regretful that the reviewer still thought that the comments raised were not addressed properly. We believed that we had already addressed the major concerns raised by the reviewer as shown in the second round of the rebuttal letter as attached below.
